# Laser Cooling with an Intermediate State and Electronic Structure Studies of the Molecules CaCs and CaNa

**DOI:** 10.1021/acsomega.2c01224

**Published:** 2022-05-24

**Authors:** Amal Moussa, Nayla El-Kork, Israa Zeid, Ehab Salem, Mahmoud Korek

**Affiliations:** † Faculty of Science, Beirut Arab University, P.O. Box 11-5020, Riad El Solh, Beirut 1107 2809, Lebanon; ‡ Department of Physics, Khalifa University, P.O. Box 127788, Abu Dhabi 51133, United Arab Emirates; § Space and Planetary Science Center, Khalifa University, Abu Dhabi 51133, United Arab Emirates

## Abstract

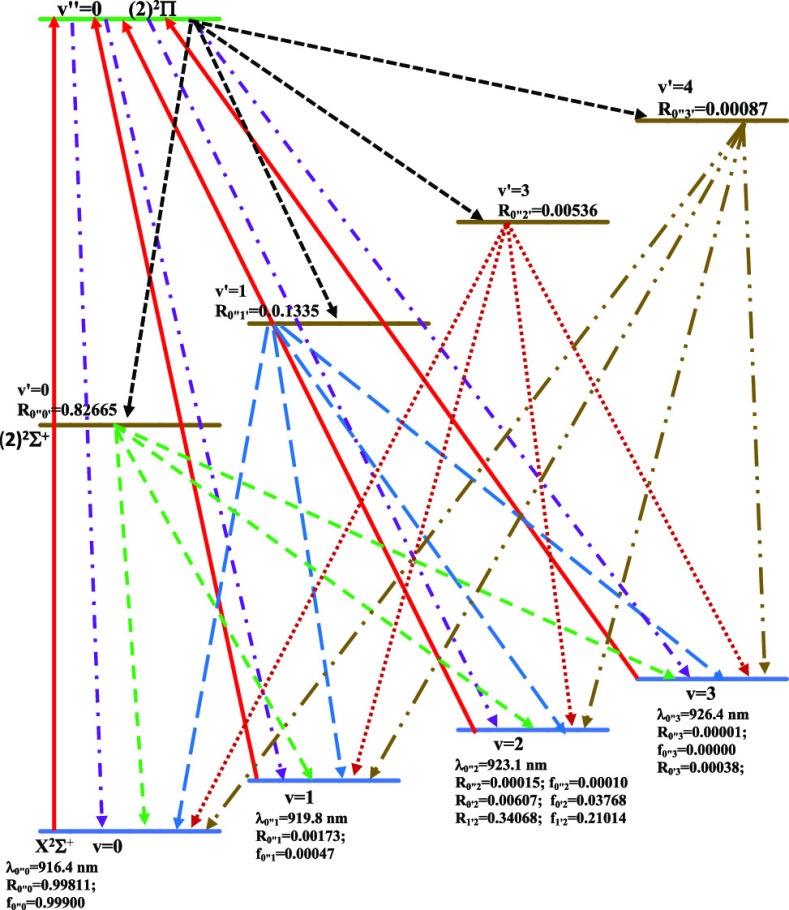

The ground and excited electronic states of the diatomic molecules CaCs and CaNa have been investigated by implementing the ab initio CASSCF/(MRCI + Q) calculation. The potential energy curves of the doublet and quartet electronic low energy states in the representation ^2*s*+1^Λ^(±)^ have been determined for the two considered molecules, in addition to the spectroscopic constants *T*_e_, ω_e_, *B*_e_, *R*_e_, and the values of the dipole moment μ_e_ and the dissociation energy *D*_e_. The determination of vibrational constants *E*_v_, *B*_v_, *D*_v_, and the turning points *R*_min_ and *R*_max_ up to the vibrational level *v* = 100 was possible with the use of the canonical functions schemes. Additionally, the transition and the static dipole moments curves, Einstein coefficients, the spontaneous radiative lifetime, the emission oscillator strength, and the Franck–Condon factors are computed. These calculations showed that the molecule CaCs is a good candidate for Doppler laser cooling with an intermediate state. A “four laser” cooling scheme is presented, along with the values of Doppler limit temperature *T*_D_ = 55.9 μK and the recoil temperature *T*_r_ = 132 nK. These results should provide a good reference for experimental spectroscopic and ultra-cold molecular physics studies.

## Introduction

The discovery of Bose–Einstein condensates^1,2^ and Fermi gases^3^ encouraged researchers to study ultra-cold molecules. More specifically, ultra-polar molecules are of high interest as they exhibit a permanent dipole moment that results from the difference in electronegativity between the atoms, leading to an anisotropic continuing dipole–dipole interaction^4^ in ultra-cold systems. The importance of ultra-cold polar molecules is that they allow the study of modulated chemical reactions^5,6^ and render descriptive quantum computing and quantum reproduction of lattice spin versions^7^ possible. In addition, they help in the study of experimental preparation of few-body quantum effects^8^ and accurate measurements of the variation of the fine structure constant α,^9^ the proton-to electron mass ratio μ ≡ *m*_p_/*m*_e_,^10−14^ and the electron dipole moment.^15,16^ The mixing of an alkali (AK) atom with an alkaline earth (AKE) atom produces an AK–alkaline earth (AK–AKE) molecule with one unpaired electron, which is a polar molecule that has a magnetic dipole moment in the ^2^Σ^+^ ground state. We chose to study the (AK–AKE) molecule because its constituents were cooled precisely, and corresponding quantum degenerate systems were already created.^17−20^ To obtain such a molecule, one can hold two laser-cooled atoms by photoassociation^21^ or Feshbach resonance.^22,23^ Our team recently proposed the (AK–AKE) molecule, CaK, as a potential laser-cooling candidate through the Doppler cooling technique.^24^ Although theoretical studies have been published about some (AK–AKE) molecules such as CsSr,^25^ MgCs,^26^ BaCs,^27^ CaK, CaNa, CaRb,^28^ and CaLi,^29^ there are still data that are missing.

This paper has two aims: the first is to fill the missing gap related to the complete absence of theoretical and experimental data on the molecule CaCs and some higher electronic states of the CaNa molecule. The second aim is to investigate whether either of the two molecules could be cooled down to ultra-cold temperatures using the Doppler laser-cooling technique. Consequently, in this work, the electronic structure of these two molecules has been studied using the ab initio CASSCF/(MRCI + Q) method. The potential energy curves (P.E.C.s), the spectroscopic constants *T*_e_, ω_e_, *B*_e_, *R*_e_, and the static and transition dipole moments (T.D.M.s) have been calculated along with rovibrational constants *E*_v_, *B*_v_, *D*_v_, and the turning points *R*_min_ and *R*_max_. The calculation of the Franck–Condon factor, the radiative lifetime, the vibrational branching ratio, the Doppler limit temperature *T*_D_, the recoil temperature *T*_r_ prove the candidacy of the molecule CaCs only for Doppler laser cooling.

In exploring the practicality of laser cooling of these molecules, we have found that the CaCs molecule is an appropriate candidate for Doppler laser cooling where a laser-cooling scheme is presented.

## Computational Approach

The basic measurements and calculations are performed in the *C*_2*v*_ point-group symmetry with the help of the computational program MOLPRO,^30^ taking advantage of the graphical user interface GABEDIT.^31^ The calculations executed by this program have high accuracy due to the analysis of the electron correlation problem. The calculations for the ground and excited states of the CaCs and CaNa molecules are based on the ab initio methods by using the state averaged complete active space self-consistent field (CASSCF) followed by the multireference configuration interaction (MRCI) method with Davidson correction (+Q). The basis set used for the cesium atom is the quasi-relativistic energy-consistent pseudo-potential ECP46MWB. According to this basis set, 46 electrons are considered frozen with the core of the Cs atom, and as a result, we deal with the cesium atom as a system of nine active electrons only. For the calcium atom of the CaCs molecule, we use the cc-pVQZ quadruple-ζ correlation-consistent polarized basis where all the 20 electrons are considered. Consequently, the CaCs molecule is considered a system of 29 electrons, with three valence electrons. For this molecule, the 13 active orbitals in the *C*_2*v*_ symmetry are 6σ (Ca: 4s, 4p_0_, 3d_0_, 3d ± 2; Cs: 6s, 6p0), 3π (Ca: 4p ± 1, 3d ± 1; Cs: 6p ± 1), 1δ (Ca: 3d ± 2) distributed into the irreducible representation *a*1, *b*1, *b*2, and *a*2 as [6, 3, 3, 1]. The calcium atom of the CaNa molecule has been treated using the quasi-relativistic energy consistent pseudo-potential ECP10MWB, where 10 electrons were frozen within the core, and the remaining 10 electrons are considered active electrons within the considered molecular orbital. The sodium atom Na is treated in all-electron schemes using the cc-pVQZ basis. Consequently, the CaNa molecule is considered a system of 21 electrons, with three valence electrons. For this molecule, the 15 active orbitals in the *C*_2*v*_ symmetry are 8σ (Ca: 4s, 4p_0_, 3d_0_, 3d ± 2, 5s; Na: 3s, 3p_0_, 4s), 3π (Ca: 4p ± 1, 3d ± 1; Na: 3p ± 1), 1δ (Ca: 3d ± 2) distributed into the irreducible representation *a*1, *b*1, *b*2, and *a*2 as [8, 3, 3, 1]. We used this combination of basis sets for the two molecules due to the successful results obtained by other groups and previously published papers^32−35^ that used a similar combination of basis sets for AK–AKE compounds. Additionally, and for more accuracy and comparison, we used the perturbation theory (Rayleigh–Schrödinger perturbation theory) RSPT2-rs2 to calculate the spectroscopic constants for some electronic states for CaCs and CaNa molecules. The RSPT2-rs2 calculations have been done using the same basis sets as the MRCI/CASSCF method, considering three valence electrons for the two molecules. By using the same methods and computing packages, we have also calculated the lowest-lying molecular curves of the CaNa molecule using Aug-cc-pVQZ for the Na atom.

## Results and Discussion

### Potential Energy Curves

In this work, we draw the P.E.C.s for 25 doublet and quartet low-energy electronic states for the CaCs molecule and 32 doublet and quartet electronic states for the CaNa molecule as a function of the internuclear distance shown in Figures 1–8.

**Figure 1:**
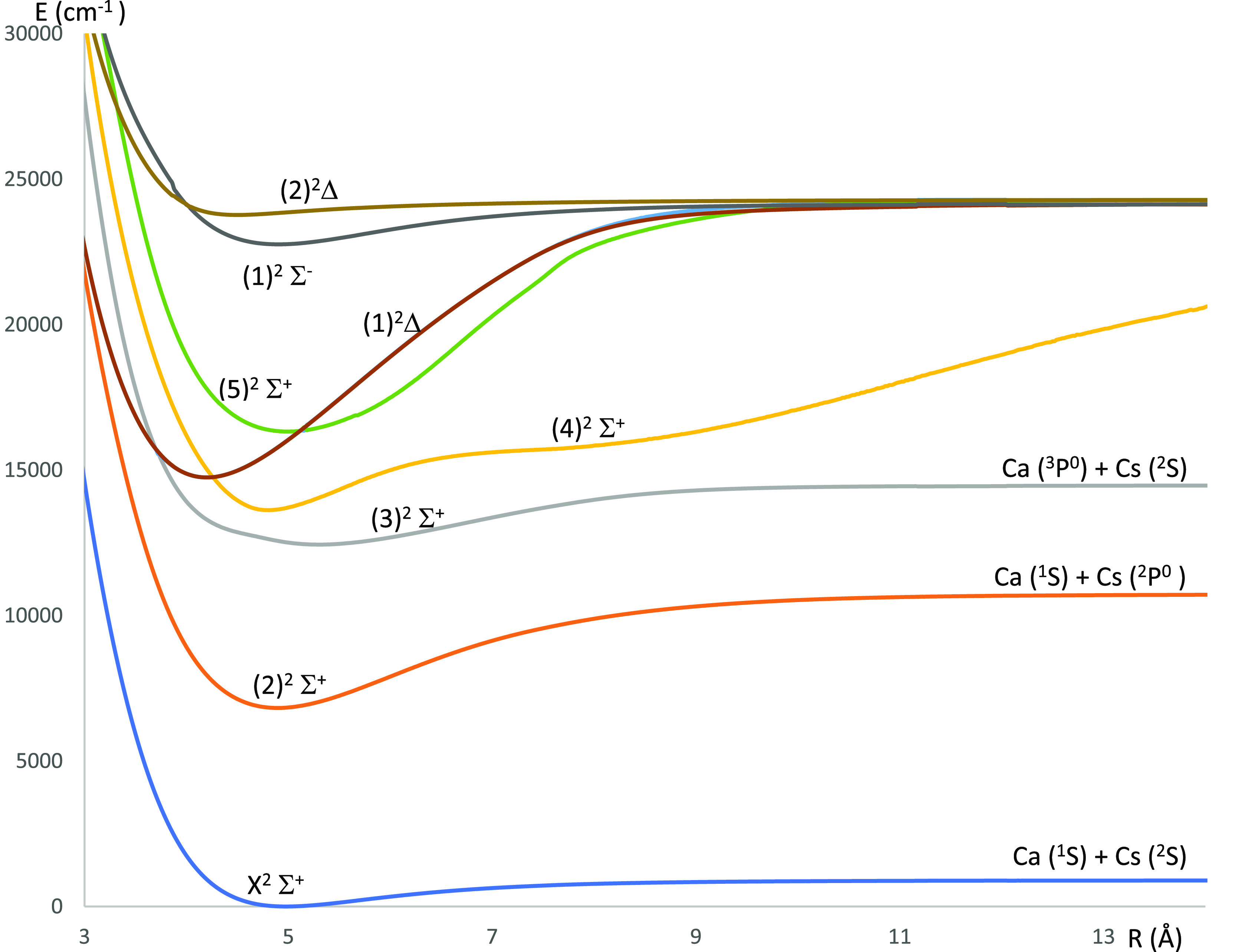
Potential energy curves of the lowest ^2^Σ^+^ and ^2^Δ electronic states of the CaCs molecule using the CASSCF/MRCI method with three valence electrons.

**Figure 2:**
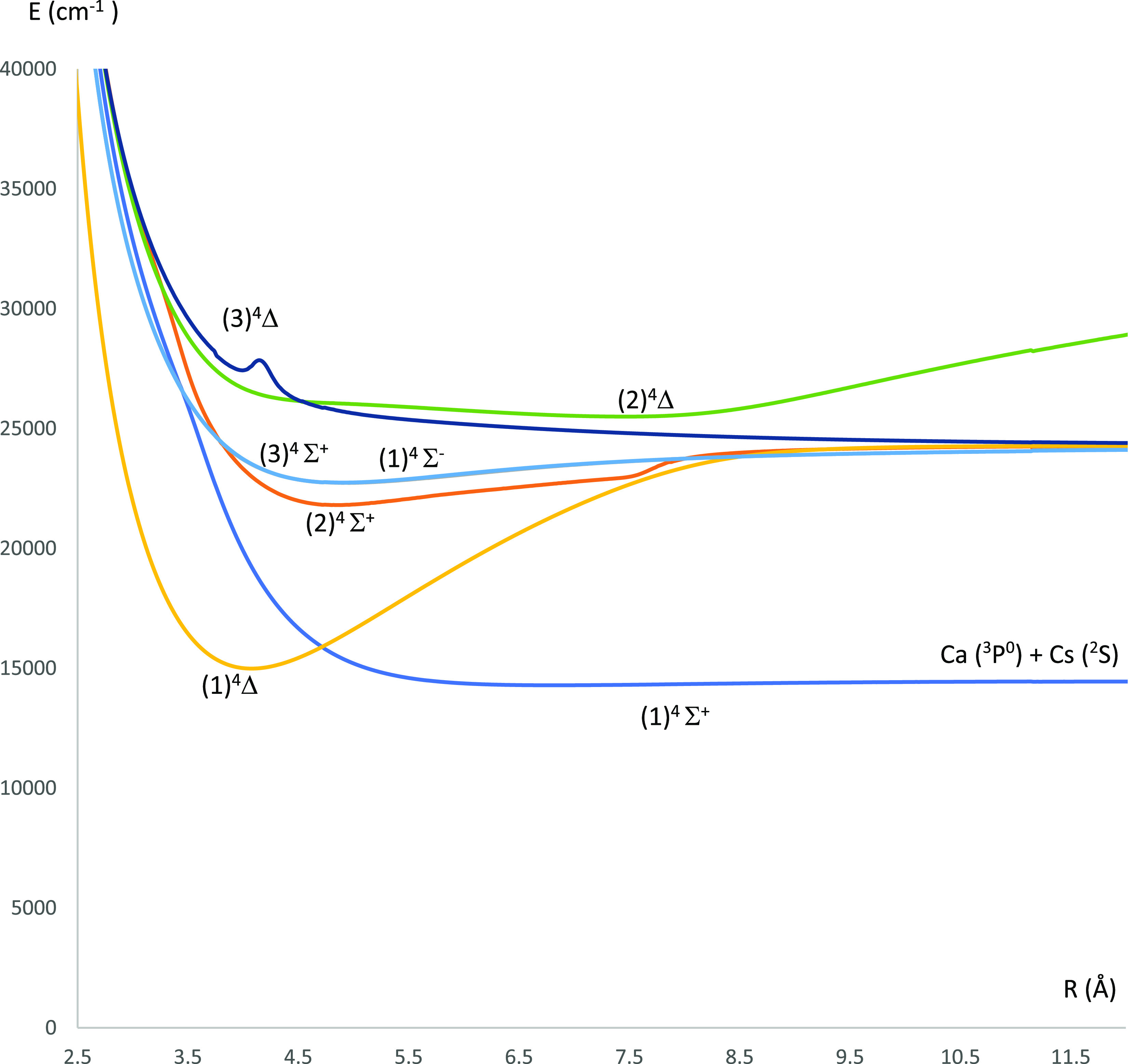
Potential energy curves of the lowest ^4^Σ^(±)^ and ^4^Δ electronic states of the CaCs molecule using the CASSCF/MRCI method with three valence electrons.

**Figure 3:**
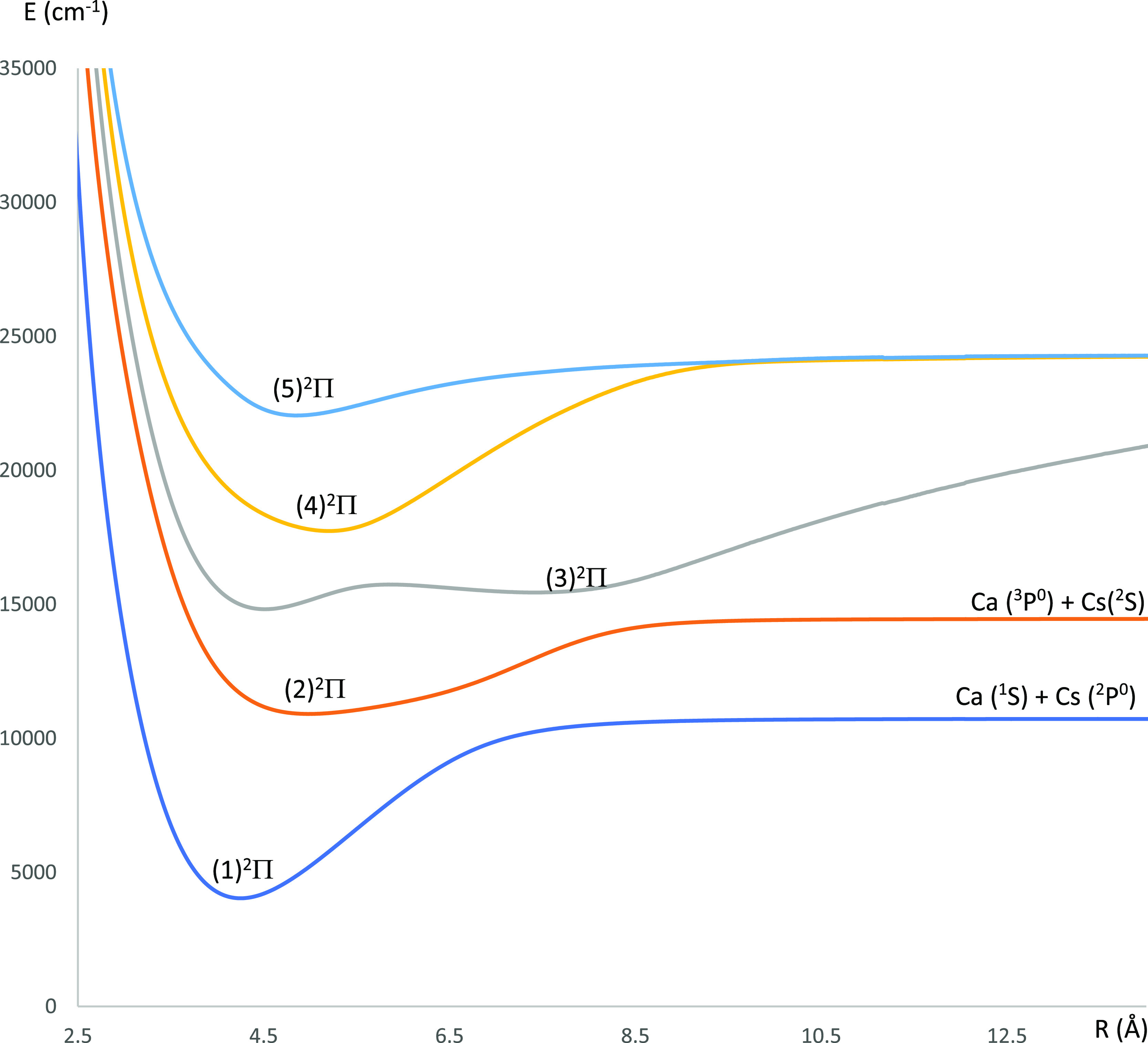
Potential energy curves of the lowest ^2^Π electronic states of the CaCs molecule using the CASSCF/MRCI method with three valence electrons.

**Figure 4:**
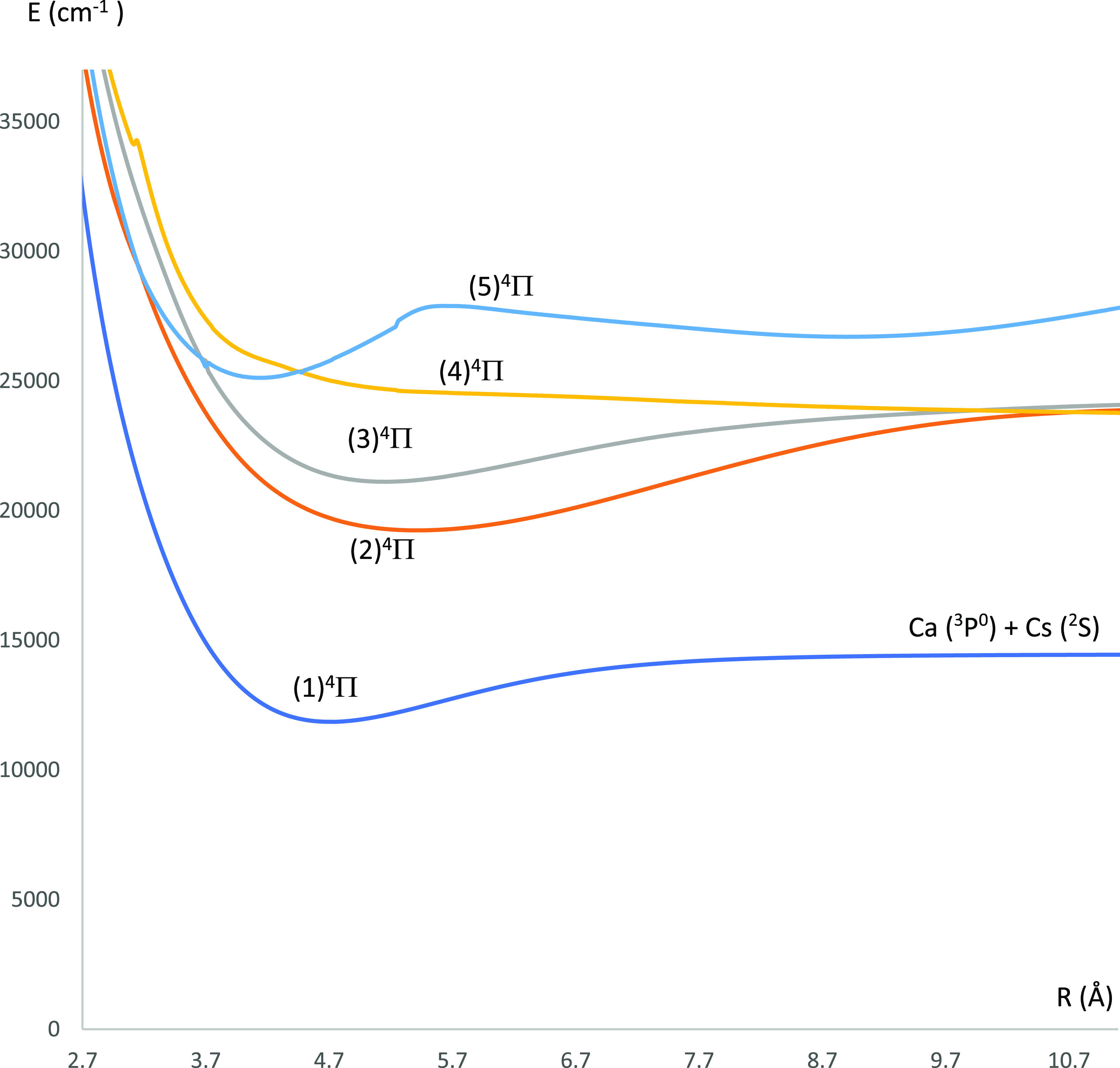
Potential energy curves of the lowest ^4^Π electronic states of the CaCs molecule using the CASSCF/MRCI method with three valence electrons.

**Figure 5:**
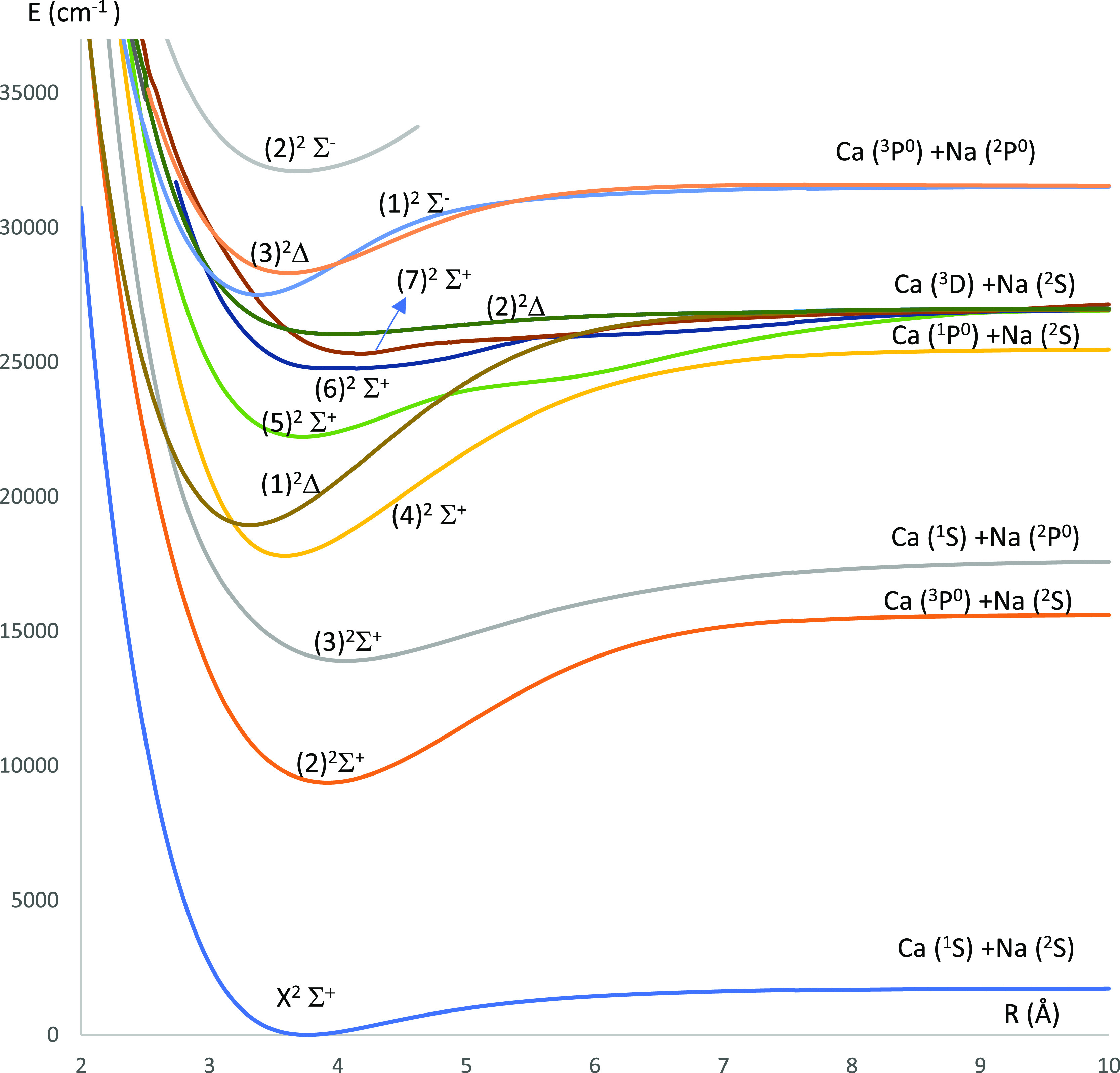
Potential energy curves of the lowest ^2^Σ^+^ and ^2^Δ electronic states of the CaNa molecule using the CASSCF/MRCI method with three valence electrons.

**Figure 6:**
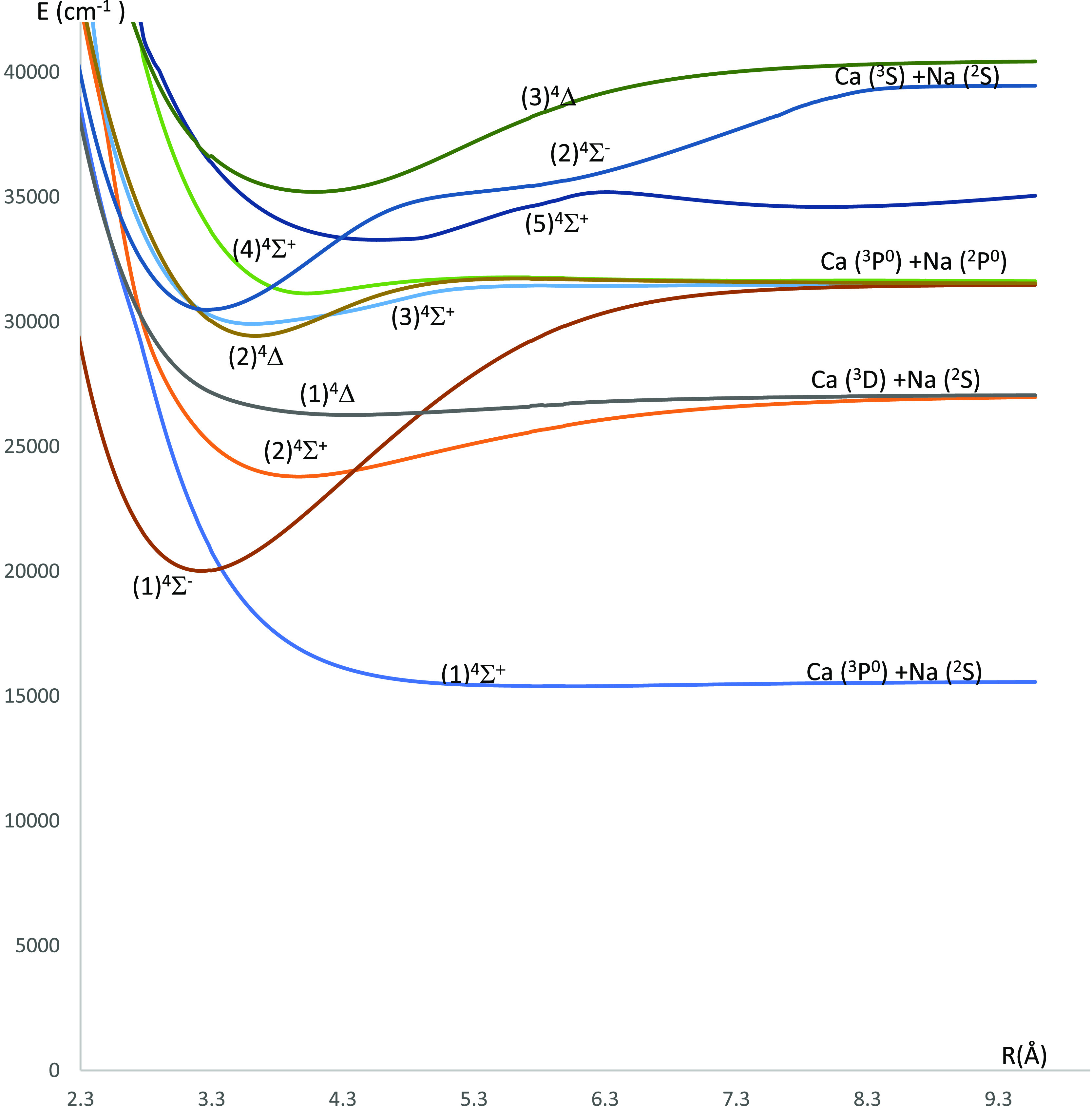
P.E.C.s of the lowest ^4^Σ^(±)^ and ^4^Δ electronic states of the CaNa molecule using the CASSCF/MRCI method with three valence electrons.

**Figure 7:**
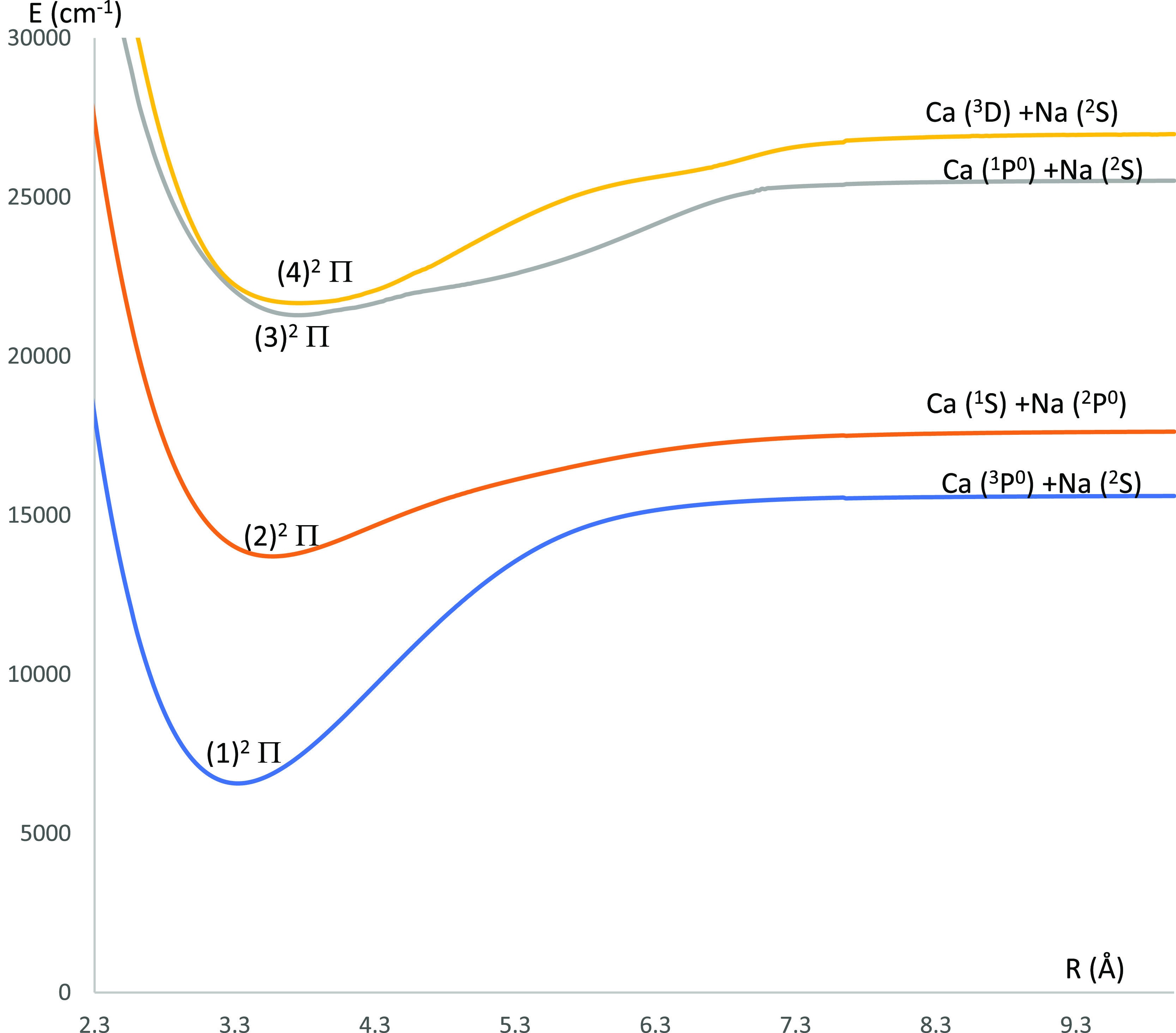
P.E.C.s of the lowest ^2^Π electronic states of the CaNa molecule using the CASSCF/MRCI method with three valence electrons.

**Figure 8:**
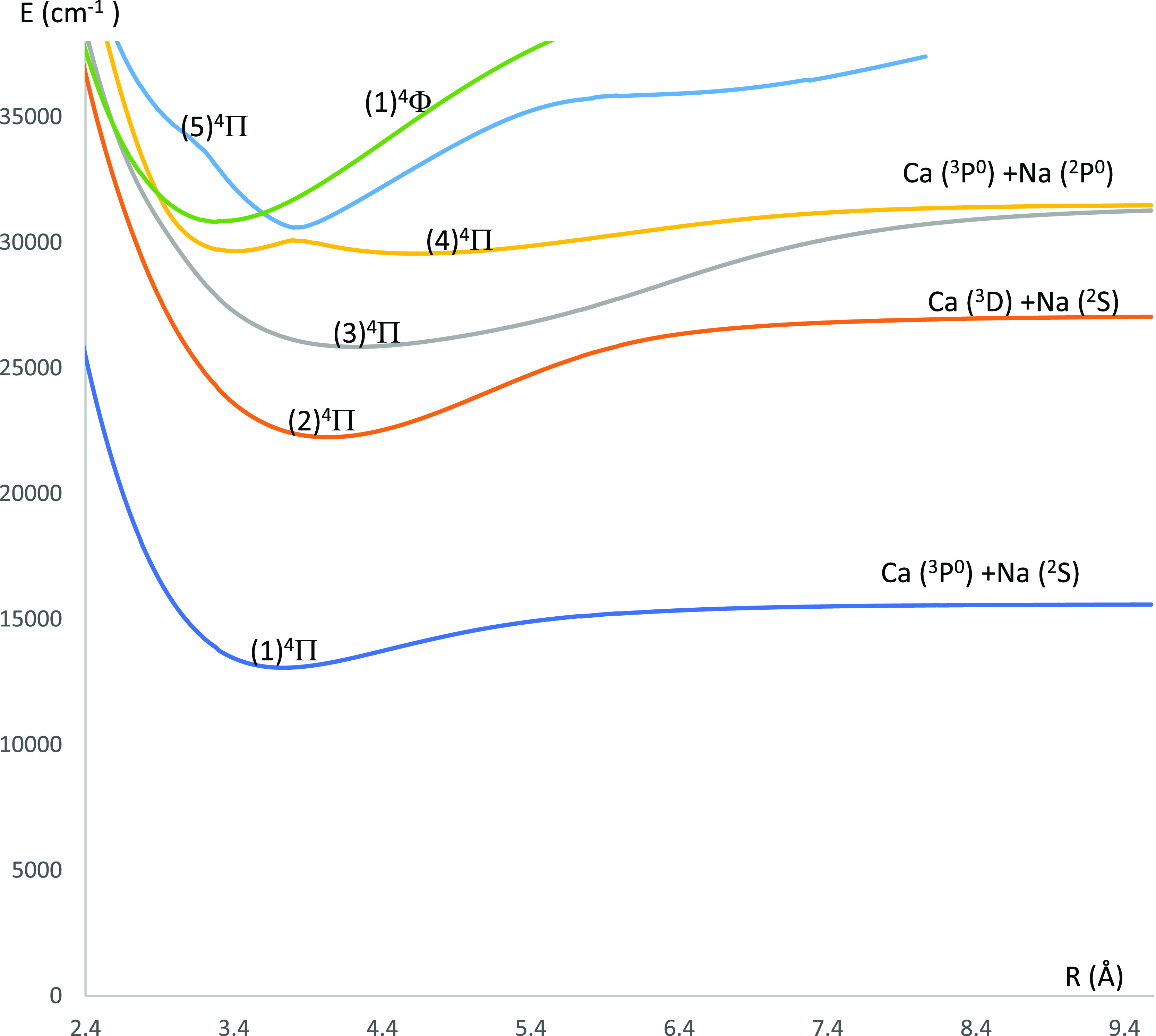
P.E.C.s of the lowest ^4^Π electronic states of the CaNa molecule using the CASSCF/MRCI method with three valence electrons.

The kind of forces holding the atoms specify and control the shape of the obtained curve. Shallow wells are obtained for some electronic states when the repulsive forces overcome the attractive ones within the considered range of internuclear distance.

The cesium and the sodium atoms have an unpaired electron; therefore, they will remain in the doublet state, while the calcium atom Ca exists either in the singlet or in the triplet state. Since the combination of a doublet alkali metal atom with a singlet lowest state of alkaline earth metal results in a doublet state of the molecule, X^2^Σ^+^ is the ground state of the two molecules CaCs and CaNa. Now, the combination between the doublet state of Cs and Na atoms with the triplet state of a Ca atom results in the quartet states ^4^Σ^+^ of the two molecules.

The P.E.C.s of the doublet electronic states of the CaCs molecule are shown in Figure 1 (^2^Σ^+^ and ^2^Δ electronic states) and Figure 3 (^2^Π electronic states). The P.E.C.s of the quartet states of the CaCs molecule are shown in Figure 2 (^4^Σ^(±)^ and ^4^Δ electronic states) and Figure 4 (^4^Π electronic states). The P.E.C.s of the doublet electronic states of the CaNa molecule are shown in Figure 5 (^2^Σ^+^ and ^2^Δ electronic states) and Figure 7 (^2^Π electronic states). The P.E.C.s of the quartet states of the CaNa molecule are shown in Figure 6 (^4^Σ^(±)^ and ^4^Δ electronic states) and Figure 8 (^4^Π electronic states). The P.E.C.s for the molecule CaNa obtained with the Aug-cc-pVQZ basis sets for the Na atom are displayed in Figure S1 in the Supporting Information.

Table 1 presents the lowest dissociation limits of the calculated low-lying electronic states of the two molecules CaCs and CaNa compared to the combination of atomic orbital values obtained from the National Institute of Standards and Technology website (NIST).^36^ As a result of the fluctuations and oscillations in the P.E.C at the long-range of the internuclear distance *R*, the dissociation limits of some of the higher excited molecular states are not achieved, and consequently, these higher molecular states are not considered. A good clarification of these fluctuations in the P.E.C is the Born–Oppenheimer approximation breakdown. The comparison of the dissociation limits of the investigated P.E.C with those obtained with the NIST database agrees well with a relative difference of 14% for the first dissociation limit and 10.9% for the second of the CaCs molecule. For the molecule CaNa, a good agreement is also attained between the values of the dissociation limits obtained with NIST and our calculated values with a relative difference of 0.38 < Δ*D*_e_/*D*_e_ < 8.35, except for the fourth dissociation limit where the relative difference is 19.64%. The corresponding values of the dissociation energies *D*_e_ are presented in Tables 2 and 3.

**Table 1:** Lowest Dissociation Limits of CaCs and CaNa Molecules

dissociation of atomic levels Ca + Cs	dissociation energy limit of CaCs levels (cm^–1^)	molecular states of CaCs	total dissociation energy limit of Ca + Cs atoms (cm^–1^)	relative error (%)
Ca (3p^6^4s^2^, ^1^S) + Cs (5p^6^6s, ^2^S)	0^a^	X^2^Σ^+^	0^b^	0.0
Ca (3p^6^4s^2^, ^1^S) + Cs (5p^6^6p, ^2^P^0^)	9832^a^	(2)^2^Σ^+^, (1)^2^Π	11 455^b^	14
Ca (3p^6^4s4p, ^3^P^0^) + Cs (5p^6^6s, ^2^S)	13 561^a^	(2)^2^Π, (3)^2^Σ^+^, (1)^4^Π, (1)^4^Σ^+^	15 228^b^	10.9

configuration of Ca + Na	dissociation energy limit of CaNa levels (cm^–1^)	molecular states of CaNa	total dissociation energy limit of Ca + Na atoms (cm^–1^)	relative error (%)
Ca (3p^6^4s^2^, ^1^S) + Na (2p^6^3s, ^2^S)	0.00^a^	X^2^Σ^+^	0.00^b^	0.00
Ca (3p^6^4s4p, ^3^P^0^) + Na (2p^6^3s, ^2^S)	13 891.6^a^	(2)^2^Σ^+^, (1)^2^Π, (1)^4^Σ^+^, (1)^4^Π	15 157.9^b^	8.35
Ca (3p^6^4s^2^, ^1^S) + Na (2p^6^3p, ^2^P^0^)	15 908.2^a^	(3)^2^Σ^+^, (2)^2^Π	16 956.1^b^	6.18
Ca (3p^6^4s4p, ^1^P^0^) + Na (2p^6^3s, 2S)	23 744.1^a^	(4)^2^Σ^+^, (3)^2^Π	23 652.3^b^	0.38
Ca (3p^6^3d4s, ^3^D) + Na (2p^6^3s, ^2^S)	25 307.4^a^	(5)^2^Σ^+^, (6)^2^Σ^+^, (4)^2^Π, (1)^2^Δ, (2)^4^Σ^+^, (7)^2^Σ^+^, (2)^4^Π, (2)^2^Δ, (1)^4^Δ	20 335.3^b^	19.64
Ca (3p^6^4s4p, ^3^P^0^) + Na (2p^6^3p, ^2^P^0^)	29 922.8^a^	(3)^4^Σ^+^, (3)^4^Π, (1)^2^Σ^–^, (3)^2^Δ, (4)^4^Σ^+^, (4)^4^Π, (1)^4^Σ^–^, (2)^4^Δ	32 114.0^b^	6.82
Ca (3p^6^4s6s, ^3^S) + Na (2p^6^3s, ^2^S)	37 737.3^a^	(2)^4^Σ^–^	40 474.2^b^	6.76

a Present work.

b Experimental data from the NIST atomic spectra database.

**Table 2:** Spectroscopic Parameters for the X^2^Σ^+^ and 13 Excited States of the CaCs Molecule

states (^2*s*+1^Λ)	method	*T*_e_ (cm^–1^)	Δ*T*_e_/*T*_e_ %	*R*_e_ (Å)	Δ*R*_e_/*R*_e_ %	ω_e_ (cm^–1^)	Δω_e_/ω_e_ %	*B*_e_ (cm^–1^)	Δ*B*_e_/*B*_e_ %	*D*_e_ (cm^–1^)	|μ_e_| (au)
X^2^Σ^+^	MRCI	0		4.978	+3.6	41.44	–23.9	0.0221	–6.8	894.192	3.762
	perturbation	0		5.156		31.52		0.0206			
(1)^2^Π	MRCI	4034.09	+8.2	4.254	–0.3	84.00	+1.9	0.0302	+0.7	6691.755	11.222
	perturbation	4366.76		4.240		85.62		0.0304			
(2)^2^Σ^+^	MRCI	6824.70	+6.9	4.899	+0.08	59.37	+3.9	0.0228	0.0	3890.082	4.189
	perturbation	7295.34		4.903		61.68		0.0228			
(2)^2^Π	MRCI	10 912.00	+1.8	4.990	+2.5	41.38	–7.7	0.0220	–5.0	3545.286	2.779
	perturbation	11 110.40		5.113		38.17		0.0209			
(1)^4^Π	MRCI	11 855.64	+1.0	4.711	+1.2	60.39	–6.0	0.0248	–2.8	2600.873	9.495
	perturbation	11 970.20		4.767		56.79		0.0241			
(4)^2^Σ^+^	MRCI	13 624.61	+13.4	4.802	–4.3	78.07	+26.9	0.0237	+9.3	6994.970	0.482
	perturbation	15 458.38		4.596		99.04		0.0259			
(1)^2^Δ	MRCI	14 747.09	+5.7	4.200	–0.4	81.19	+2.7	0.0310	+1.0	9382.437	11.967
	perturbation	15 586.57		4.183		83.42		0.0313			
(3)^2^Π	MRCI	14 819.56	+7.5	4.516	+0.4	67.55	–14	0.0268	–3.0	6104.507	6.275
	perturbation	15 928.58		4.589		58.08		0.0260			
(1)^4^Δ	MRCI	14 987.06	+5.36	4.077	+0.2	82.63	+12.5	0.0329	–0.3	9174.208	10.343
	perturbation	15 790.82		4.086		92.94		0.0328			
(4)^2^Π	MRCI	17 737.17	+2.0	5.204	–2.8	57.06	+13.7	0.0202	+6.0	6503.924	1.307
	perturbation	18 092.90		5.056		64.91		0.0214			
(2)^4^Π	MRCI	19 238.21	+0.08	5.412	–0.6	40.12	+14	0.0187	+1.1	4886.337	2.304
	perturbation	19 254.39		5.379		45.75		0.0189			
(3)^4^Π	MRCI	21 112.83	+1.2	5.154	+3.5	48.97	–1.5	0.0206	–5.8	3130.329	5.347
	perturbation	21 370.67		5.334		48.23		0.0194			
(5)^2^Π	MRCI	22 048.66	+0.8	4.857	+5.9	55.01	–23.3	0.0232	–10.8	2232.292	1.929
	perturbation	22 226.51		5.142		42.20		0.0207			
(1)^4^Σ^–^	MRCI	22 747.93	+2.0	4.911	+10.7	36.86	–33.0	0.0227	–18.5	1540.725	7.521
	perturbation	23 197.12		5.437		24.69		0.0185			

**Table 3:** Spectroscopic Parameters for the X^2^Σ^+^ and 26 Excited States of the CaNa Molecule (Na Atom: cc-pVQZ and aug-cc-pVQZ)

states Λ^2*s*+1^	method [reference]	*T*_e_ (cm^–1^)	Δ*T*_e_/*T*_e_ %	*R*_e_ (Å)	Δ*R*_e_/*R*_e_ %	ω_e_ (cm^–1^)	Δω_e_/ω_e_ %	*B*_e_ (cm^–1^)	Δ*B*_e_ /*B*_e_ %	*D*_e_ (cm^–1^)	Δ*D*_e_/*D*_e_ %	|μ_e_| (au)	Δμ_e_/μ_e_ %
X^2^Σ^+^	CASSCF/MRCI (Na: cc-pVQZ basis)[this work]	0.00		3.759		101.3		0.0817		1721		1.38	
	CASSCF/MRCI (Na: Aug-cc-pVQZ) [this work]	0.00		3.749	0.3	103.4	2.1	0.0821	0.5				
	perturbation [this work]			3.851	2.4	91.8	9.4	0.0778	4.8				
	CASSCF/MRCI^36^			3.670	2.3	103.0	1.6	0.0830		1792	3.9	1.18	14.5
	CCSD(T)^27^			3.720	1.0	97.0	4.2		1.5	1453	15.5	1.01	26.8
	CASSCF/MRCI^37^			3.665	2.5	103.0	1.6			1802	4.4	1.17	15.2
	CASPT2^37^			3.666	2.4	102.6	1.2			1752	1.7	1.09	21.0
	CCSD^37^			3.720	1.0	88.5	12.6			1264	26.5		
(1)^2^Π	CASSCF/MRCI (Na: cc-pVQZ basis)[this work]	6572.57	2.6	3.323	0.00	164.4	0.7	0.1045	0.00	9034	12.0	2.85	20.0
	CASSCF/MRCI (Na: Aug-cc-pVQZ) [this work]	6401.98	3.6	3.323	0.69	163.3	2.6	0.1045	1.3	10 275	11.4	2.28	18.9
	perturbation [this work]	6807.73	3.6	3.346	2.97	160.1	4.5	0.1031		10 199	3.4	2.31	
	CASSCF/MRCI^37^	6825	4.0	3.224	2.94	172.2	4.5			9360			
	CASPT2^37^	6851	8.8	3.225	2.25	172.2	6.1						
	CCSD^37^	7211		3.248		175.1							
(2)^2^Σ^+^	CASSCF/MRCI (Na: cc-pVQZ basis) [this work]	9374.00	0.3	3.919	0.2	119.8	0.4	0.0751	0.4	6226	8.6	0.99	45.4
	CASSCF/MRCI (Na: Aug-cc-pVQZ) [this work]	9344.73	1.1	3.927	0.7	120.3	1.2	0.0748	1.5	6815	7.3	0.54	1.0
	perturbation [this work]	9472.78	8.6	3.948	3.1	118.4	3.8	0.0740		6721	2.4	0.98	
	CASSCF/MRCI^37^	10 261	9.0	3.795	3.0	124.6	4.0			6076			
	CASPT2^37^	10 305	10.4	3.800	5.6	124.9	7.9						
	CCSD^37^	10 472		3.696		130.1							
(1)^4^Π	CASSCF/MRCI [this work]	13 065.21	1.5	3.734	1.34	109.7	6.6	0.0828	2.6	2515	11.1	3.08	19.5
	perturbation [this work]	12 872.69	8.2	3.784	2.75	102.4	2.9	0.08060		2832	10.1	2.48	19.8
	CASSCF/MRCI^37^	14 238	8.1	3.631	2.78	113.0	3.0			2800	0.9	2.47	
	CASPT2^37^	14 220	6.6	3.630	2.46	113.1	0.7			2540			
	CCSD^37^	13 998		3.64		110.5							
(2)^2^Π	CASSCF/MRCI (Na: cc-pVQZ basis) [this work]	13 708.69	0.7	3.570	0.14	124.2	2.1	0.0905	0.4	3920	21.6	3.25	29.5
	CASSCF/MRCI (Na: Aug-cc-pVQZ) [this work]	13 611.21	2.7	3.565	3.00	126.8	9.5	0.0909	5.7	5003	17.8	4.61	16.9
	perturbation [this work]	14 080.64	0.5	3.677	4.98	112.4	11.6	0.0853		4773	14.2	3.91	
	CASSCF/MRCI^37^	13 786	1.8	3.392	4.92	140.6	11.4			4573			
	CASPT2^37^	13 966	0.1	3.394	7.31	140.3	15.1						
	CCSD^37^	13 688		3.309		146.4							
(3)^2^Σ^+^	CASSCF/MRCI (Na: cc-pVQZ basis) [this work]	13 894.96	0.3	4.065	0.54	100.5	2.7	0.0699	0.8	3682	11.9	0.52	30.8
	CASSCF/MRCI (Na: Aug-cc-pVQZ) [this work]	13 849.72	1.9	4.043	2.36	97.8	16.7	0.0705	4.7	4180	10.0	0.36	3.9
	perturbation [this work]	14 162.47	4.7	4.161	4.32	117.3	8.0	0.0666		4094	7.3	0.50	
	CASSCF/MRCI^37^	14 585	4.9	3.889	4.37	92.4	4.9			3413			
	CASPT2^37^	14 622	6.2	3.887	6.10	95.1	22.2						
	CCSD^37^	14 814		3.817		78.1							
(1)^4^Σ^+^	CASSCF/MRCI [this work]	15 390.75	4.4	6.041	7.2	22.9	12.2	0.0316	14.2	176	25.7	1.67	3.0
	perturbation [this work]	14 708.90	8.2	6.474	4.9	25.7	4.5	0.0271		237	29.8	1.62	4.8
	CASSCF/MRCI^36^	16 775	8.2	5.740	5.0	24.0	3.3			251	12.0	1.59	10.1
	CASSCF/MRCI^37^	16 775	5.5	5.735	3.3	23.7	4.8			200	14.5	1.50	
	CASPT2^37^	16 288		5.838	2.6	21.8	7.4			206			
	CCSD^37^			5.882		21.2							
(4)^2^Σ^+^	CASSCF/MRCI[this work]	17 800.28		3.588		156.5		0.0897		7665		0.03	
(1)^4^Σ^–^	CASSCF/MRCI[this work]	20 017.54	0.3	3.221	0.6	162.3	2.5	0.1111	1.1	11 463		1.27	
	perturbation [this work]	20 086.76		3.241		166.4		0.1099					
(3)^2^Π	CASSCF/MRCI (Na: cc-pVQZ basis) [this work]	21 289.08	2.5	3.754	0.6	114.4	5.1	0.0819	1.2	4227		4.85	
	CASSCF/MRCI (Na: Aug-cc-pVQZ) [this work]	20 755.62		3.775		108.5		0.0809					
(4)^2^Π	CASSCF/MRCI (Na: cc-pVQZ basis) [this work]	21 670.05	0.5	3.761	0.6	82.3	5.1	0.0816	0.9	5311		2.65	
	CASSCF/MRCI (Na: Aug-cc-pVQZ) [this work]	21 561.58		3.737		86.5		0.0823					
(5)^2^Σ^+^	CASSCF/MRCI[this work]	22 223.91		3.726		117.1		0.0832		4692		4.04	
(2)^4^Π	CASSCF/MRCI[this work]	22 242.86	0.1	4.037	1.3	107.0	1.9	0.0709	2.7	4792		2.36	
	perturbation [this work]	22 266.68		4.089		105.0		0.0690					
(2)^4^Σ^+^	CASSCF/MRCI [this work]	23 797.06	0.2	3.964	3.9	92.4	17.0	0.0734	7.3	3183		4.17	
	perturbation [this work]	23 847.03		4.119		76.7		0.0680					
(6)^2^Σ^+^	CASSCF/MRCI [this work]	24 767.42		3.926		39.5		0.0744		2169		12.27	
(3)^4^Π	CASSCF/MRCI [this work]	25 841.54	0.3	4.222	3.0	74.8	6.9	0.0648	5.8	5428		1.36	
	perturbation [this work]	25 771.95		4.348		69.6		0.06104					
(2)^2^Δ	CASSCF/MRCI (Na: cc-pVQZ basis) [this work]	26 034.51	1.6	3.978	3.4	64.0	28.3	0.0730	7.0	915		3.61	
	CASSCF/MRCI (Na: Aug-cc-pVQZ) [this work]	25 605.11		3.842		82.1		0.0781					
(1)^4^Δ	CASSCF/MRCI[this work]	26 267.05		4.355		45.3		0.0608		789		2.24	
(3)^2^Δ	CASSCF/MRCI[this work]	28 307.45		3.616		121.3		0.0883		3251		0.77	
(2)^4^Δ	CASSCF/MRCI[this work]	29 435.36	0.5	3.634	0.2	142.0	6.5	0.0874	0.3	2103		1.83	
	perturbation [this work]	29 595.75		3.628		132.8		0.0877					
(4)^4^Π 2nd min	CASSCF/MRCI[this work]	29 554.57		4.626		56.3		0.0540		1921		4.43	
(4)^4^Π 1st min	CASSCF/MRCI[this work]	29 652.33		3.412		148.0		0.0991		1761		0.55	
(3)^4^Σ^+^	CASSCF/MRCI[this work]	29 914.75	1.2	3.610	3.5	105.0	6.3	0.0886	6.8	1584		1.74	
	perturbation [this work]	30 268.02		3.738		98.4		0.0826					
(1)^4^Φ	CASSCF/MRCI [this work]	30 830.53		3.267		171.5		0.1073				0.92	
(4)^4^Σ^+^	CASSCF/MRCI[this work]	31 135.44	4.6	4.027	3.5	114.1	31.0	0.0712	6.9	492		2.97	
	perturbation [this work]	32 584.45		4.170		78.7		0.0663					
(2)^2^Σ^–^	CASSCF/MRCI [this work]	32 086.31	0.7	3.687	0.7	106.0	15.5	0.0850	1.5			1.97	
	perturbation [this work]	32 299.26		3.713		122.4		0.0837					
(5)^4^Σ^+^ 1st min	CASSCF/MRCI[this work]	33 283.47		4.569		65.0		0.0553				7.44	
(5)^4^Σ^+^ 2nd min	CASSCF/MRCI[this work]	34 600.65		8.002		88.2		0.0168				11.67	
(3)^4^Δ	CASSCF/MRCI[this work]	35 205.66		4.082		89.0		0.0692		5226		5.17	

In Figures 1–8, one can notice some shallow potential energy wells, which are the evidence of the dominant Coulomb repulsive forces over the attractive ones. In these figures, for the two considered molecules, the avoided crossings that occur between the adiabatic states are the results of an interplay between the ionic state and all other states. Crossings are also generated due to the strong repulsive behavior of the electronic states. The positions of crossing and avoided crossing are provided in Table S1 in the Supporting Information with their corresponding energy gaps Δ*E*. As a result, the appearance of a barrier potential and multiple wells are due to the avoided crossing behavior corresponding to a crossing in the diabatic picture.

### Spectroscopic Parameters

The spectroscopic constants ω_e_, *R*_e_, *B*_e_, *T*_e_, and *D*_e_ of the two molecules CaCs and CaNa electronic states have been calculated by fitting the P.E.C values around the minimum of the internuclear distance *R*_e_ to a polynomial in terms of *R*. These constants are obtained by using CASSCF/MRCI and perturbation RSPT2-rs2 methods for 14 electronic states of the molecule CaCs and 27 electronic states for the molecule CaNa. They are presented in Tables 2 and 3, respectively. For the molecule CaCs, no comparison can be made between our calculated values and those of the literature since they are presented here for the first time. However, there is an important agreement between the results we obtained using CASSCF/MRCI and RSPT2-rs2 methods.

Our calculated equilibrium bond distance *R*_e_ and harmonic frequency ω_e_ for the ground state X^2^Σ^+^ of the CaNa molecule overlap well with those in the three references,^28,37,38^ with relative differences of 1% ≤ Δ*R*_e_/*R*_e_ ≤ 2.5% and 1.2% ≤ Δω_e_/ω_e_ ≤ 4.2%, respectively, except for a larger relative difference Δω_e_/ω_e_ = 10.5%^38^ calculated by using the CCSD method. Our calculated value of *B*_e_ is very close to that calculated by Gopakumar et al.^28^ with a relative difference Δ*B*_e_/*B*_e_ = 1.5%.

For the excited states, our values of *T*_e_ compare well with those in the literature for seven excited electronic states, where the relative differences vary as 0.1%(2)^2^Π^38^ < Δ*T*_e_/*T*_e_ < 10.4%(2)^2^∑^+^.^38^ Similarly, the internuclear distance *R*_e_ also shows a very good agreement when compared with published data, with a relative difference of 2.25%^38^ ≤ Δ*R*_e_/*R*_e_ ≤ 7.31%.^38^ The comparison of our results with the values of ω_e_ obtained by different techniques in the literature shows a good agreement with the relative difference of 7%(1)^4^Π^38^ ≤ Δω_e_/ω_e_ ≤ 1.6%(1)^2^Π.^38^ The comparison of our calculated values of ω_e_ with those obtained by using the CCSD method^38^ shows a relative difference of 22.2%. There is no comparison for the other investigated states since they are calculated here first.

The comparison of our calculated values of the spectroscopic constants by using the Aug-cc-pVQZ basis sets for Na atom in Table 3 with those calculated by using the cc-pVQZ basis set for the same atom shows an excellent agreement with the average relative differences of the ground and the studied excited states Δ*T*_e_/*T*_e_ = 0.97%, Δ*R*_e_/*R*_e_ = 0.29%, Δω_e_/ωe = 1.32%, and Δω_e_/ω_e_ = 0.52%. Given these values, we estimate that the use of the diffuse Gaussian basis functions (aug-) for the Na atom has no real effect on the investigated data of the molecule CaNa.

The absence of spectroscopic constants of some electronic states is referred to the presence of avoided crossing near the minima of these states. As a verification of the accuracy of our results given in Tables 2 and 3, the trend of the spectroscopic constants is presented in Table 4, where *T*_e_, ω_e_, and *B*_e_ decrease for each electronic state with the decrease of the electronegativity and *R*_e_ increases as we go from CaNa to CaCs.

**Table 4:**
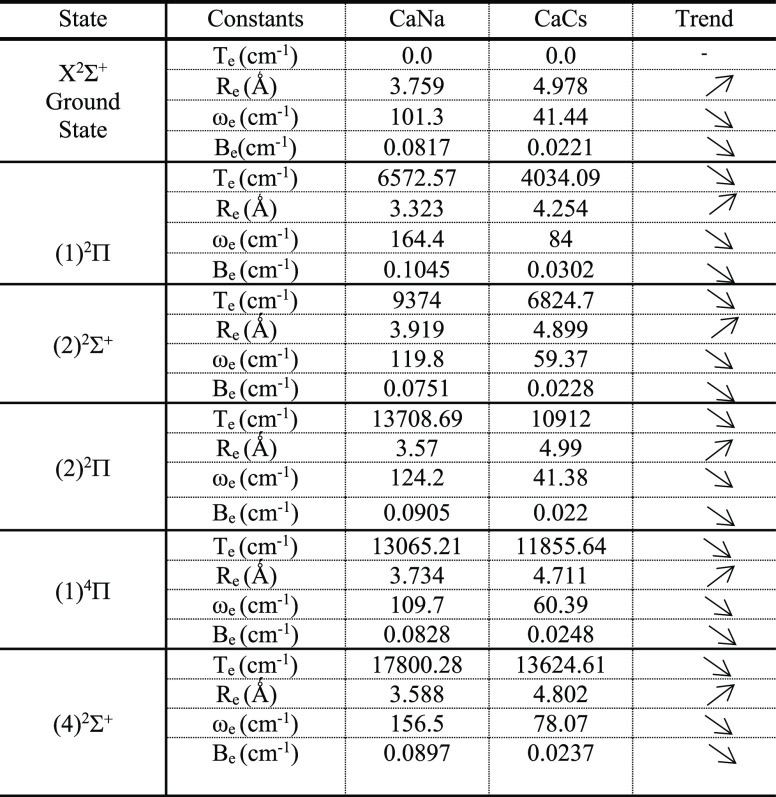
Study of the Trend of the Spectroscopic Constants of the Different Electronic States of the Molecules CaNa and CaCs

### Permanent Dipole Moment

The permanent dipole moment of a diatomic molecule is an important parameter since it clarifies the type of bonding (ionic/covalent) and the polarity of a given molecular interaction. As stated in the Introduction, the importance of polar ultra-cold molecules lies in using long-life interactions among their permanent dipole moments in specific applications. We have estimated the dipole moment curves (D.M.C.s), representing the molecular permanent dipole moment variation with the internuclear distance *R*, for the 25 lowest doublet and quartet electronic states of CaCs and the 32 lowest doublet and quartet electronic states of the CaNa molecule. These curves are plotted in the Supporting Information, in Figures S2–S9. The electron density distribution controls the values of the dipole moments. The geometry of the investigated systems is such that the calcium atom is chosen to be at the origin for both CaNa and CaCs molecules. Consequently, a charge transfer from Ca to Cs and from Ca to Na leads to negative dipole moment values when the charge density is closer to the Cs and Na atoms. This polarity is indicated as Ca^δ+^Cs^δ−^ and Ca^δ+^Na^δ−^ for CaCs and CaNa molecules, respectively.

The permanent dipole moment (PDM) curves for the ground state X^2^Σ^+^ of the two molecules CaCs and CaNa are positive with maximum values |μ_e_| = 1.78 au at *R* = 4.14 Å and |μ_e_| = 0.691 au at *R* = 2.92 Å, respectively. The curves reach zero at a large distance (*R* = 10 Å), indicating the molecule’s breaking into a neutral fragment. The absolute values of μ_e_ were calculated for both molecules’ ground and excited states and are tabulated in Tables 2 and 3. It should be noted here that the abrupt gradient change of the PDM curves is due to the occurrence of an avoided crossing between the P.E.C.s of two states of the same symmetry. The positions of the avoided crossings concur with those of the D.M.C. polarity shifts.

The comparison of our calculated PDM(μ_e_) values for the ground state (X)^2^∑^+^ of the molecule CaNa shows an almost acceptable agreement with the values present in the literature with a relative error 14.5%^37^ ≤ Δμ_e_/μ_e_ ≤ 21%.^38^ The first excited quartet state (1)^4^∑^–^ shows a good agreement with a relative error 3%^37^ ≤ Δμ_e_/μ_e_ ≤ 10.1%.^38^ The comparison of our calculated values of (μ_e_) for the excited states (1)^2^Π, (2)^2^∑^+^, (1)^4^Π, (2)^2^Π, (3)^2^∑^+^ with those published by Pototschnig et al.^38^ shows an acceptable agreement with a relative error 1% ≤ Δμ_e_/μ_e_ ≤ 19.8%. The exception goes when the calculations were performed by using the ab initio CASPT2 method, with a relative error 19.5% ≤ Δμ_e_/μ_e_ ≤ 45.4%.

### Transition Dipole Moment Curves and Radiative Lifetimes

**Figure 9:**
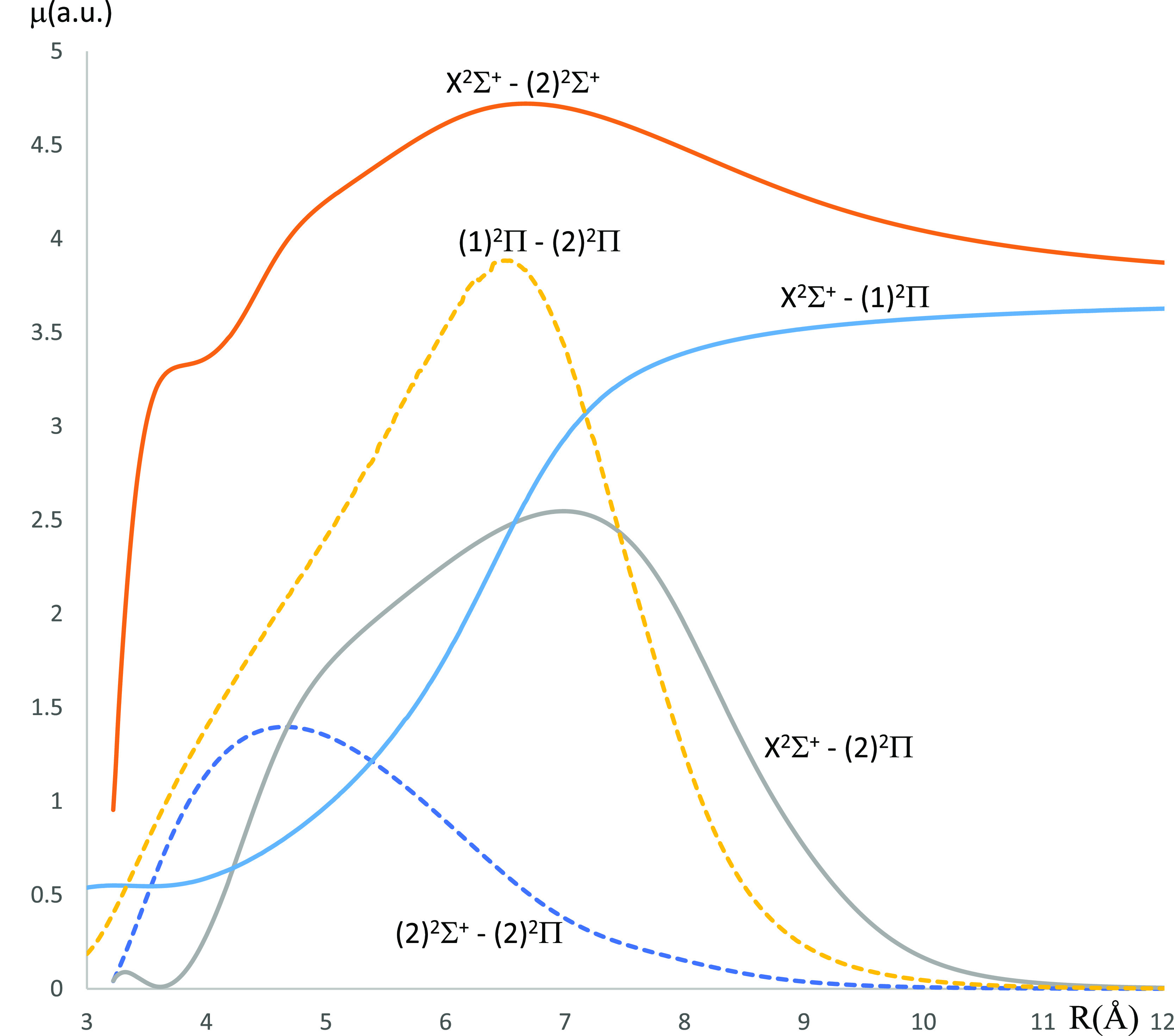
Transition D.M.C.s between the ground state X^2^Σ^+^ and the lowest-excited doublet states of the CaCs molecule using the CASSCF/MRCI method with three valence electrons.

**Figure 10:**
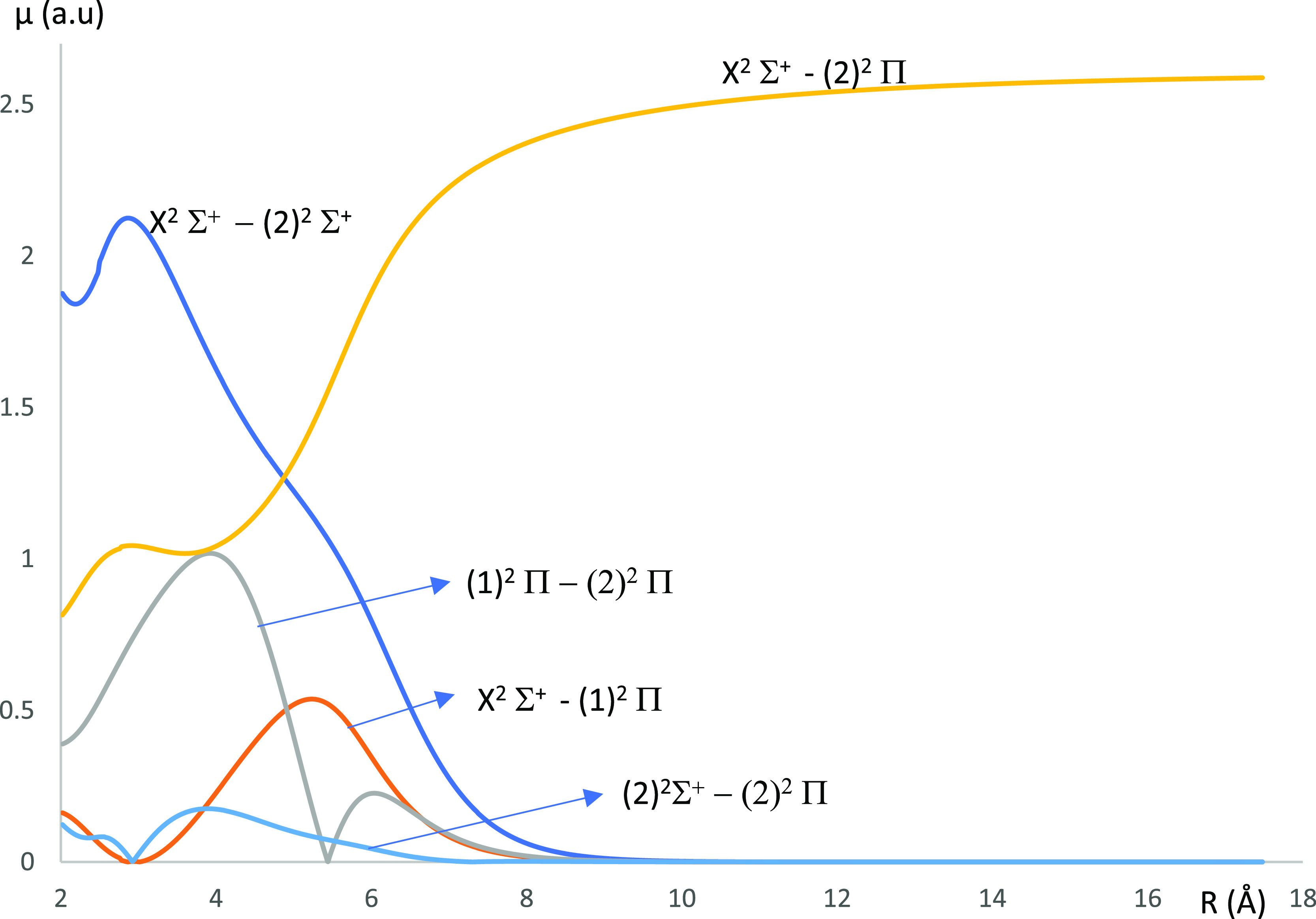
Transition D.M.C.s between the ground state X^2^Σ^+^ and the lowest-excited doublet states of the CaNa molecule using the CASSCF/MRCI method with three valence electrons.

The T.D.M. is useful for predicting the possible transitions that are likely to occur between certain electronic states. In our work, we investigated and show in Figures 9 and 10 the transition dipole moment curves (TDMCs) of the allowed transitions from the lowest excited to the ground X^2^Σ^+^ states for the molecules CaCs and CaNa as a function of the internuclear distance.

For the molecule CaCs, the TDMCs for the transitions X^2^Σ^+^–(2)^2^Π, (1)^2^Π–(2)^2^Π, and (2)^2^Σ^+^–(2)^2^Π vanish when *R* is larger than 10.5 Å where the occurrence of these transitions is at a very low probability. For the two transitions X^2^Σ^+^–(1)^2^Π and X^2^Σ^+^–(2)^2^Σ^+^, the transitions are maximal for *R* greater than 11 and 5.54 Å, respectively. The TDMCs for the X^2^Σ^+^–(2)^2^Σ^+^, X^2^Σ^+^–(1)^2^Π, (2)^2^Σ^+^–(2)^2^Π, and (1)^2^Π–(2)^2^Π transitions of the CaNa molecule vanish when *R* is larger than 9 Å. The transition X^2^Σ^+^–(2)^2^Π is the highest for *R* greater than 16 Å. We have considered the T.D.M. values at the equilibrium position *R*_e_ of the upper state for each electronic transition to calculate the emission coefficients proposed by Hilborn^39^ for the two considered molecules, CaCs and CaNa. The T.D.M. value|μ_21_| and the radiative lifetime τ_21_ ( where *j* runs for the underlying states of the *i* state), the emission angular frequency ω_21_, the Einstein coefficients of spontaneous emissions *A*_21_, the oscillator strength constant |*f*_21_|, and the classical radiative decay rate of the single-electron oscillator γ_cl_ are presented in Table 5. The emission coefficients for the allowed electronic transitions are given below, where ν_*ij*_ is the transition frequency between the two states, ε_0_ is the vacuum permittivity, and m_e_ is the mass of an electron
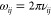
1.1
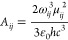
1.2
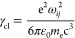
1.3
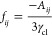
1.4

**Table 5:** Transition Dipole Moment Values of the Upper State at its Equilibrium Position |μ|, the Emission Angular Frequency ω_21_, the Einstein Spontaneous Coefficients *A*_21_, the Spontaneous Radiative Lifetime τ_spon_, the Classical Radiative Decay Rate of the Single-Electron Oscillator γ_cl_, and the Emission Oscillator Strength *f*_21_ of Some Transitions among the Doublet States of CaCs and CaNa Molecules

transition	|μ_21_| (au)	ω_21_ × 10^–15^ (rad s^–1^)	*A*_21_ (s^–1^)	τ_21_ (ns)	γ_cl_ × 10^–6^ (s^–1^)	|*f*_21_|
CaCs						
X^2^Σ^+^–(1)^2^Π	0.655	0.760	56 934.23	17 564.12	3.61	0.00526
X^2^Σ^+^–(2)^2^Π	1.698	2.055	7 573 195.06	132.04	26.41	0.09557
(2)^2^Σ^+^–(2)^2^Π	1.355	0.768	253 461.85	3945.37	3.70	0.02280
(1)^2^Π–(2)^2^Π	2.386	1.295	3 743 637.51	267.12	10.49	0.11893
X^2^Σ^+^–(3)^2^Σ^+^	2.471	2.343	23 763 471.39	42.08	34.32	0.23078
X^2^Σ^+^–(2)^2^Σ^+^	4.595	1.286	13 572 618.85	73.68	10.33	0.43783
CaNa						
X^2^Σ^+^–(1)^2^Π	0.037	1.238	769.05	1 300 317.3	9.57	0.000027
X^2^Σ^+^–(2)^2^Π	1.018	2.581	5 389 790.30	185.6	41.65	0.043144
(2)^2^Σ^+^–(2)^2^Π	0.159	0.817	4120.94	242 666.3	4.17	0.000329
(1)^2^Π–(2)^2^Π	0.968	1.344	686 368.83	1456.7	11.29	0.020275
X^2^Σ^+^–(3)^2^Σ^+^	3.881	2.616	81 540 743.90	12.3	42.79	0.635332
X^2^Σ^+^–(2)^2^Σ^+^	1.668	1.765	4 621 351.20	216.4	19.48	0.079116

The comparison of our data with previous work is absent since it is calculated here for the first time.

The oscillator strength expresses the probability of absorption or emission of electromagnetic radiation in transitions between energy levels of a molecule. If an emissive state has a small oscillator strength, nonradiative decay will outpace radiative decay. Conversely, “bright” transitions will have large oscillator strengths. From Table 5, we found that the largest oscillator strength belongs to the (X)^2^Σ^+^–(3)^2^Σ^+^ transition of the molecule CaNa and the most considerable value of the radiative lifetime is for the transition (X)^2^Σ^+^–(1)^2^Π of the same molecule. Since we are interested in the transition (X)^2^Σ^+^–(1)^2^Π for the laser cooling of the molecule CaCs, one can notice that the oscillator strength of this molecule is larger than that of the molecule CaNa, while the radiative lifetime is shorter. With these two conditions, the molecule CaCs is more advantageous for experimental laser cooling than the molecule CaNa.

### Vibration–Rotation Calculation

The vibrational energy *E*_v_, the rotational constant *B*_v_, and the centrifugal distortion constant *D*_v_ for the ground and many excited electronic states of the CaCs and CaNa molecules are determined by using the canonical functions approach^40,41^ and the cubic spline interpolation between each two consecutive points of the P.E.C obtained from the ab initio calculation of the molecule. Then, the calculated vibrational eigenvalues of energy and the P.E.C of the investigated states are used to determine the abscissas of the turning points *R*_min_ and *R*_max_ for each vibrational level. The calculations were done for a large number of vibrational levels up to *v* = 100 for deep well potential, while few vibrational levels were calculated for shallow well potentials. However, these calculations cannot be achieved for some electronic states due to crossings and avoided crossing in the P.E.C near the minima, the existence of double minima, and for very shallow potentials.

The vibrational constants of the investigated electronic states of the two molecules CaCs and CaNa are collected and presented in Tables S2 and S3 in the Supporting Information. There is no comparison with other data for the ground and the excited states of the molecules CaCs since they are investigated here for the first time. For the ground state (Χ)^2^∑^+^ of the CaNa molecule, the rotational constants *B*_v_ of five vibrational levels have been found in the literature. The comparison of our calculated values of these constants with those given in the literature shows a very good agreement with the relative difference 0.02%^28^ ≤ Δ*B*_v_/*B*_v_ ≤ 2.2%.^28^ The comparison for the other vibrational constants of the investigated electronic states of the molecule CaNa is absent since they are calculated here for the first time. These theoretical data will be a good guide for the spectroscopic experimentalists, particularly the values of *R*_min_ and *R*_max_ that will be compared with the values of the experimental Rydberg–Klein–Rees potentials.

### Laser-Cooling Study

#### Laser-Cooling Viability of CaCs Molecule

The small difference in equilibrium positions Δ*R*_e_ between the two electronic X^2^Σ^+^ and (2)^2^Σ^+^ states and X^2^Σ^+^ and (2)^2^Π states of the CaCs molecule directed our attention to study the laser-cooling feasibility for this molecule. The primary criteria for direct laser cooling is a highly diagonal Franck–Condon factors (FCFs) between the ground and a low-lying excited electronic state. This allows the use of a limited number of lasers to keep the molecule in a closed-loop cycle.^42^ The second criterion that affects a molecule’s laser-cooling viability is a short radiative lifetime between the vibrational levels of the involved electronic states. In the present work, a direct laser cooling is studied between the two electronic X^2^Σ^+^ and (2)^2^Π states in the presence of the intervening electronic states (1)^2^Π and (2)^2^Σ^+^ between them.

An examination of the table of the spectroscopic constants of the CaNa molecule shows a large difference between the values of the internuclear distance at equilibrium *R*_e_ for the ground electronic state and that of higher excited states. Such a large difference usually implies a non-diagonal FCF between the involved states. Consequently, we aborted further investigations of CaNa laser cooling at this stage.

**Figure 11:**
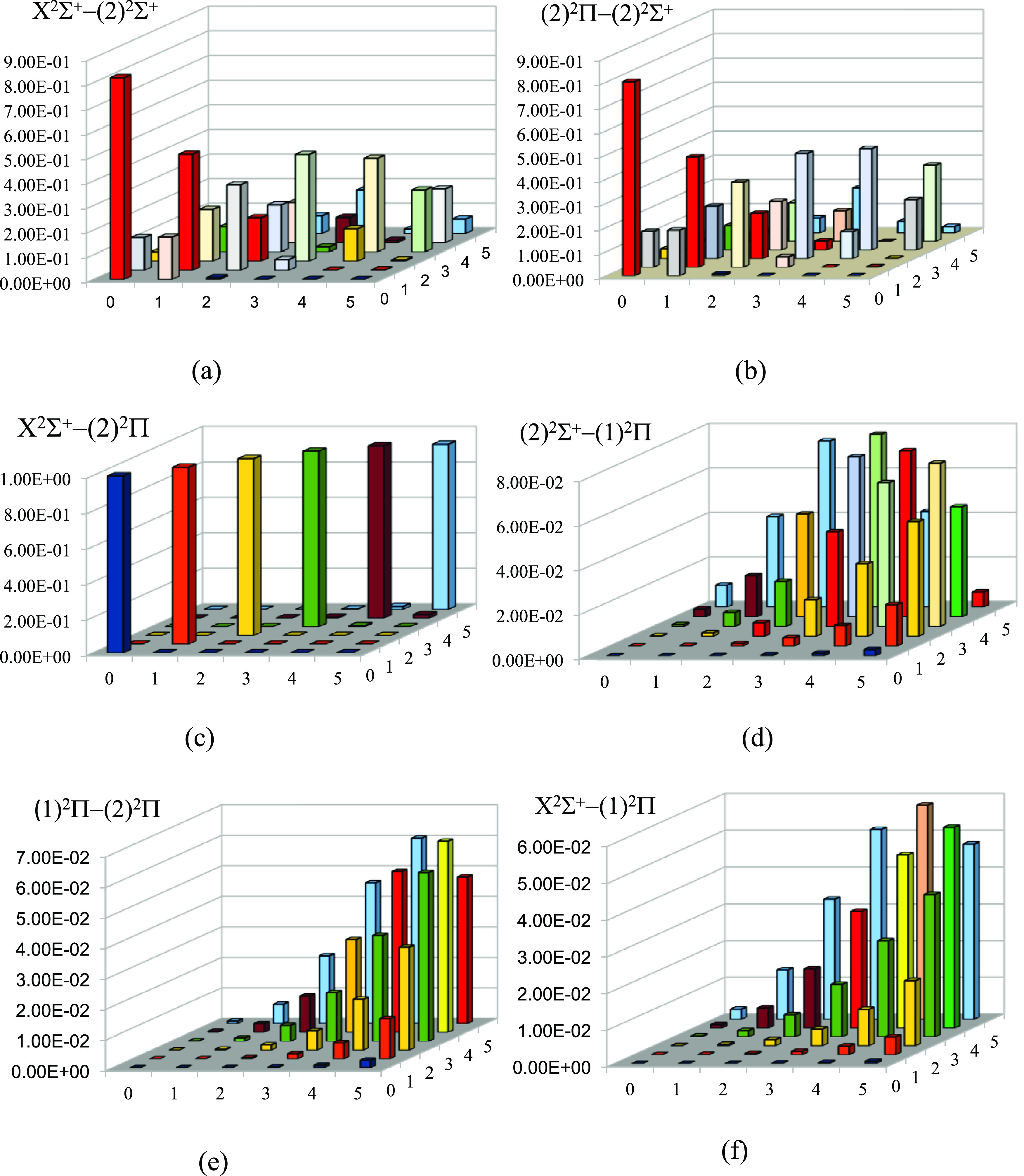
FCF plotting for the transitions (a) (2)^2^Σ^+^–X^2^Σ^+^, (b) (2)^2^Π(2)–^2^Σ^+^, (c) X^2^Σ^+^–(2)^2^Π, (d) (2)^2^Σ^+^–(1)^2^Π, (e) (2)^2^Π–(1)^2^Π, and (f) X^2^Σ^+^–(1)^2^Π of the CaCs molecule using the CASSCF/MRCI method with three valence electrons.

By using the LEVEL 11 program,^43^ we have calculated the FCFs for the transitions X^2^Σ^+^–(1)^2^Π, X^2^Σ^+^–(2)^2^Π, X^2^Σ^+^–(2)^2^Σ^+^, (1)^2^Π–(2)^2^Π, (2)^2^Σ^+^–(2)^2^Π, (2)^2^Σ^+^–(1)^2^Π of the CaCs molecule at the vibrational levels 0 ≤ *v*″ ≤ 5 of the upper states (2)^2^Π and 0 ≤ *v* ≤ 5 of the lower states X^2^Σ^+^. The graphical representation of the FCF for the cited transitions is shown in Figure 11, and their corresponding values are set in Table S4 in the Supporting Information.

We can notice by checking Figure 11c and the values in Table S5 in the Supporting Information that the FCFs for the six lowest vibrational levels of the transition (2)^2^Π → X^2^Σ^+^ of the molecule CaCs are diagonal (*f*_00_ = 0.998, *f*_11_ = 0.999, *f*_22_ = 0.998, *f*_33_ = 0.991, *f*_44_ = 0.972, and *f*_55_ = 0.935). This diagonal nature has also been proven experimentally and theoretically.^28,38,44^ Based on these values of FCFs, we study the direct laser cooling for this transition (2)^2^Π → X^2^Σ^+^. The intermediate state (1)^2^Π has no effect on the cycling cooling of this transition since the FCFs for the transitions (2)^2^Σ^+^–(1)^2^Π, X^2^Σ^+^–(1)^2^Π, and (2)^2^Π–(1)^2^Π shown in Figure 11d–f, respectively, are minimal for the lowest vibrational levels. The intermediate (2)^2^Σ^+^ state cannot be ignored since the FCFs for the transitions X^2^Σ^+^–(2)^2^Σ^+^ (Figure 11a) and (2)^2^Σ^+^–(2)^2^Π (Figure 11b) are significant for the first three vibrational levels. The laser cooling of molecules with a non-intervening intermediate electronic state between the cycling levels has been confirmed for several molecules, with specific requirements.^42−46^ These include a higher transition probability and a smaller radiative lifetime for transitions between the electronic excited and the ground states than the intermediate one. Completing these criteria would assure that an intermediate state does not hinder the laser-cooling cycle. Recently, however, Yuan et al.,^47^ Nguyen and Odom,^48^ and Li et al.^49^ have proposed laser-cooling schemes involving intervening electronic intermediate states in the cooling cycle. In the following, we show that within the approximation of spin-free calculations, the CaCs molecule is suitable for laser cooling through the cycle X^2^Σ^+^–(2)^2^Π with the involvement of the intermediate state (2)^2^Σ^+^ in the laser-cooling process.

To apply a three-step cooling scheme, one has to investigate the radiative lifetime, the value of the FCFs, and vibrational loss ratio between the ground, excited, and intervening states. We denote by *v* the vibrational states belonging to the ground state X^2^Σ^+^, ν′ are the ones belonging to the intermediate state (2)^2^Σ^+^, and ν″ belongs to the excited states involved in the cooling loop (2)^2^Π. To obtain the values of the radiative lifetime τ, the LEVEL program can be used to calculate the Einstein coefficients^42^ of the ro–vibrational transitions through the formula^45^
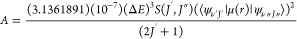
2μ(*r*) is the electronic T.D.M. (in debye),^46^*A*_ν″ν_ is the Einstein coefficient in s^–1^, Δ*E* is the emission frequency (in cm^–1^), *J* is the rotational quantum number, and *S*(*J*′, *J*″) is the Hönl–London factor whose values vary with the nature of the electronic transition. The present version of the program calculates *A* only in the case of singlet–singlet transitions. Given that the states we are dealing with are doublets, we will be using the following vibrational approximation instead (consider Λ as the projection of the angular momentum of an electronic state on the internuclear axis). For the parallel transitions with ΔΛ = 0, such as the transition X^2^Σ^+^–(2)^2^Σ^+^, we use
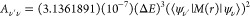
3

For the perpendicular transitions with ΔΛ = ±1, such as X^2^Σ^+^–(2)^2^Π and (2)^2^Σ^+^–(2)^2^Π, the recommended definition of the perpendicular T.D.M.^50^ is usually represented by
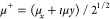
4.1
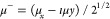
4.2

In the present work, however, we used the T.D.M. functions in MOLPRO software as μ_*x*_, μ_*y*_, and μ_*z*_ where the calculated T.D.M. is vertical (given with respect to *x*, *y*, and *z*). In this case, the Einstein coefficients^50^ are divided by 2 and given by
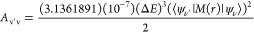
5

The calculated values of the radiative lifetime  for the transitions X^2^Σ^+^–(2)^2^Σ^+^, (2)^2^Π–(2)^2^Σ^+^, and X^2^Σ^+^––(2)^2^Π are given in Table 5. We find a strong correspondence between the value of τ among electronic transitions (calculated in Table 5) and the vibrational state transitions τ_0_ (Table 6). More specifically, for the transitions X^2^Σ^+^–(2)^2^Π, (2)^2^Σ^+^–(2)^2^Π, and X^2^Σ^+^–(2)^2^Σ^+^, the electronic transition radiative lifetimes τ are 132.04, 3945.37, and 73.68 ns, respectively; the values of τ_0_ for the same transitions are 136.5, 4050, and 71.8 ns. The highly diagonal FCF (Table S5) for the transition X^2^Σ^+^–(2)^2^Π (Figure 11c) and the short value of the radiative lifetime 68.3 < τ_0_ < 71.4 ns are indicative of a possible laser-cooling scheme involving these two states.

**Table 6:** Radiative Lifetimes τ of the Vibrational Transitions between the Electronic States X^2^Σ^+^–(2)^2^Σ^+^, X^2^Σ^+^–(2)^2^Π, and (2)^2^Σ^+^–(2)^2^Π of the CaCs Molecule

X^2^Σ^+^–(2)^2^Σ^+^								
	value	ν′(2^2^Σ^+^) = 0	1	2	3	4	5	6
ν (X^2^Σ^+^) = 0	*A*_vv′_	1.151437 × 10^7^	1.962532 × 10^6^	5.683341 × 10^5^	1.114800 × 10^5^	2.341589 × 10^4^	4.344481 × 10^3^	8.972812 × 10^2^
	*R*_vv′_	8.266503 × 10^–1^	1.400070 × 10^–1^	4.046544 × 10^–2^	7.914280 × 10^–3^	1.868120 × 10^–3^	7.091100 × 10^–4^	1.613200 × 10^–4^
1	*A*_vv′_	2.317422 × 10^6^	6.674955 × 10^6^	3.148351 × 10^6^	1.573059 × 10^6^	4.793907 × 10^5^	1.421461 × 10^5^	3.500623 × 10^4^
	*R*_vv′_	1.663744 × 10^–1^	4.761911 × 10^–1^	2.241629 × 10^–1^	1.116759 × 10^–1^	3.824573 × 10^–2^	2.320121 × 10^–2^	6.293740 × 10^–3^
2	*A*_vv′_	8.454822 × 10^4^	4.775409 × 10^6^	2.473803 × 10^6^	2.889758 × 10^6^	2.520671 × 10^6^	1.148000 × 10^6^	4.570333 × 10^5^
	*R*_vv′_	6.069960 × 10^–3^	3.406776 × 10^–1^	1.761350 × 10^–1^	2.051521 × 10^–1^	2.010988 × 10^–1^	1.873776 × 10^–1^	8.216958 × 10^–2^
3	*A*_vv′_	5.354361 × 10^3^	5.721896 × 10^5^	6.013017 × 10^6^	2.735225 × 10^5^	1.570320 × 10^6^	2.758580 × 10^6^	1.884728 × 10^6^
	*R*_vv′_	3.844000 × 10^–4^	4.081999 × 10^–2^	4.281274 × 10^–1^	1.941813 × 10^–2^	1.252799 × 10^–1^	4.502579 × 10^–1^	3.388535 × 10^–1^
4	*A*_vv′_	6.403610 × 10^3^	4.299750 × 10^–1^	1.752144 × 10^6^	5.339973 × 10^6^	1.515287 × 10^5^	2.990324 × 10^5^	1.983343 × 10^6^
	*R*_vv′_	4.597300 × 10^–4^	3.000000 × 10^–8^	1.247528 × 10^–1^	3.790998 × 10^–1^	1.208894 × 10^–2^	4.880833 × 10^–2^	3.565834 × 10^–1^
5	*A*_vv′_	8.483919 × 10^2^	2.365979 × 10^4^	5.973875 × 10^4^	3.435269 × 10^6^	3.099584 × 10^6^	9.225905 × 10^5^	6.341689 × 10^4^
	*R*_vv′_	6.091000 × 10^–5^	1.687890 × 10^–3^	4.253410 × 10^–3^	2.438794 × 10^–1^	2.472844 × 10^–1^	1.505860 × 10^–1^	1.140166 × 10^–2^
6	*A*_vv′_	3.535833 × 10^0^	8.639941 × 10^3^	2.953637 × 10^4^	4.628698 × 10^5^	4.689579 × 10^6^	8.519738 × 10^5^	1.137650 × 10^6^
	*R*_vv′_	2.500000 × 10^–7^	6.163700 × 10^–4^	2.102990 × 10^–3^	3.286043 × 10^–2^	3.741340 × 10^–1^	1.390599 × 10^–1^	2.045370 × 10^–1^
sum (s^–1^) = *A*_ν′ν_		1.392895 × 10^7^	1.401739 × 10^7^	1.404492 × 10^7^	1.408593 × 10^7^	1.253449 × 10^7^	6.126667 × 10^6^	5.562074 × 10^6^
τ (s) = 1/*A*_ν′ν_		7.180000 × 10^–8^	7.130000 × 10^–8^	7.120000 × 10^–8^	7.100000 × 10^–8^	7.980000 × 10^–8^	1.630000 × 10^–7^	1.800000 × 10^–7^
τ (ns)		71.8	71.3	71.2	71.0	79.8	1163.0	180.0

	value	ν′(2)^2^Π = 0	1	2	3	4	5	6
X^2^Σ^+^–(2)^2^Π								
ν(X^2^Σ^+^) = 0	*A*_vv′_	7.307537 × 10^6^	1.540002 × 10^1^	4.043620 × 10^2^	9.457135 × 10^1^	6.757200 × 10^0^	3.648095 × 10^–1^	7.106915 × 10^–1^
	*R*_vv′_	9.653976 × 10^–1^	2.020432 × 10^–6^	2.680556 × 10^–5^	6.233773 × 10^–6^	4.440631 × 10^–7^	2.398561 × 10^–8^	5.021044 × 10^–8^
1	*A*_vv′_	1.264076 × 10^4^	7.364731 × 10^6^	5.969834 × 10^3^	1.797929 × 10^3^	2.045633 × 10^2^	1.655454 × 10^1^	1.930394 × 10^0^
	*R*_vv′_	1.669969 × 10^–3^	9.662287 × 10^–1^	3.957462 × 10^–4^	1.185124 × 10^–4^	1.344329 × 10^–5^	1.088433 × 10^–6^	1.363826 × 10^–7^
2	*A*_vv′_	2.258488 × 10^3^	1.027514 × 10^4^	1.483069 × 10^7^	6.272968 × 10^4^	9.686437 × 10^3^	1.250687 × 10^3^	3.088913 × 10^1^
	*R*_vv′_	2.983685 × 10^–4^	1.348065 × 10^–3^	9.831410 × 10^–1^	4.134895 × 10^–3^	6.365639 × 10^–4^	8.223058 × 10^–5^	2.182321 × 10^–6^
3	*A*_vv′_	1.413538 × 10^2^	8.786911 × 10^3^	4.521870 × 10^1^	1.482928 × 10^7^	1.908601 × 10^5^	2.434150 × 10^4^	3.637003 × 10^3^
	*R*_vv′_	1.867423 × 10^–5^	1.152814 × 10^–3^	2.997592 × 10^–6^	9.774880 × 10^–1^	1.254276 × 10^–2^	1.600413 × 10^–3^	2.569547 × 10^–4^
4	*A*_vv′_	2.742864 × 10^1^	9.229474 × 10^1^	1.918221 × 10^4^	3.223685 × 10^4^	1.462070 × 10^7^	4.422058 × 10^5^	5.720593 × 10^4^
	*R*_vv′_	3.623594 × 10^–6^	1.210877 × 10^–5^	1.271608 × 10^–3^	2.124927 × 10^–3^	9.608291 × 10^–1^	2.907429 × 10^–2^	4.041606 × 10^–3^
5	*A*_vv′_	5.826010 × 10^–1^	9.546542 × 10^1^	3.824662 × 10^1^	3.266196 × 10^4^	1.738525 × 10^5^	1.409420 × 10^7^	8.486087 × 10^5^
	*R*_vv′_	7.696734 × 10^–8^	1.252475 × 10^–5^	2.535406 × 10^–6^	2.152948 × 10^–3^	1.142507 × 10^–2^	9.266697 × 10^–1^	5.995431 × 10^–2^
6	*A*_vv′_	2.285418 × 10^0^	5.437355 × 10^0^	2.191561 × 10^2^	1.304577 × 10^1^	4.475723 × 10^4^	5.057950 × 10^5^	1.310403 × 10^7^
	*R*_vv′_	3.019262 × 10^–7^	7.133633 × 10^–7^	1.452808 × 10^–5^	8.599260 × 10^–7^	2.941312 × 10^–3^	3.325517 × 10^–2^	9.258013 × 10^–1^
sum (s^–1^) = *A*_ν′ν_		7.322607 × 10^6^	7.384002 × 10^6^	1.485655 × 10^7^	1.495881 × 10^7^	1.504007 × 10^7^	1.506780 × 10^7^	1.401352 × 10^7^
τ (s) = 1/*A*_ν′ν_		1.365634 × 10^–7^	1.354279 × 10^–7^	6.731039 × 10^–8^	6.685022 × 10^–8^	6.648906 × 10^–8^	6.636667 × 10^–8^	7.135968 × 10^–8^
τ (ns)		136.6	135.4	67.3	66.8	66.5	66.4	71.3
(2)^2^Σ^+^–(2)^2^Π								
ν((2)^2^Σ^+^) = 0	*A*_vv′_	2.040594 × 10^5^	5.247195 × 10^4^	2.665403 × 10^3^	7.337515 × 10^1^	1.493461 × 10^2^	2.015362 × 10^1^	1.837800 × 10^–2^
	*R*_vv′_	8.266518 × 10^–1^	2.203419 × 10^–1^	1.166686 × 10^–2^	3.461200 × 10^–4^	8.452600 × 10^–4^	1.422200 × 10^–4^	1.300000 × 10^–7^
1	*A*_vv′_	3.296895 × 10^4^	1.129200 × 10^5^	9.423539 × 10^4^	1.278708 × 10^4^	3.367380 × 10^–1^	5.392491 × 10^2^	1.804288 × 10^2^
	*R*_vv′_	1.335584 × 10^–1^	4.741772 × 10^–1^	4.124822 × 10^–1^	6.031886 × 10^–2^	1.900000 × 10^–6^	3.805360 × 10^–3^	1.281980 × 10^–3^
2	*A*_vv′_	8.250505 × 10^3^	4.673072 × 10^4^	4.538750 × 10^4^	1.132751 × 10^5^	3.195216 × 10^4^	6.208191 × 10^2^	8.950309 × 10^2^
	*R*_vv′_	3.342308 × 10^–2^	1.962331 × 10^–1^	1.986678 × 10^–1^	5.343382 × 10^–1^	1.808413 × 10^–1^	4.380990 × 10^–3^	6.359380 × 10^–3^
3	*A*_vv′_	1.322416 × 10^3^	1.992522 × 10^4^	4.203309 × 10^4^	9.110008 × 10^3^	1.057596 × 10^5^	5.718606 × 10^4^	4.353858 × 10^3^
	*R*_vv′_	5.357150 × 10^–3^	8.367061 × 10^–2^	1.839850 × 10^–1^	4.297348 × 10^–2^	5.985731 × 10^–1^	4.035497 × 10^–1^	3.093507 × 10^–2^
4	*A*_vv′_	2.140005 × 10^2^	4.777781 × 10^3^	3.043745 × 10^4^	2.584151 × 10^4^	1.445372 × 10^1^	7.736191 × 10^4^	8.060838 × 10^4^
	*R*_vv′_	8.669200 × 10^–4^	2.006301 × 10^–2^	1.332292 × 10^–1^	1.218989 × 10^–1^	8.180000 × 10^–5^	5.459263 × 10^–1^	5.727394 × 10^–1^
5	*A*_vv′_	2.971031 × 10^1^	1.110592 × 10^3^	1.048777 × 10^4^	3.421640 × 10^4^	9.297499 × 10^3^	5.397876 × 10^3^	4.271001 × 10^4^
	*R*_vv′_	1.203600 × 10^–4^	4.663630 × 10^–3^	4.590651 × 10^–2^	1.614047 × 10^–1^	5.262153 × 10^–2^	3.809165 × 10^–2^	3.034636 × 10^–1^
6	*A*_vv′_	5.437040 × 10^0^	2.024822 × 10^2^	3.212693 × 10^3^	1.668792 × 10^4^	2.951271 × 10^4^	5.815654 × 10^2^	1.199406 × 10^4^
	*R*_vv′_	2.202000 × 10^–5^	8.502700 × 10^–4^	1.406243 × 10^–2^	7.871980 × 10^–2^	1.670346 × 10^–1^	4.103980 × 10^–3^	8.522031 × 10^–2^
sum (s^–1^) = *A*_ν′ν_		2.468505 × 10^5^	2.381388 × 10^5^	2.284593 × 10^5^	2.119914 × 10^5^	1.766862 × 10^5^	1.417076 × 10^5^	1.407418 × 10^5^
τ (s) = 1/*A*_ν′ν_		4.050000 × 10^–6^	4.200000 × 10^–6^	4.380000 × 10^–6^	4.720000 × 10^–6^	5.660000 × 10^–6^	7.060000 × 10^–6^	7.110000 × 10^–6^
τ (μs)		4.05	4.22	4.38	4.72	5.66	7.06	7.11

From the calculated Einstein coefficients, we additionally calculate the vibrational branching ratios by taking onto account the intermediate state for the three transitions using the following formulas^51,52^
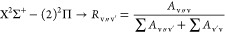
6.1
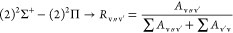
6.2
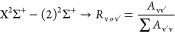
6.3

The obtained values are given in Table 6.

**Figure 12:**
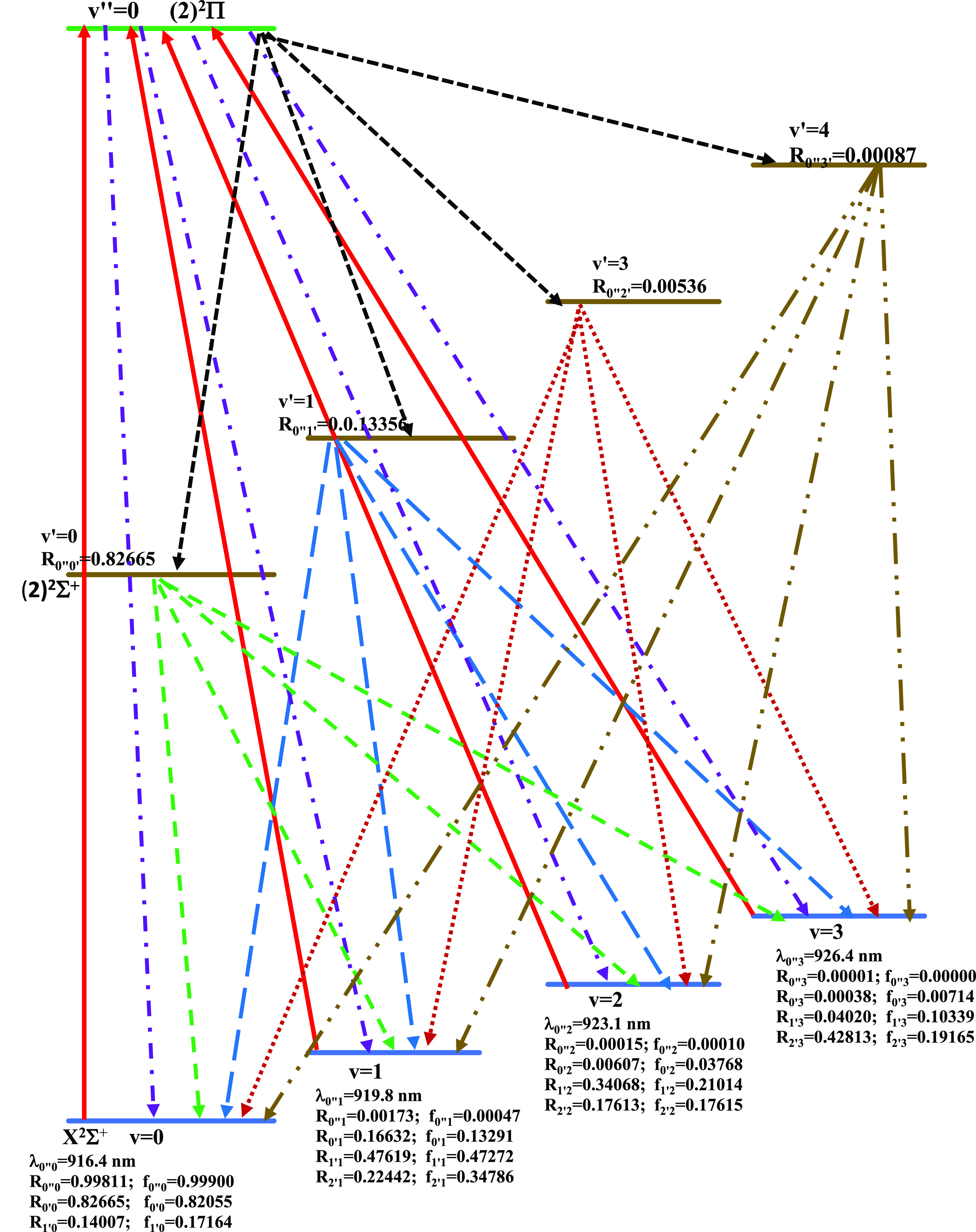
Laser-cooling scheme for the transition X^2^Σ^+^–(2)^2^Π οf the molecule CaCs with the intermediate state (2)^2^Σ^+^.

In setting out a laser-cooling scheme, the number of cycles (*N*) for photon absorption/emission should be maximized to sufficiently decelerate the molecule in the Doppler laser-cooling beams.^52,53^ The graphical representation of our proposed scheme is shown in Figure 12, where lasers are represented by red solid lines along with their wavelength. The spontaneous decays are represented by dotted lines with the values of their FCF (*f*_ν′ν_) and the vibrational branching ratios (*R*_ν′ν_). The wavelength of the main pumping laser (2)^2^Π(*v*″ = 0) ← X^2^Σ^+^(ν = 0) is λ_0″_ = 916.4 nm. Three re-pumping laser beams are employed to avoid leakage to lower vibrational levels. The wavelength of these repumping lasers for the transitions (2)^2^Π(*v*″ = 0) ← X^2^Σ^+^(ν = 1), (2)^2^Π(*v*″ = 0) ← X^2^Σ^+^ (ν = 2), and (2)^2^Π(*v*″ = 0) ← X^2^Σ^+^(ν = 3) are, respectively, λ_0″1_ = 918.8 nm, λ_0″2_ = 923.1 nm, and λ_0″3_ = 926.4 nm. In this case, *N*, which is the reciprocal to the total loss, is given by
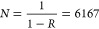
7.1where
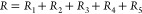
7.2
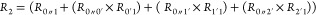
7.3
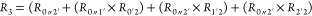
7.4
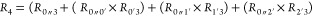
7.8
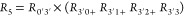
7.9

For more experimental detail, the parameters *L*, *a*_max_, *V*, and *T*, which are respectively the slowing distance, the maximum acceleration, the initial velocity, and temperature are^24,52^
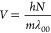
8.1
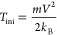
8.2
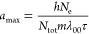
8.3
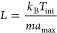
8.4where *K*_b_ and *h* are the Boltzmann and Planck constants and *m* is the mass of the considered CaCs molecule.

With this value of N, the experimental parameters given in eq 4.2 are *V* = 15.16 m/s, *a*_max_ = 7.37 × 10^3^ m/s^2^, and *L* = 1.56 cm. We have considered the ratio of the number of excited states connected to the ground state in the main cycling transition (*N*_e_) to the number of ground states connected to the excited state of the leading cycling transition (*N*_tot_) plus *N*_e_. We have approximated the value of *N*_e_/*N*_tot_ = 1/5 by only taking into account the vibrational ground and excited states and ignoring any hyperfine structure. The value of the slowing distance *L* can be considered as a convenient choice as it is within the range of the experimental values for a typical laser cooling setup. By using the calculated experimental parameters of eq 4.2*V* = 15.19 m/s, *T*_ini_ = 2.51 K and *a*_max_ = 7.37 × 10^3^, we find that the Doppler and Recoil temperatures that can be reached during the cooling process are^24,54^
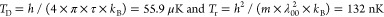
9

The molecule’s initial velocity and temperature imply that one needs to find a cooling process that would lead to the initial temperature of 2.51 K before it reaches the nanokelvin regime. Buffer gas cooling is a flexible method that is applicable to a multitude of molecules. It consists of thermalizing species through collisions with a cold buffer gas, whose role is to dissipate the molecules’ translational energy. Buffer gas cooling of calcium-bearing molecules has been proven successful for species such as CaH, which were cooled to temperatures close to microkelvin.

We model the CaCs molecules to be produced through a typical laser ablation technique before being driven into a buffer cooling cell, to be then sent in the Doppler laser-cooling setup. According to the hard-sphere collision model, after *N* collisions in the buffer gas cell, the molecules are thermalized to the temperature *T*_N_, which is given by^52^
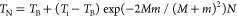
10We consider the initial temperature *T*_i_ = 7000 K as the typical temperature of the CaCs molecules as they leave the laser ablation setup, *T*_B_ = 2 K is the initial temperature of the helium gas in the buffer gas cell, and *T*_N_ = 2.51 K is the precooling temperature of CaCs molecules. From eq 6.1, one can find the number of collisions in the buffer cell *N* = 224.

For a low density of CaCs molecules, the average distance λ (mean free path) covered by the molecules between *N* and *N* – 1 collisions with the helium gas of the buffer cell is given by^52^
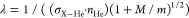
11

At a low helium density of *n*_He_ = 5 × 10^14^ atom/cm^3^ and at low temperature, the scattering cross-sectional value for collisions between CaCs molecules and He atoms is typically close to σ_X–He_ = 10^–14^ cm^2^, leading to a value of λ = 0.0295 cm. By using the rules of the kinetic theory of ideal gases, the time for thermalizing the molecules of the CaCs in the buffer cell is then given by^24^
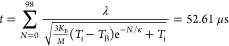
12where *K*_B_ is the Boltzmann constant and .

## Conclusions

In this paper, we have reported ab initio calculations of 25 doublet and quartet states of the CaCs molecule and 32 doublet and quartet low-lying energy states of the CaNa molecule. We studied the P.E.C.s and D.M.C.s of these molecules with three valence electrons at the spin-free level by using the CASSCF/MRCI method with the basis sets ECP46MWB and ECP10MWB for Cs and Ca atoms, respectively, while for the Na atom, we used the two basis aug-ccpVAZ and ccpVAZ. In addition, the PDMs for the ground and the excited electronic states have been calculated and for most of the bound states, the spectroscopic constants *T*_e_, ω_e_, *B*_e_, *R*_e_, and *D*_e_ have been also obtained. Moreover, the ro-vibrational constants *E*_v_, *B*_v_, and *D*_v_ with the abscissas of turning points *R*_min_ and *R*_max_ have been obtained for different vibrational levels of the ground state and some low-lying electronic states of the two molecules by means of the canonical function approach. The T.D.M.s have been also determined for the lowest electronic transitions, along with the emission angular frequency ω_21_ and the oscillator strength *f*_21_. The calculation of the FCF, the Einstein coefficients , and the spontaneous radiative lifetime for the molecule CaCs shows its candidacy for a direct laser cooling between the two electronic states X^2^Σ^+^ and (2)^2^Π. The study of this cooling has been done with the intermediate states (1)^2^Π and (2)^2^Σ^+^ by calculating the vibrational branching ratios, the number of cycles (*N*) for photon absorption/emission, the experimental parameters of this cooling, and the recoil and Doppler temperatures. The values of the initial required temperature show the need for a precooling buffer gas cell, in a typical experimental setup. A laser cooling scheme is presented with four pumping and repumping lasers whose wavelengths are in the infrared region. These results open the way for an experimental work on the cooling of the transition X^2^Σ^+^–(2)^2^Π of the CaCs molecule, with an intermediate state. Cooling polar molecules such as CaCs to the microkelvin and nanokelvin range of temperature could lead to phenomena and discoveries far beyond the focus of traditional molecular science. More precisely, such studies offer promising applications such as new platforms for quantum computing, precise control of molecular dynamics, nanolithography, and Bose–Einstein condensate of a polar molecule. The electric dipole–dipole interaction may give rise to a molecular superfluid via Bardeen–Cooper–Schrieffer pairing, and it leads to fundamentally new condensed-matter phases and new complex quantum dynamics.^55^

## Supporting Information Available

Potential energy curves of the lowest ^2^Σ^+^, ^2^Π, and ^2^Δ electronic states of the CaNa molecule with the Aug-cc-pVQZ basis for the Na atom using the CASSCF/MRCI method with three valence electrons; permanent D.M.C.s of the doublet and quartet states of CaCs and CaNa; position of the crossings *R*_c_ and avoided crossing *R*_AC_ with the energy difference Δ*E* of the molecule two molecules CaCs and CaNa; vibrational parameters of the excited states of the CaCs and CaNa molecules; and FCF for the lowest transitions X^2^Σ^+^–(1)^2^Π, X^2^Σ^+^–(2)^2^Σ^+^, (1)^2^Π–(2)^2^Π, (2)^2^Σ^+^–(2)^2^Π, and X^2^Σ^+^–(2)^2^Π of the CaCs molecule (PDF)
