# Supplementary material for: Laser Cooling with an Intermediate State and Electronic Structure Studies of the Molecules CaCs and CaNa
Source: ACS Omega. 2022 May 24;7(22):18577–96. doi: 10.1021/acsomega.2c01224 (PMC9178733; doi:10.1021/acsomega.2c01224)
Supplement: Supplementary file 1 — ao2c01224_si_001.pdf [file ao2c01224_si_001.pdf]

Laser cooling with an intermediate state and electronic structure studies of the molecules CaCs and CaNa

Amal Moussa,[1] Nayla El-Kork,*[,2,3] Israa Zeid,[1] Ehab Salem,[1] and Mahmoud Korek[1]

[1]Faculty of Science, Beirut Arab University, P.O. Box 11-5020 Riad El Solh, Beirut 1107 2809, Lebanon

[2]Department of Physics, Khalifa University, P.O. Box 127788, Abu Dhabi, United Arab Emirates

[3] Space and Planetary Science Center, Khalifa University, Abu Dhabi, United Arab Emirates

Keywords: *ab initio* calculation, electronic structure, rovibrational calculations, Franck-Condon factor, radiative lifetime, vibrational branching ratio, laser cooling scheme.

Journal: ACS-Omega

---

* Author to whom correspondence should be addressed

E-mail: nayla.elkork@ku.ac.ae

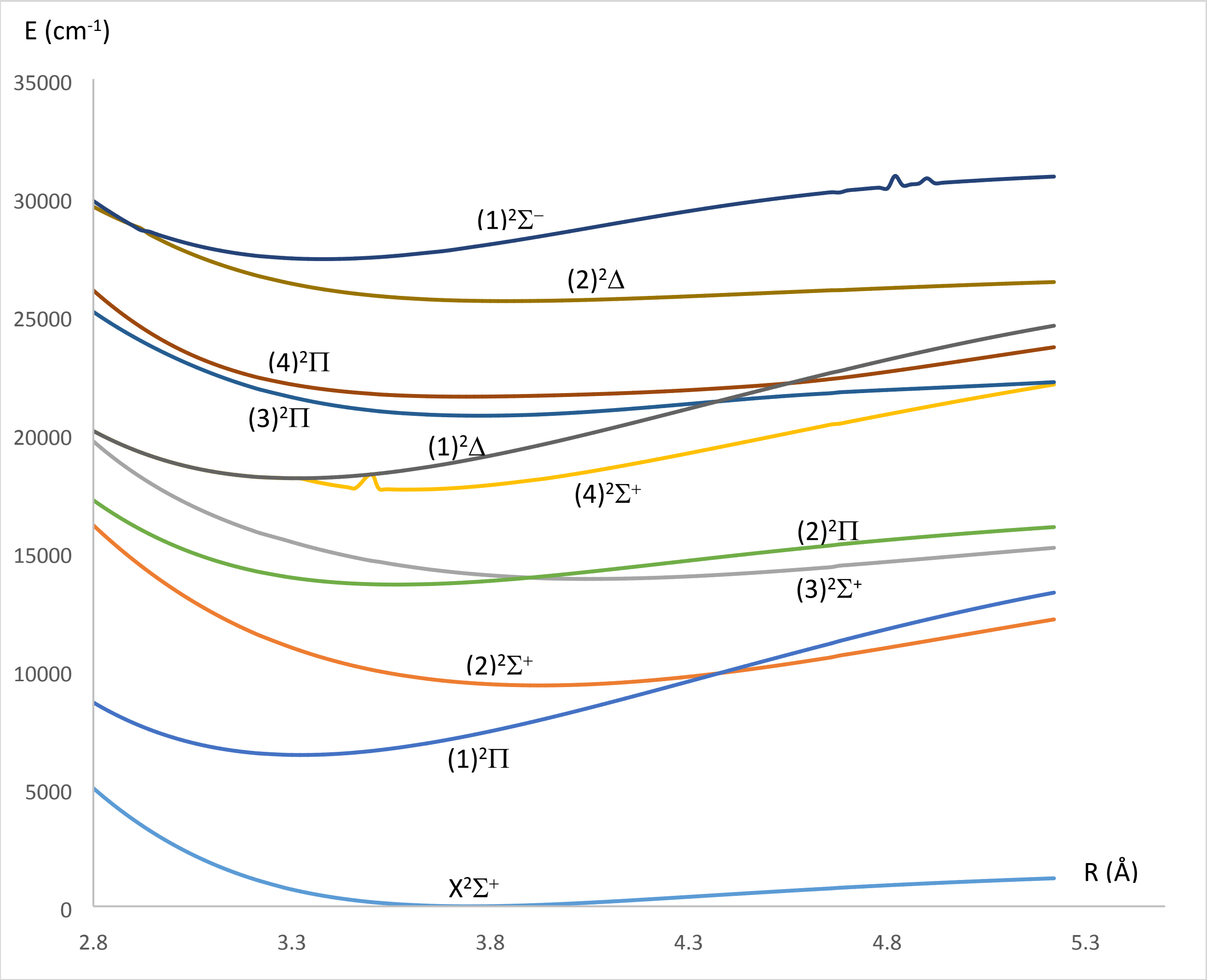

Figure S1: Potential energy curves of the lowest $^2\Sigma^+$, $^2\Pi$ and $^2\Delta$ electronic states of the CaNa molecule with Aug-cc-pVQZ basis for the Na atom using CASSCF/MRCI method with 3 valence electrons.

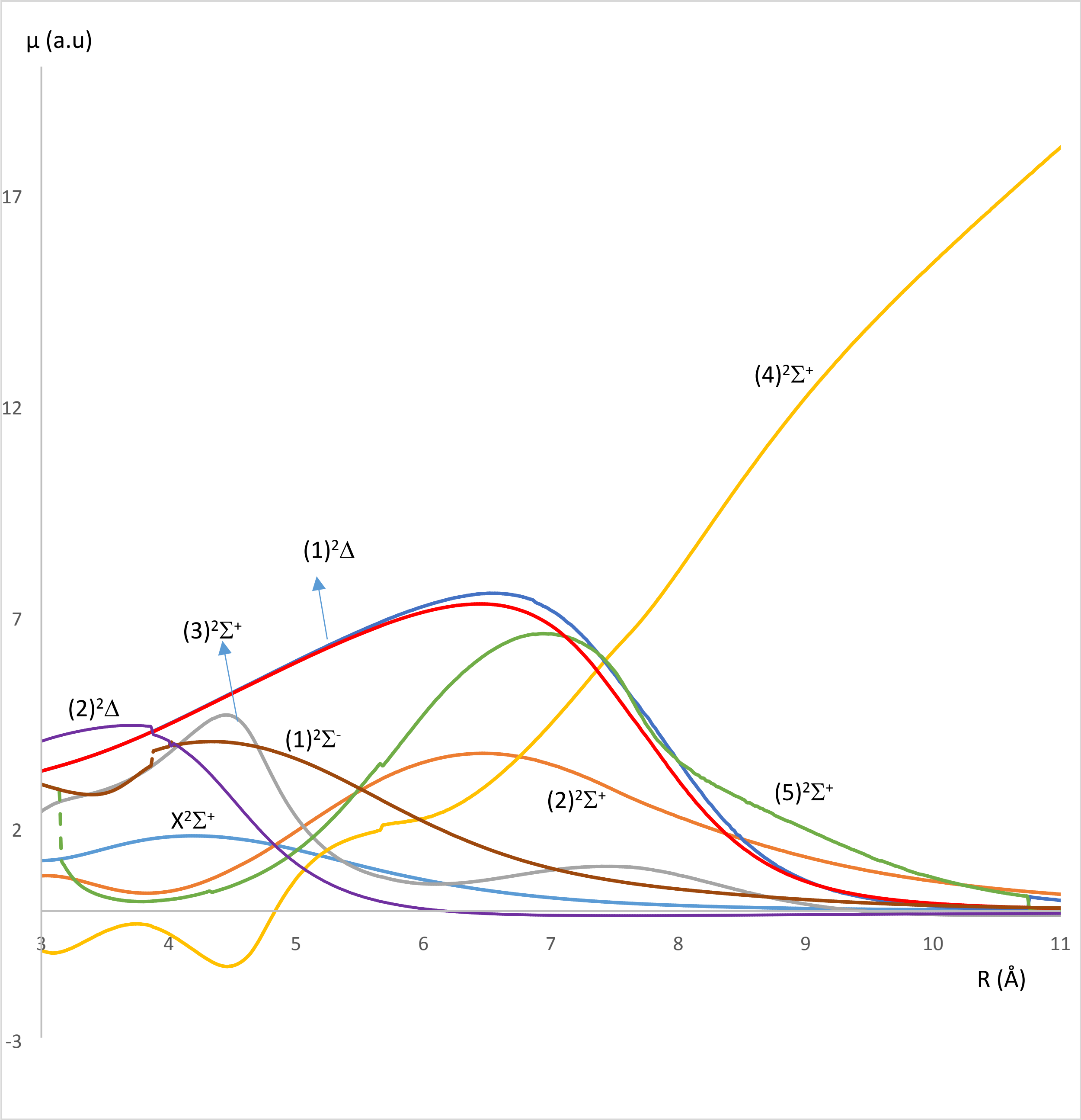

Figure S2: Dipole moment curves of the low-lying doublet states of symmetry one and four of CaCs molecule using CASSCF/MRCI method with 3 valence electrons.

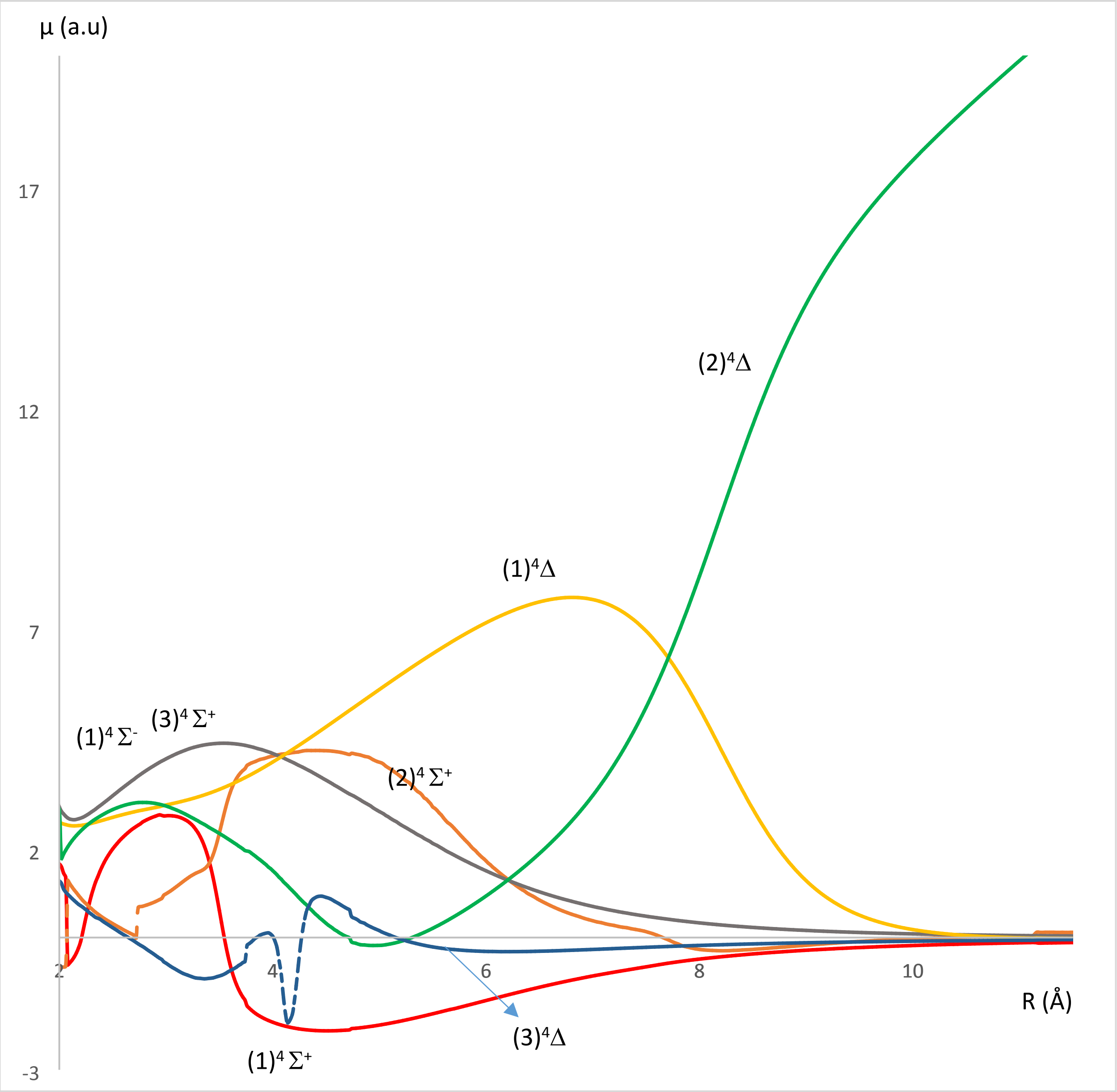

Figure S3: Dipole moment curves of the low-lying quartet states of symmetry one and four of CaCs molecule using CASSCF/MRCI method with 3 valence electrons.

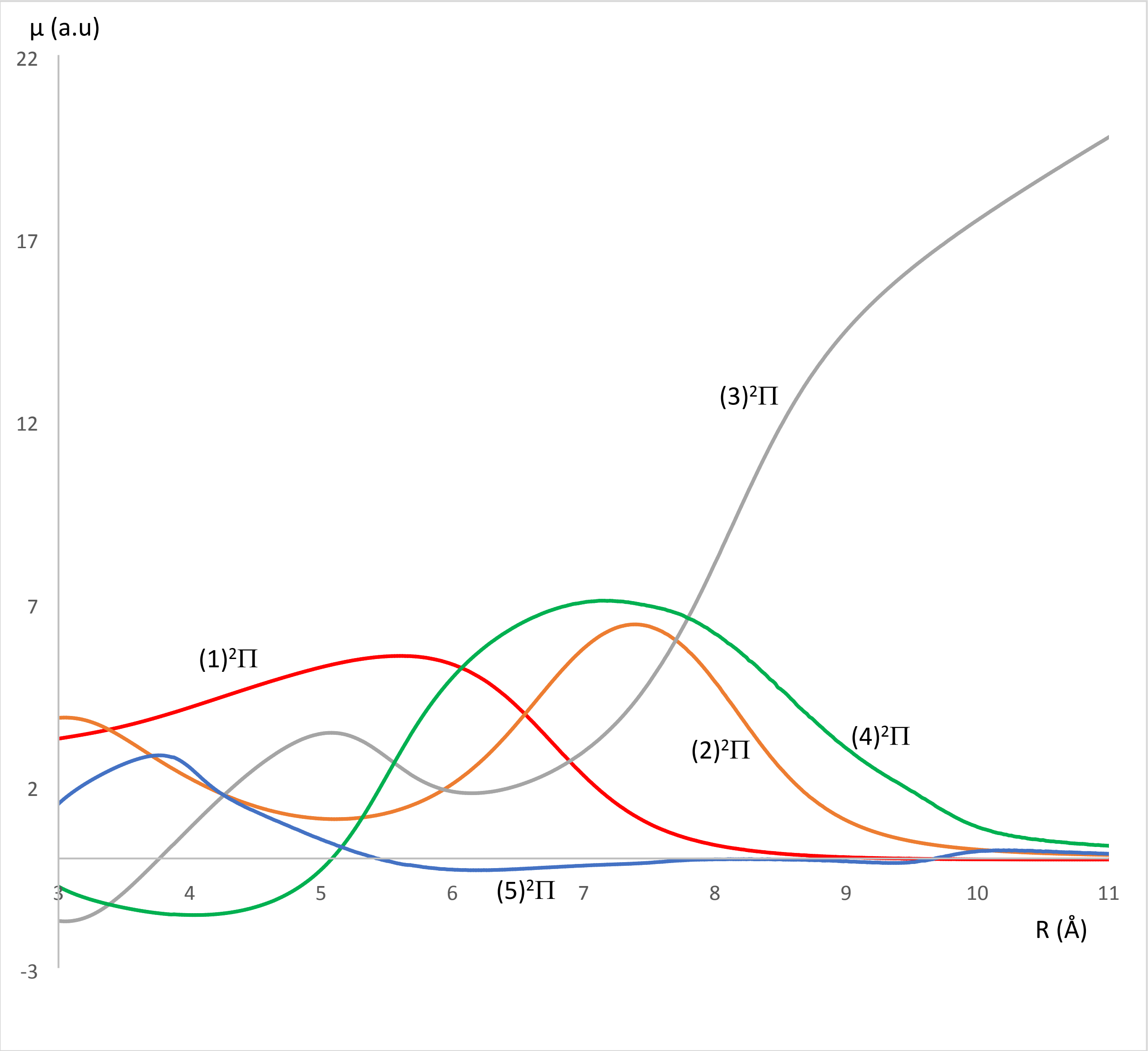

Figure S4: Dipole moment curves of the low-lying doublet states of symmetry two of CaCs molecule using CASSCF/MRCI method with 3 valence electrons.

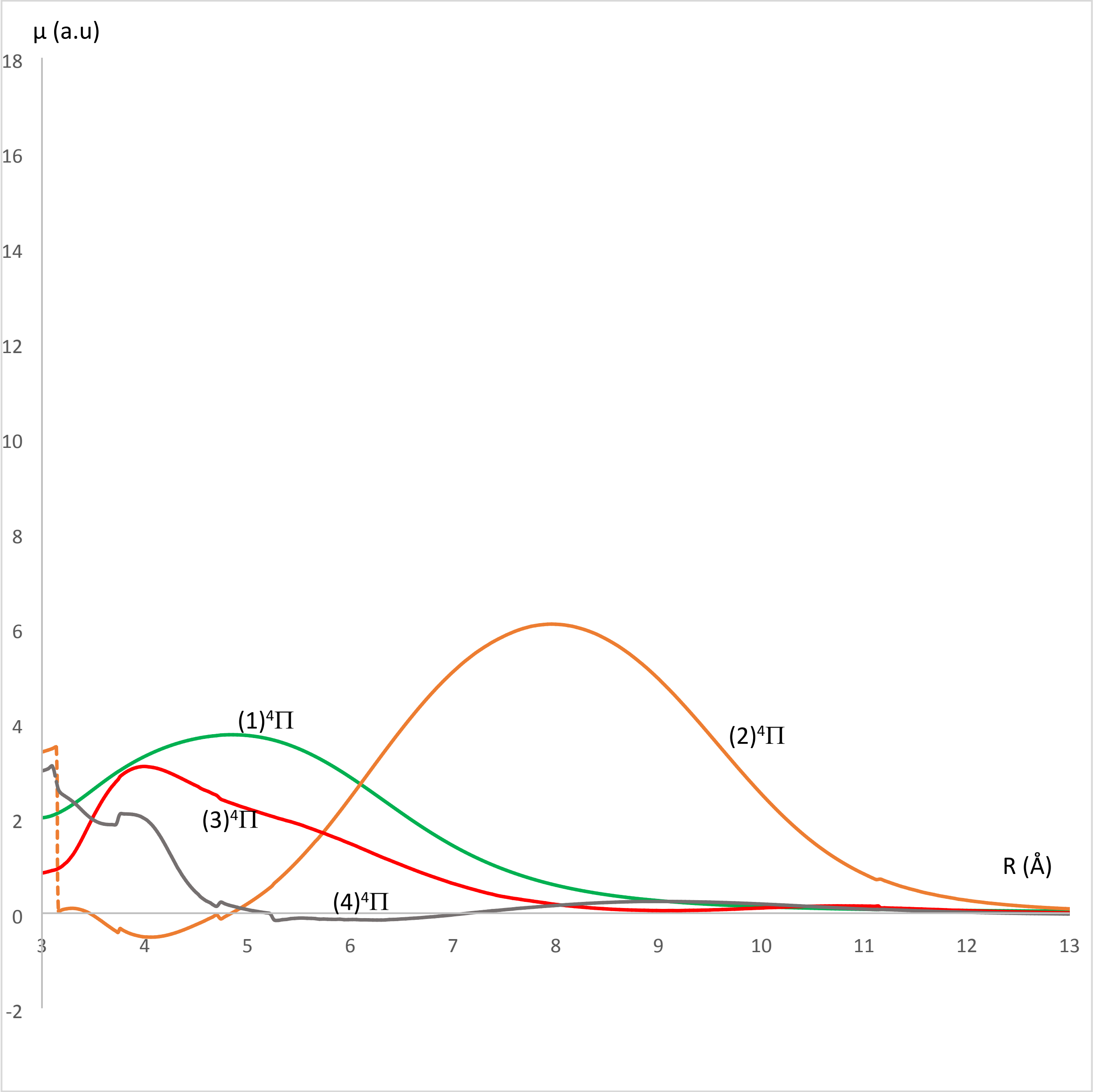

Figure S5: Dipole moment curves of the low-lying quartet states of symmetry two of CaCs molecule using CASSCF/MRCI method with 3 valence electrons.

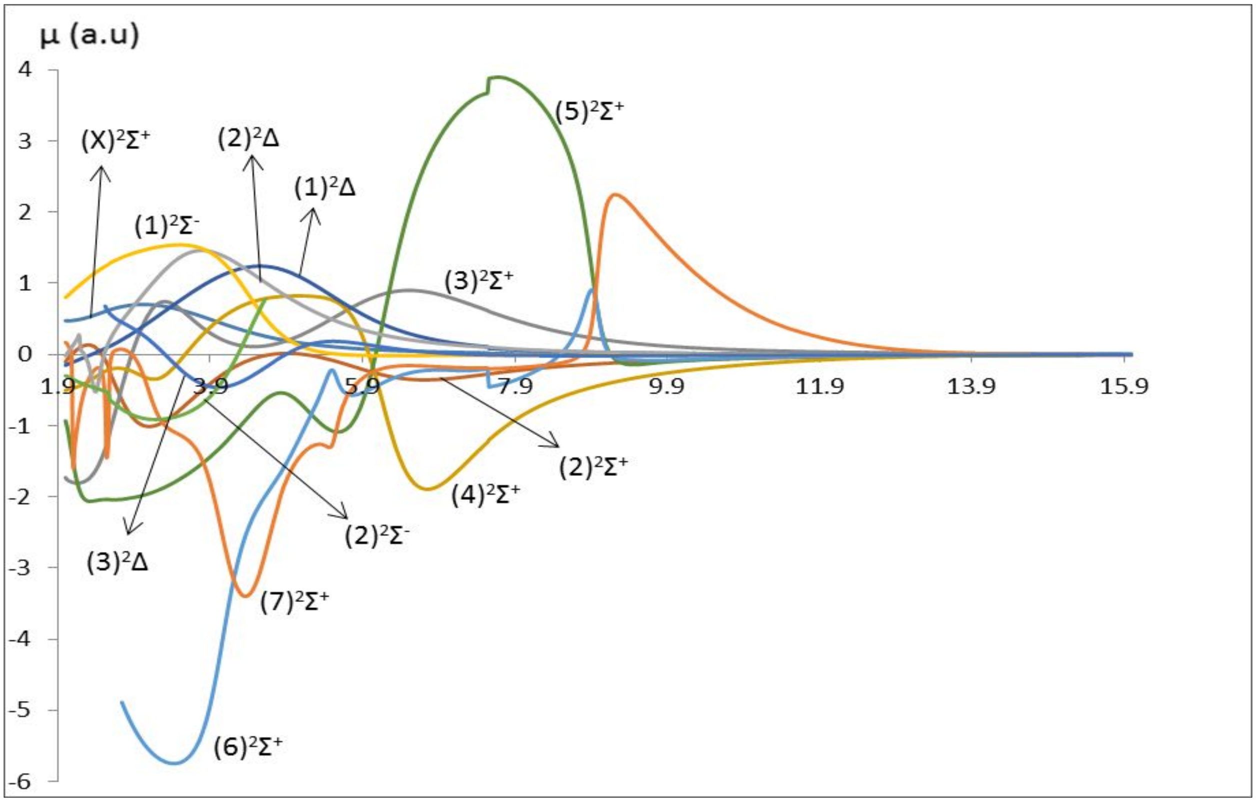

Figure S6: Dipole moment curves for the low-lying doublet states of symmetry one and four for CaNa molecule using CASSCF/MRCI method with 3 valence electrons.

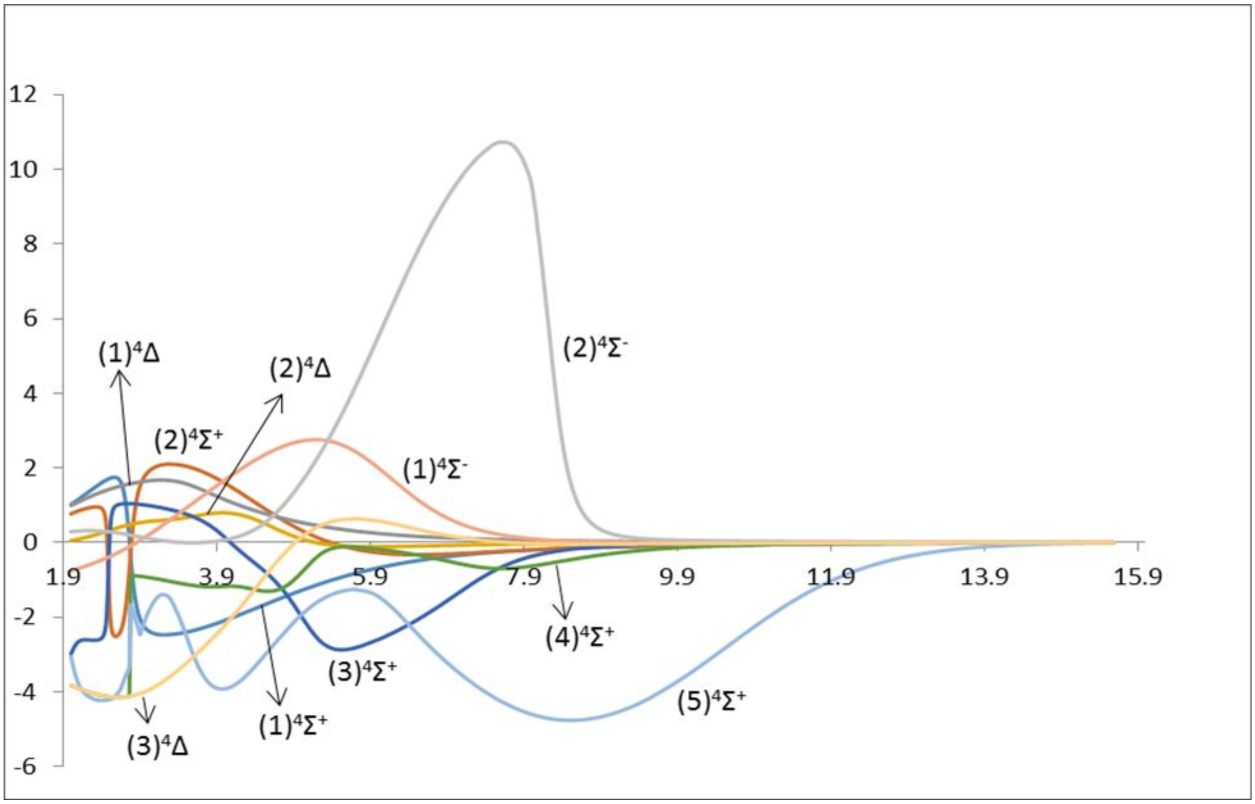

Figure S7: Dipole moment curves for the low-lying quartet states of symmetry one and four for CaNa molecule using CASSCF/MRCI method with 3 valence electrons.

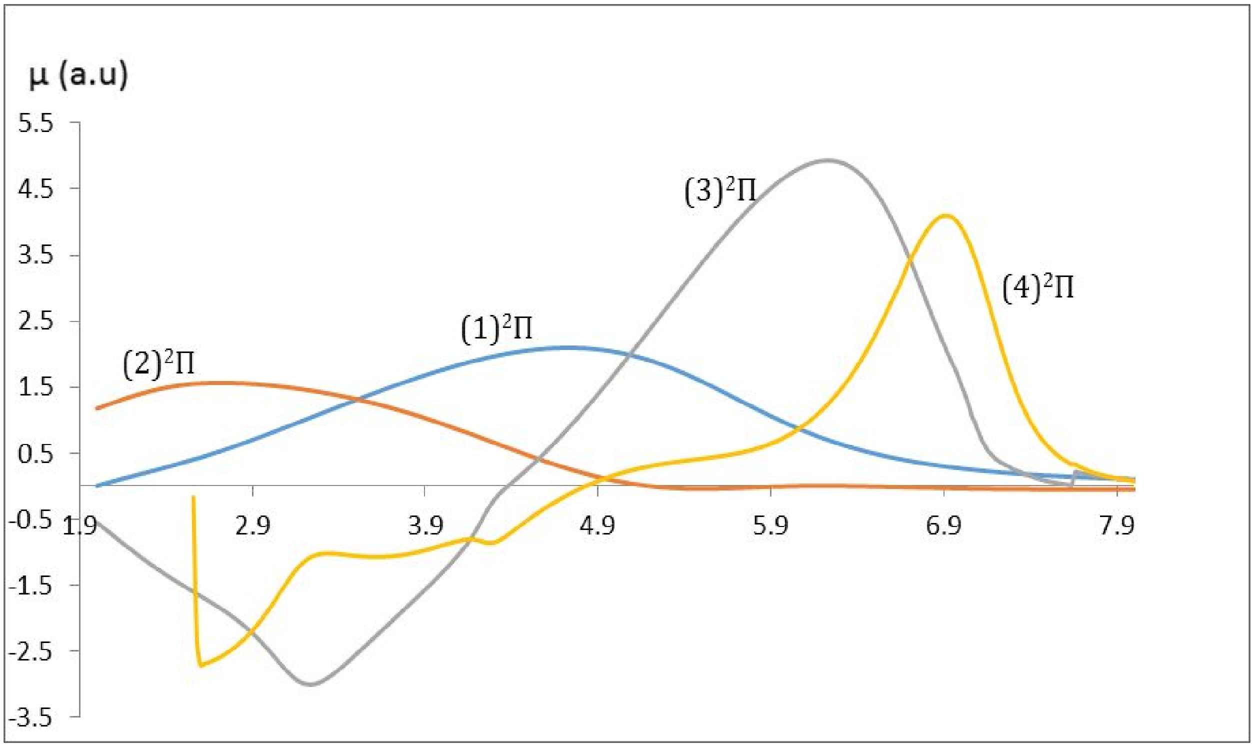

Figure S8: Dipole moment curves for the low-lying doublet states of symmetry two for CaNa molecule using CASSCF/MRCI method with 3 valence electrons.

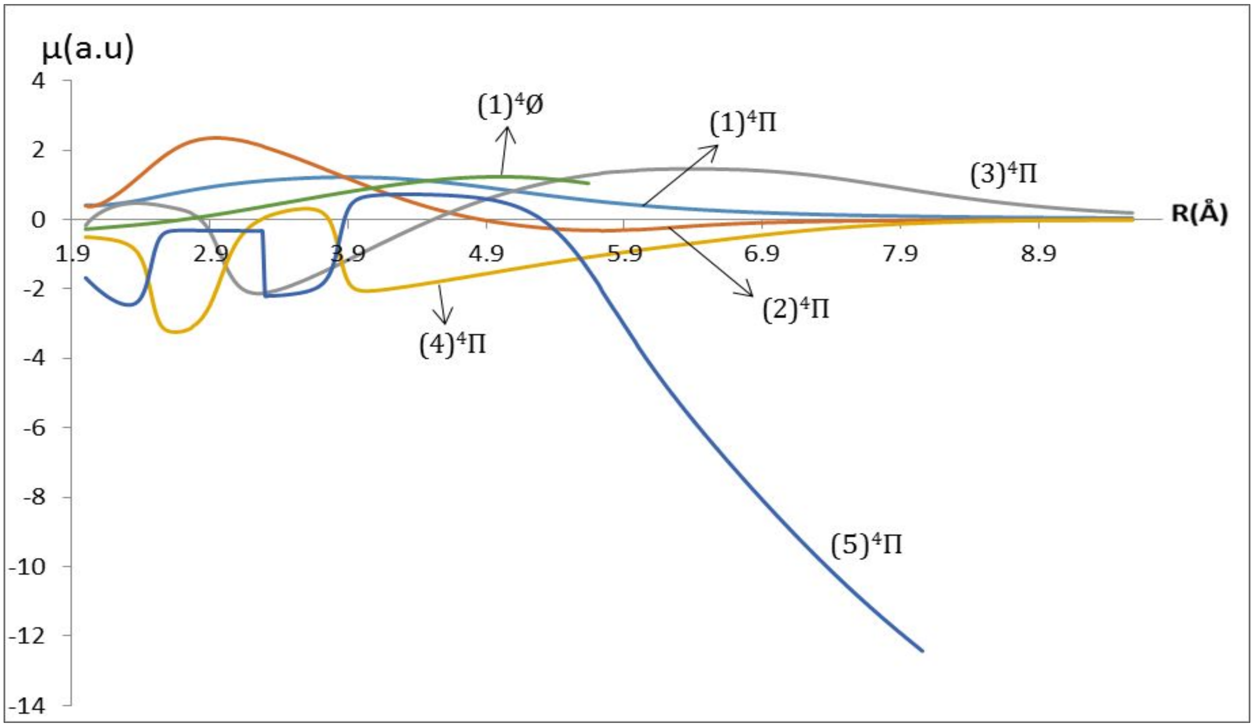

Figure S9: Dipole moment curves for the low-lying quartet states of symmetry two for CaNa molecule using CASSCF/MRCI method with 3 valence electrons.

Table S1: Position of the crossings R_c and avoided crossing R_{AC} with the energy difference ΔE of the molecules CaCs and CaNa.

CaCs					CaNa				
Crossing		Avoided crossing			crossing		Avoided crossing		
State1/ State2	R_c(Å)	State1/ State2	R_{ac} (Å)	ΔE(cm^{-1})	State1/ State2	R_c(Å)	State1/ State2	R_{ac} (Å)	ΔE(cm^{-1})
$(3)^2\Sigma^+/(1)^2\Delta$	3.74	$(3)^2\Sigma^+/(4)^2\Sigma^+$	5.24	1529.7162	$(2)^2\Sigma^+/(1)^2\Delta$	2.08	$(4)^2\Sigma^+/(5)^2\Sigma^+$	6.04	572.57
$(4)^2\Sigma^+/(1)^2\Delta$	4.26	$(2)^2\Pi/(3)^2\Pi$	7.68	2107.878	$(4)^2\Sigma^+/(1)^2\Delta$	3.18	$(6)^2\Sigma^+/(7)^2\Sigma^+$	5.72<br>8.96	51.20<br>17.77
$(5)^2\Sigma^+/(1)^2\Delta$	5.14	$(1)^4\Delta/(2)^4\Delta$	70	2533.790	$(1)^2\Delta/(5)^2\Sigma^+$	4.86	$(3)^2\Pi/(4)^2\Pi$	2.90	757.03
$(5)^2\Sigma^+/(2)^2\Delta$	3.32				$(5)^2\Sigma^+/(2)^2\Delta$	2.32	$(1)^4\Sigma^+/(2)^4\Sigma^+$	2.78	1079.31
$(1)^2\Sigma^+/(1)^2\Pi$	3.28				$(6)^2\Sigma^+/(2)^2\Delta$	2.92	$(2)^4\Sigma^+/(3)^4\Sigma^+$	2.50	510.67
$(2)^2\Sigma^+/(1)^2\Pi$	5.96				$(7)^2\Sigma^+/(2)^2\Delta$	3.62<br>9.22	$(2)^4\Pi/(3)^4\Pi$	4.64	3067.46
$(3)^2\Sigma^+/(3)^2\Pi$	3.20				$(1)^2\Delta/(6)^2\Sigma^+$	5.82	$(3)^4\Pi/(4)^4\Pi$	3.02	939.41
$(4)^2\Sigma^+/(3)^2\Pi$	4.20<br>6.80<br>8.88				$(1)^2\Delta/(7)^2\Sigma^+$	5.90	$(4)^4\Pi/(5)^4\Pi$	2.46<br>3.84	867.29<br>539.60
$(4)^2\Sigma^+/(4)^2\Pi$	3.20				$(1)^2\Sigma^-/(3)^2\Delta$	4.0<br>5.38			
$(5)^2\Sigma^+/(4)^2\Pi$	3.80<br>7.68				$(5)^2\Sigma^+/(1)^2\Sigma^-$	2.48			
$(1)^2\Delta/(2)^2\Pi$	3.40				$(6)^2\Sigma^+/(1)^2\Sigma^-$	2.98			
$(1)^2\Delta/(3)^2\Pi$	4.42				$(7)^2\Sigma^+/(1)^2\Sigma^-$	3.36			
$(2)^2\Delta/(1)^2\Sigma^-$	4.02				$(7)^2\Sigma^+/(3)^2\Delta$	3.04			
$(1)^4\Sigma^+/(3)^4\Sigma^+$	3.48				$(2)^2\Delta/(1)^2\Sigma^-$	3.04			
$(2)^4\Sigma^+/(3)^4\Sigma^+$	3.80<br>8.04				$(1)^4\Sigma^+/(1)^4\Sigma^-$	3.38			
$(1)^4\Sigma^+/(1)^4\Delta$	4.72				$(1)^4\Sigma^+/(1)^4\Delta$	2.54			
$(3)^4\Sigma^+/(1)^4\Delta$	8.50				$(1)^4\Sigma^-/(2)^4\Sigma^+$	4.40			
$(1)^4\Sigma^+/(2)^4\Pi$	3.66				$(1)^4\Sigma^-/(1)^4\Delta$	4.92			
$(2)^4\Sigma^+/(3)^4\Pi$	4.22				$(2)^4\Sigma^+/(1)^4\Delta$	2.76			
$(2)^4\Delta/(3)^4\Delta$	4.54				$(2)^4\Sigma^+/(2)^4\Sigma^-$	2.62			
$(1)^4\Delta/(1)^4\Pi$	3.60				$(2)^4\Sigma^+/(2)^4\Delta$	2.14			
$(1)^4\Delta/(2)^4\Pi$	6.04				$(2)^4\Delta/(2)^4\Sigma^-$	3.20			
$(2)^4\Delta/(4)^4\Pi$	3.56				$(2)^4\Sigma^-/(4)^4\Sigma^+$	3.76			
$(2)^4\Delta/(5)^4\Pi$	4.86<br>9.58				$(2)^4\Sigma^-/(3)^4\Sigma^+$	3.22			
$(3)^4\Delta/(5)^4\Pi$	4.76				$(2)^4\Sigma^-/(5)^4\Sigma^+$	4.30			
$(2)^4\Pi/(4)^4\Pi$	10.66				$(3)^4\Sigma^+/(2)^4\Delta$	3.46<br>3.14<br>4.20			
$(3)^4\Pi/(4)^4\Pi$	10.00				$(5)^4\Sigma^+/(3)^4\Delta$	3.22			
$(3)^4\Pi/(5)^4\Pi$	3.68				$(2)^4\Pi/(1)^4\Phi$	2.26			
$(4)^4\Pi/(5)^4\Pi$	4.48				$(3)^4\Pi/(1)^4\Phi$	2.60			
					$(4)^4\Pi/(1)^4\Phi$	2.90			
					$(5)^4\Pi/(1)^4\Phi$	3.60			

Table S2: Values of the eigenvalues, the rotational constants and, and the abscissas of the turning points for the different vibrational levels of states $X^2\Sigma^+$, $(2)^2\Sigma^+$, $(4)^2\Sigma^+$, $(1)^2\Pi$, $(2)^2\Pi$, $(3)^2\Pi$, $(4)^2\Pi$, $(5)^2\Pi$, $(1)^2\Delta$, $(1)^4\Pi$, $(2)^4\Pi$, $(3)^4\Pi$, $(1)^4\Delta$, and $(1)^4\Sigma^-$ of CaCs molecule.

state	v	$E_v\ (cm^{-1})$	$B_v \times 10^2\ (cm^{-1})$	$D_v \times 10^8\ (cm^{-1})$	R_{min} (Å)	R_{max} (Å)
$X^2\Sigma^+$	0	20.5	2.198	2.596	4.825	5.153
	1	60.6	2.172	2.680	4.726	5.301
	2	99.7	2.147	2.729	4.665	5.414
	3	137.9	2.121	2.793	4.618	5.512
	4	175.2	2.095	2.833	4.579	5.605
	5	211.6	2.069	2.979	4.546	5.688
	6	247.1	2.043	3.060	4.518	5.775
	7	281.7	2.014	3.392	4.492	5.860
	8	315.0	1.985	3.323	4.469	5.943
	9	347.4	1.957	3.406	4.448	6.026
	10	409.2	1.898	3.645	4.412	6.193
	11	438.7	1.867	3.835	4.396	6.278
	12	467.1	1.838	3.941	4.381	6.363
	13	521.2	1.773	4.447	4.355	6.542
	14	546.7	1.741	4.427	4.343	6.632
	15	571.2	1.708	4.728	4.332	6.727
	16	594.8	1.675	4.829	4.322	6.821
	17	617.4	1.639	4.972	4.313	6.923
	18	639.0	1.604	5.558	4.304	7.026
	19	659.7	1.569	5.837	4.295	7.132
	20	679.3	1.532	5.203	4.288	7.243
	21	698.1	1.495	6.367	4.280	7.362
state	v	$E_v\ (cm^{-1})$	$B_v \times 10^2\ (cm^{-1})$	$D_v \times 10^8\ (cm^{-1})$	R_{min} (Å)	R_{max} (Å)
$(2)^2\Sigma^+$	0	29.6	2.280	1.384	4.763	5.039
	1	88.0	2.272	1.376	4.674	5.148
	2	146.2	2.262	1.393	4.614	5.229
	3	204.0	2.253	1.364	4.568	5.295
	4	261.5	2.244	1.392	4.528	5.356
	5	318.7	2.235	1.430	4.494	5.411
	6	375.4	2.226	1.353	4.463	5.462
	7	432.0	2.219	1.480	4.435	5.511
	8	543.9	2.199	1.472	4.385	5.604
	9	599.3	2.191	1.402	4.364	5.647
	10	654.3	2.180	1.373	4.343	5.688
	11	709.2	2.174	1.390	4.324	5.731
	12	763.6	2.161	1.402	4.305	5.771
	13	817.8	2.154	1.591	4.288	5.811
	14	871.3	2.142	1.288	4.271	5.851
	15	924.7	2.134	1.516	4.255	5.889
	16	977.6	2.123	1.559	4.240	5.929
	17	1030.0	2.112	1.330	4.225	5.967
	18	1082.2	2.104	1.396	4.212	6.004

19	1134.0	2.093	1.600	4.198	6.041
20	1185.3	2.085	1.348	4.185	6.079
21	1236.4	2.072	1.546	4.172	6.116
22	1287.0	2.063	1.585	4.160	6.152
23	1337.1	2.053	1.419	4.149	6.189
24	1387.0	2.043	1.698	4.138	6.227
25	1436.2	2.031	1.443	4.127	6.265
26	1485.1	2.021	1.494	4.116	6.300
27	1533.6	2.009	1.565	4.106	6.337
28	1581.7	1.999	1.636	4.096	6.373
29	1629.3	1.991	1.510	4.086	6.410
30	1723.4	1.966	1.669	4.068	6.484
31	1769.8	1.956	1.682	4.059	6.521
32	1815.7	1.943	1.587	4.050	6.558
33	1861.2	1.931	1.555	4.042	6.596
34	1906.2	1.920	1.782	4.033	6.633
35	1950.8	1.909	1.566	4.025	6.670
36	2038.7	1.885	1.748	4.010	6.747
37	2081.9	1.873	1.843	4.002	6.785
38	2124.6	1.859	1.497	3.995	6.824
39	2166.9	1.847	1.719	3.988	6.863
40	2208.7	1.836	2.189	3.981	6.902
41	2250.0	1.823	1.401	3.975	6.942
42	2290.9	1.809	2.090	3.968	6.982
43	2331.1	1.796	1.812	3.962	7.023
44	2370.9	1.783	1.698	3.955	7.064
45	2410.3	1.769	1.926	3.949	7.105
46	2449.1	1.758	1.974	3.943	7.148
47	2487.4	1.740	1.574	3.938	7.190
48	2525.2	1.729	2.433	3.932	7.233
49	2562.5	1.714	1.708	3.926	7.277
50	2599.3	1.701	2.351	3.921	7.321
51	2635.4	1.685	1.548	3.916	7.365
52	2671.2	1.671	2.451	3.910	7.411
53	2706.3	1.658	1.845	3.905	7.458
54	2741.0	1.640	2.164	3.901	7.504
55	2775.1	1.628	2.005	3.896	7.551
56	2841.9	1.599	2.263	3.886	7.646
57	2874.6	1.586	2.212	3.882	7.694
58	2906.8	1.573	1.993	3.877	7.741
59	2938.6	1.558	1.921	3.873	7.791
60	3000.7	1.530	2.093	3.865	7.890
61	3031.0	1.515	2.045	3.861	7.941
62	3061.0	1.502	2.870	3.857	7.992
63	3090.3	1.487	1.809	3.853	8.043
64	3119.2	1.472	1.728	3.849	8.097

65	3175.7	1.443	2.347	3.842	8.206
66	3203.1	1.427	1.870	3.839	8.260
67	3230.1	1.411	2.319	3.835	8.317
68	3256.6	1.396	2.877	3.832	8.374
69	3282.6	1.380	2.275	3.829	8.433
70	3308.1	1.364	1.807	3.825	8.493
71	3333.2	1.347	2.911	3.822	8.553
72	3357.7	1.333	2.722	3.819	8.615
73	3381.8	1.317	2.662	3.816	8.678
74	3405.3	1.300	2.412	3.813	8.742
75	3428.4	1.284	2.756	3.811	8.807
76	3451.0	1.267	2.272	3.808	8.874
77	3473.1	1.248	2.936	3.805	8.943

state	v	$E_v\ (cm^{-1})$	$B_v \times 10^2$ (cm^{-1})	$D_v \times 10^8$ (cm^{-1})	R_{min} (Å)	R_{max} (Å)
$(4)^2\Sigma^+$	0	38.9	2.366	0.883	4.690	4.929
	1	115.9	2.348	0.885	4.619	5.031
	2	192.0	2.331	0.955	4.571	5.106
	3	266.4	2.316	1.027	4.532	5.173
	4	339.0	2.304	1.079	4.499	5.235
	5	409.9	2.290	1.051	4.469	5.288
	6	479.9	2.280	1.152	4.443	5.343
	7	548.3	2.261	1.164	4.420	5.391
	8	615.4	2.251	1.113	4.397	5.439
	9	681.8	2.241	1.071	4.376	5.484
	10	747.7	2.230	1.268	4.357	5.528
	11	812.3	2.213	1.117	4.339	5.572
	12	876.3	2.204	1.488	4.323	5.622
	13	938.6	2.184	1.140	4.307	5.664
	14	1000.3	2.174	1.358	4.292	5.703
	15	1061.1	2.159	1.196	4.278	5.747
	16	1121.0	2.142	1.966	4.261	5.793
	17	1179.0	2.124	1.540	4.248	5.839
	18	1291.5	2.092	1.951	4.223	5.929
	19	1345.6	2.072	1.515	4.212	5.975
	20	1398.8	2.058	1.824	4.201	6.026
	21	1450.6	2.029	1.722	4.190	6.075
	22	1501.2	2.012	2.034	4.180	6.126
	23	1550.4	1.986	2.038	4.171	6.074
	24	1598.2	1.963	2.218	4.161	6.230
	25	1644.5	1.936	2.920	4.152	6.288
	26	1688.7	1.906	1.703	4.144	6.347
	27	1731.9	1.881	3.519	4.136	6.410
	28	1773.0	1.853	3.093	4.129	6.475

state	v	$E_v\ (cm^{-1})$	$B_v \times 10^2$ (cm^{-1})	$D_v \times 10^8$ (cm^{-1})	R_{min} (Å)	R_{max} (Å)
	29	1812.3	1.814	3.139	4.122	6.547
	30	1849.7	1.783	3.494	4.115	6.616
	31	1885.3	1.745	3.966	4.109	6.694
	32	1919.0	1.709	3.319	4.103	6.784
	33	1950.7	1.653	5.480	4.097	6.875
state	v	$E_v\ (cm^{-1})$	$B_v \times 10^2$ (cm^{-1})	$D_v \times 10^8$ (cm^{-1})	R_{min} (Å)	R_{max} (Å)
$(1)^2\Pi$	0	41.9	3.024	1.581	4.141	4.370
	1	125.4	3.016	1.597	4.064	4.461
	2	208.4	3.007	1.580	4.013	4.526
	3	291.2	2.998	1.583	3.972	4.581
	4	373.6	2.989	1.607	3.938	4.630
	5	455.6	2.981	1.587	3.909	4.674
	6	537.3	2.972	1.595	3.882	4.716
	7	618.6	2.963	1.625	3.857	4.755
	8	699.5	2.953	1.592	3.835	4.792
	9	780.1	2.945	1.616	3.814	4.828
	10	860.3	2.936	1.588	3.795	4.863
	11	940.2	2.926	1.607	3.777	4.896
	12	1019.7	2.918	1.613	3.759	4.929
	13	1098.9	2.908	1.647	3.743	4.961
	14	1177.6	2.899	1.603	3.727	4.992
	15	1256.1	2.890	1.624	3.712	5.022
	16	1334.1	2.881	1.620	3.698	5.052
	17	1411.8	2.872	1.603	3.684	5.081
	18	1489.2	2.863	1.640	3.671	5.110
	19	1566.3	2.854	1.669	3.658	5.138
	20	1642.9	2.846	1.576	3.646	5.166
	21	1719.3	2.837	1.667	3.634	5.194
	22	1795.2	2.827	1.668	3.623	5.221
	23	1870.8	2.818	1.591	3.611	5.249
	24	1946.0	2.810	1.684	3.601	5.275
	25	2020.9	2.800	1.632	3.590	5.302
	26	2095.5	2.792	1.646	3.580	5.328
	27	2169.7	2.782	1.710	3.570	5.355
	28	2243.5	2.773	1.589	3.560	5.381
	29	2390.1	2.755	1.645	3.541	5.432
	30	2462.9	2.746	1.688	3.532	5.458
	31	2535.3	2.736	1.663	3.524	5.483
	32	2607.4	2.727	1.666	3.515	5.509
	33	2679.1	2.718	1.676	3.507	5.534
	34	2750.4	2.709	1.703	3.498	5.559
	35	2821.4	2.699	1.670	3.490	5.584

36	2892.1	2.690	1.651	3.483	5.609
37	2962.4	2.681	1.724	3.475	5.634
38	3032.3	2.672	1.766	3.467	5.659
39	3101.8	2.662	1.633	3.460	5.684
40	3171.0	2.652	1.772	3.453	5.708
41	3239.8	2.643	1.637	3.445	5.733
42	3308.3	2.633	1.823	3.438	5.758
43	3376.3	2.624	1.657	3.432	5.783
44	3444.0	2.614	1.727	3.425	5.808
45	3511.4	2.605	1.802	3.418	5.832
46	3578.4	2.595	1.706	3.412	5.857
47	3645.0	2.585	1.799	3.405	5.882
48	3711.1	2.575	1.717	3.399	5.907
49	3777.0	2.566	1.745	3.393	5.931
50	3907.5	2.546	1.652	3.381	5.981
51	3972.2	2.535	1.928	3.375	6.006
52	4036.4	2.525	1.718	3.369	6.031
53	4100.3	2.514	1.850	3.364	6.056
54	4163.7	2.503	1.839	3.358	6.081
55	4226.8	2.494	1.785	3.353	6.107
56	4351.6	2.471	1.928	3.342	6.158
57	4413.3	2.460	1.755	3.337	6.184
58	4474.7	2.449	1.936	3.331	6.210
59	4535.6	2.437	1.956	3.326	6.236
60	4596.0	2.426	1.879	3.321	6.262
61	4656.0	2.414	2.038	3.317	6.289
62	4715.5	2.401	1.920	3.312	6.316
63	4774.5	2.391	1.967	3.307	6.343
64	4833.1	2.378	2.099	3.302	6.370
65	4891.1	2.364	2.135	3.298	6.398
66	4948.6	2.351	1.953	3.293	6.426
67	5005.7	2.340	2.100	3.289	6.454
68	5062.2	2.325	2.292	3.284	6.482
69	5118.1	2.311	1.987	3.280	6.511
70	5173.5	2.298	2.266	3.276	6.540
71	5228.4	2.283	2.339	3.272	6.570
72	5282.6	2.268	2.264	3.268	6.601
73	5336.2	2.252	2.438	3.264	6.631
74	5389.2	2.236	2.362	3.260	6.663
75	5493.2	2.203	2.480	3.252	6.728
76	5544.2	2.185	2.725	3.249	6.761
77	5594.4	2.166	2.604	3.245	6.796
78	5643.9	2.148	2.740	3.241	6.831

state	v	$E_v\ (cm^{-1})$	$B_v \times 10^2\ (cm^{-1})$	$D_v \times 10^8\ (cm^{-1})$	R_{min} (Å)	R_{max} (Å)
	79	5692.7	2.129	2.710	3.238	6.866
	80	5740.7	2.109	3.089	3.235	6.904
	81	5787.8	2.087	2.890	3.231	6.942
	82	5834.1	2.065	3.346	3.228	6.982
	83	5879.5	2.042	3.111	3.225	7.022
	84	5923.9	2.021	3.104	3.222	7.064
	85	5967.6	1.995	3.830	3.219	7.108
	86	6010.1	1.969	3.473	3.216	7.153
	87	6051.6	1.942	3.943	3.213	7.200
	88	6092.1	1.915	3.719	3.210	7.251
	89	6131.4	1.885	4.313	3.208	7.301
	90	6169.6	1.854	4.273	3.205	7.357
	91	6206.7	1.824	4.211	3.203	7.413
	92	6242.6	1.792	4.600	3.200	7.473
	93	6277.3	1.758	4.912	3.198	7.535
	94	6310.7	1.726	4.682	3.196	7.600
	95	6343.0	1.688	5.716	3.193	7.668
	96	6431.1	1.567	5.712	3.188	7.906
	97	6457.7	1.522	7.029	3.186	7.996
	98	6482.7	1.480	6.798	3.184	8.093
	99	6506.3	1.432	6.956	3.183	8.195
	100	6528.5	1.387	7.630	3.181	8.307
state	v	$E_v\ (cm^{-1})$	$B_v \times 10^2\ (cm^{-1})$	$D_v \times 10^8\ (cm^{-1})$	R_{min} (Å)	R_{max} (Å)
$(2)^2\Pi$	0	20.7	2.193	2.482	4.833	5.156
	1	61.7	2.176	2.496	4.731	5.297
	2	102.2	2.157	2.498	4.666	5.401
	3	142.1	2.138	2.479	4.616	5.491
	4	181.6	2.122	2.398	4.575	5.571
	5	220.9	2.105	2.489	4.539	5.646
	6	259.7	2.089	2.321	4.507	5.715
	7	298.2	2.072	2.441	4.479	5.781
	8	336.4	2.059	2.206	4.453	5.844
	9	412.2	2.033	2.214	4.407	5.961
	10	449.8	2.019	1.999	4.387	6.015
	11	487.4	2.010	2.245	4.367	6.067
	12	524.8	1.997	1.904	4.349	6.119
	13	562.2	1.988	2.051	4.331	6.168
	14	599.4	1.978	1.839	4.314	6.216
	15	636.7	1.968	1.860	4.298	6.261
	16	673.9	1.961	1.576	4.282	6.305
	17	748.6	1.946	1.578	4.253	6.389
	18	785.9	1.940	1.769	4.240	6.430

19	823.3	1.934	1.361	4.226	6.468
20	860.8	1.928	1.633	4.213	6.505
21	898.4	1.923	1.314	4.200	6.541
22	936.2	1.917	1.608	4.188	6.578
23	973.9	1.912	1.442	4.176	6.612
24	1011.7	1.908	1.448	4.164	6.646
25	1049.6	1.902	1.271	4.153	6.679
26	1087.6	1.897	1.463	4.142	6.712
27	1125.7	1.895	1.369	4.131	6.744
28	1163.8	1.889	1.393	4.120	6.775
29	1202.0	1.884	1.172	4.110	6.806
30	1240.3	1.882	1.395	4.100	6.835
31	1278.7	1.876	1.143	4.090	6.866
32	1317.2	1.875	1.223	4.080	6.895
33	1355.7	1.869	1.487	4.070	6.923
34	1394.2	1.866	1.101	4.061	6.952
35	1432.9	1.861	1.360	4.051	6.981
36	1471.5	1.858	1.169	4.042	7.008
37	1510.3	1.854	1.197	4.033	7.035
38	1549.0	1.851	1.043	4.024	7.063
39	1587.9	1.847	1.447	4.016	7.090
40	1626.7	1.842	1.146	4.007	7.117
41	1665.6	1.840	1.375	3.999	7.143
42	1704.5	1.834	1.183	3.991	7.170
43	1743.3	1.829	1.241	3.982	7.196
44	1782.2	1.827	1.064	3.974	7.222
45	1821.1	1.823	1.283	3.967	7.249
46	1860.0	1.817	1.223	3.959	7.275
47	1898.8	1.811	1.437	3.951	7.301
48	1937.6	1.808	0.996	3.944	7.327
49	1976.4	1.806	1.370	3.936	7.353
50	2015.1	1.799	1.168	3.929	7.379
51	2053.8	1.794	1.502	3.922	7.406
52	2092.4	1.791	1.135	3.915	7.432
53	2131.0	1.785	1.206	3.908	7.457
54	2169.6	1.779	1.327	3.901	7.484
55	2208.0	1.775	1.125	3.895	7.510
56	2246.4	1.769	1.573	3.888	7.536
57	2284.6	1.764	1.274	3.881	7.562
58	2322.8	1.757	1.342	3.875	7.590
59	2360.8	1.750	1.412	3.869	7.617
60	2398.6	1.743	1.571	3.862	7.645
61	2436.3	1.737	1.483	3.856	7.672

62	2473.8	1.731	1.446	3.850	7.701
63	2511.2	1.722	1.532	3.844	7.730
64	2585.2	1.707	1.573	3.833	7.788
65	2621.9	1.700	1.952	3.827	7.818
66	2658.4	1.691	1.187	3.821	7.848
67	2730.6	1.674	1.662	3.811	7.910
68	2766.3	1.662	2.003	3.805	7.942
69	2801.7	1.654	1.331	3.800	7.974
70	2871.6	1.632	2.225	3.790	8.043
71	2906.0	1.623	1.736	3.785	8.077
72	2940.0	1.607	2.054	3.780	8.114
73	2973.6	1.597	2.397	3.775	8.152
74	3006.8	1.583	1.967	3.771	8.190
75	3039.5	1.567	2.865	3.766	8.230
76	3071.6	1.553	2.250	3.762	8.272
77	3103.3	1.537	2.612	3.758	8.316
78	3134.3	1.519	2.650	3.753	8.361
79	3164.8	1.499	3.140	3.749	8.409
80	3194.5	1.480	3.297	3.745	8.461
81	3223.5	1.459	3.221	3.741	8.515
82	3251.8	1.434	3.920	3.738	8.572
83	3305.6	1.382	4.830	3.731	8.699
84	3331.0	1.351	4.544	3.727	8.772
85	3355.3	1.318	5.386	3.724	8.848
86	3378.5	1.281	5.624	3.721	8.937
87	3400.4	1.244	6.108	3.719	9.032
88	3420.9	1.197	6.669	3.716	9.138

state	v	$E_v\ (cm^{-1})$	$B_v \times 10^2$ (cm^{-1})	$D_v \times 10^8$ (cm^{-1})	R_{min} (Å)	R_{max} (Å)
$(3)^2\Pi$	0	33.7	2.681	1.712	4.391	4.646
	1	100.6	2.667	1.732	4.309	4.753
	2	166.8	2.652	1.785	4.254	4.830
	3	232.0	2.638	1.809	4.212	4.897
	4	296.5	2.621	1.908	4.176	4.958
	5	359.8	2.603	1.927	4.146	5.016
	6	422.0	2.584	2.084	4.118	5.071
	7	483.0	2.565	2.090	4.094	5.125
	8	542.7	2.542	2.289	4.072	5.179
	9	601.0	2.518	2.490	4.052	5.234
	10	657.5	2.490	2.656	4.033	5.290
	11	712.1	2.456	3.080	4.017	5.349
	12	764.4	2.418	3.632	4.001	5.413
	13	813.7	2.367	4.381	4.987	5.484
	14	859.5	2.302	6.691	3.975	5.567

	15	899.5	2.173	16.066	3.964	5.684

state	v	$E_v\ (cm^{-1})$	$B_v \times 10^2$ (cm^{-1})	$D_v \times 10^8$ (cm^{-1})	R_{min} (Å)	R_{max} (Å)
$(4)^2\Pi$	0	28.4	2.028	1.049	5.060	5.338
	1	141.0	2.046	1.129	5.878	5.503
	2	196.9	2.055	1.186	4.817	5.558
	3	252.4	2.063	1.208	4.763	5.605
	4	307.8	2.071	1.217	4.716	5.648
	5	363.1	2.077	1.244	4.672	5.686
	6	418.2	2.084	1.302	4.632	5.722
	7	473.2	2.089	1.316	4.594	5.757
	8	528.0	2.095	1.330	4.560	5.790
	9	582.6	2.098	1.373	4.527	5.822
	10	637.1	2.101	1.367	4.496	5.852
	11	691.4	2.107	1.362	4.466	5.882
	12	745.7	2.109	1.426	4.438	5.911
	13	799.8	2.112	1.450	4.411	5.934
	14	853.7	2.114	1.340	4.385	5.967
	15	907.6	2.116	1.491	4.360	5.994
	16	961.4	2.119	1.452	4.337	6.021
	17	1015.0	2.120	1.442	4.314	6.047
	18	1068.5	2.121	1.512	4.292	6.073
	19	1121.9	2.122	1.427	4.271	6.098
	20	1175.2	2.121	1.479	4.251	6.125
	21	1228.4	2.123	1.498	4.231	6.149
	22	1281.5	2.121	1.491	4.212	6.174
	23	1334.4	2.122	1.519	4.194	6.199
	24	1387.3	2.122	1.415	4.176	6.223
	25	1440.1	2.121	1.577	4.159	6.248
	26	1492.7	2.120	1.546	4.142	6.272
	27	1545.2	2.120	1.442	4.125	6.296
	28	1597.6	2.117	1.630	4.109	6.319
	29	1649.9	2.117	1.360	4.094	6.343
	30	1702.1	2.115	1.558	4.079	6.367
	31	1754.2	2.113	1.640	4.064	6.390
	32	1806.1	2.111	1.519	4.050	6.414
	33	1857.9	2.109	1.379	4.036	6.437
	34	1909.6	2.107	1.561	4.022	6.460
	35	1961.3	2.105	1.621	4.009	6.483
	36	2012.7	2.102	1.394	3.996	6.506
	37	2064.1	2.101	1.573	3.984	6.529
	38	2115.4	2.097	1.671	3.971	6.552
	39	2166.4	2.093	1.411	3.959	6.575

40	2217.4	2.091	1.565	3.947	6.598
41	2268.2	2.088	1.639	3.936	6.621
42	2318.8	2.085	1.459	3.925	6.644
43	2369.4	2.082	1.546	3.913	6.667
44	2419.9	2.079	1.598	3.903	6.690
45	2470.1	2.074	1.489	3.892	6.713
46	2520.3	2.071	1.660	3.881	6.736
47	2570.2	2.067	1.588	3.871	6.759
48	2620.0	2.063	1.568	3.861	6.781
49	2669.7	2.061	1.543	3.851	6.804
50	2719.3	2.057	1.676	3.841	6.827
51	2768.6	2.054	1.502	3.832	6.849
52	2817.9	2.049	1.635	3.822	6.873
53	2867.0	2.045	1.389	3.813	6.896
54	2915.9	2.040	1.652	3.804	6.918
55	2964.7	2.035	1.486	3.795	6.941
56	3013.3	2.032	1.479	3.787	6.964
57	3061.8	2.026	1.541	3.778	6.987
58	3110.2	2.022	1.509	3.770	7.010
59	3158.4	2.017	1.706	3.761	7.033
60	3206.4	2.012	1.589	3.753	7.057
61	3254.2	2.006	1.654	3.745	7.079
62	3301.8	2.003	1.427	3.738	7.102
63	3349.4	1.999	1.712	3.730	7.126
64	3396.7	1.993	1.498	3.722	7.149
65	3443.9	1.988	1.612	3.715	7.172
66	3490.9	1.983	1.648	3.707	7.195
67	3537.7	1.979	1.271	3.700	7.218
68	3584.5	1.973	1.610	3.693	7.242
69	3631.0	1.967	1.690	3.686	7.265
70	3677.3	1.962	1.686	3.679	7.289
71	3769.4	1.951	1.768	3.665	7.336
72	3815.1	1.946	1.492	3.659	7.360
73	3860.7	1.941	1.617	3.652	7.384
74	3906.1	1.934	1.562	3.646	7.408
75	3951.3	1.928	1.695	3.639	7.432
76	3996.3	1.923	1.416	3.633	7.457
77	4041.2	1.917	1.801	3.627	7.481
78	4085.8	1.910	1.621	3.621	7.506
79	4130.1	1.903	1.807	3.615	7.530
80	4174.3	1.898	1.577	3.609	7.555
81	4218.2	1.891	1.762	3.603	7.580
82	4261.9	1.884	1.642	3.597	7.605

83	4305.5	1.879	1.590	3.592	7.630
84	4348.8	1.872	1.857	3.586	7.656
85	4391.8	1.863	1.771	3.580	7.681
86	4434.6	1.858	1.202	3.575	7.707
87	4477.3	1.852	1.982	3.570	7.733
88	4519.7	1.844	1.666	3.564	7.759
89	4561.9	1.838	1.613	3.559	7.784
90	4603.8	1.832	1.618	3.554	7.811
91	4645.6	1.825	1.760	3.549	7.837
92	4687.2	1.818	1.687	3.544	7.863
93	4728.5	1.811	1.531	3.539	7.889
94	4769.7	1.805	1.662	3.534	7.914
95	4810.6	1.799	1.508	3.529	7.942
96	4851.4	1.794	1.750	3.524	7.967
97	4892.0	1.786	1.851	3.520	7.994
98	4932.3	1.777	1.874	3.515	8.024
99	4972.3	1.769	1.726	3.510	8.049
100	5012.0	1.761	1.434	3.506	8.075

state	v	$E_v\ (cm^{-1})$	$B_v \times 10^2\ (cm^{-1})$	$D_v \times 10^8\ (cm^{-1})$	R_{min} (Å)	R_{max} (Å)
$(5)^2\Pi$	0	27.5	2.313	1.637	4.724	5.005
	1	82.2	2.297	1.629	4.636	5.123
	2	136.5	2.283	1.682	4.577	5.212
	3	190.1	2.270	1.733	4.530	5.287
	4	242.9	2.255	1.829	4.491	5.355
	5	294.8	2.242	1.738	4.458	5.416
	6	396.7	2.214	2.019	4.400	5.536
	7	446.4	2.197	1.925	4.375	5.590
	8	495.3	2.185	2.042	4.351	5.645
	9	543.4	2.169	2.071	4.329	5.698
	10	590.7	2.154	2.093	4.309	5.751
	11	637.3	2.139	1.942	4.290	5.803
	12	683.4	2.125	2.388	4.272	5.855
	13	728.5	2.105	2.268	4.254	5.906
	14	772.8	2.091	2.291	4.238	5.958
	15	816.4	2.075	2.676	4.222	6.010
	16	859.0	2.058	2.193	4.207	6.062
	17	900.9	2.039	2.740	4.193	6.115
	18	942.0	2.022	2.700	4.179	6.168
	19	982.2	2.006	2.861	4.165	6.221
	20	1021.6	1.985	2.540	4.152	6.275
	21	1060.3	1.968	3.169	4.140	6.330
	22	1098.0	1.949	3.005	4.128	6.385
	23	1135.0	1.931	3.133	4.116	6.441
	24	1171.1	1.909	2.924	4.105	6.498

state	v	$E_v\ (cm^{-1})$	$B_v \times 10^2\ (cm^{-1})$	$D_v \times 10^8\ (cm^{-1})$	R_{min} (Å)	R_{max} (Å)
	25	1206.4	1.889	3.351	4.094	6.556
	26	1240.9	1.870	3.794	4.083	6.615
	27	1274.4	1.849	3.264	4.073	6.674
	28	1307.2	1.827	3.337	4.064	6.736
	29	1339.3	1.805	3.803	4.054	6.798
	30	1370.4	1.782	3.567	4.045	6.862
	31	1400.8	1.759	4.165	4.036	6.927
	32	1430.3	1.739	4.130	4.028	6.995
	33	1459.0	1.712	3.877	4.020	7.063
	34	1486.9	1.690	4.397	4.012	7.132
	35	1514.1	1.666	4.245	4.004	7.204
	36	1540.4	1.642	3.851	3.997	7.277
	37	1566.0	1.614	4.108	3.990	7.352
	38	1590.8	1.593	4.911	3.983	7.429
	39	1615.0	1.572	3.949	3.977	7.503
	40	1638.5	1.549	3.154	3.970	7.580
	41	1661.6	1.532	3.918	3.964	7.653
	42	1684.3	1.512	4.415	3.958	7.727
	43	1748.8	1.445	5.151	3.941	7.962
	44	1769.1	1.424	4.339	3.936	8.048
	45	1788.8	1.397	5.100	3.931	8.135
	46	1807.8	1.370	5.106	3.926	8.227
	47	1826.1	1.345	5.342	3.922	8.315
	48	1877.6	1.271	6.122	3.909	8.609
	49	1893.5	1.242	4.943	3.905	8.714
	50	1908.9	1.220	6.282	3.901	8.817
	51	1923.7	1.197	3.358	3.897	8.931
	52	1938.3	1.181	6.742	3.894	9.028
	53	1952.2	1.154	4.030	3.894	9.148

state	v	$E_v\ (cm^{-1})$	$B_v \times 10^2\ (cm^{-1})$	$D_v \times 10^8\ (cm^{-1})$	R_{min} (Å)	R_{max} (Å)
$(1)^2\Delta$	0	40.5	3.102	1.821	4.086	4.318
	1	121.4	3.094	1.825	4.007	4.410
	2	201.9	3.086	1.812	3.955	4.476
	3	282.2	3.077	1.807	3.914	4.532
	4	362.3	3.068	1.782	3.879	4.581
	5	442.2	3.059	1.788	3.850	4.625
	6	521.9	3.049	1.793	3.822	4.667
	7	601.3	3.041	1.839	3.797	4.706
	8	680.3	3.033	1.898	3.774	4.744
	9	758.8	3.026	1.861	3.753	4.780
	10	837.0	3.017	1.791	3.733	4.814
	11	915.0	3.008	1.801	3.714	4.848
	12	992.6	2.999	1.849	3.696	4.880
	13	1070.0	2.992	1.904	3.679	4.912

14	1146.9	2.984	1.856	3.663	4.943
15	1223.5	2.975	1.806	3.648	4.973
16	1299.9	2.966	1.813	3.633	5.002
17	1376.0	2.958	1.925	3.619	5.031
18	1451.7	2.950	1.907	3.605	5.060
19	1527.0	2.942	1.798	3.592	5.088
20	1602.1	2.933	1.835	3.579	5.116
21	1676.9	2.925	1.906	3.566	5.143
22	1751.3	2.917	1.893	3.554	5.170
23	1825.5	2.909	1.835	3.543	5.197
24	1899.3	2.900	1.848	3.531	5.223
25	1972.9	2.892	1.890	3.521	5.250
26	2046.2	2.884	1.922	3.510	5.276
27	2119.1	2.876	1.842	3.499	5.301
28	2191.8	2.867	1.832	3.489	5.327
29	2264.2	2.859	1.885	3.479	5.352
30	2336.3	2.851	1.942	3.470	5.377
31	2408.0	2.843	1.869	3.460	5.402
32	2479.5	2.835	1.829	3.451	5.426
33	2550.7	2.826	1.898	3.442	5.451
34	2621.7	2.819	1.945	3.433	5.475
35	2692.3	2.811	1.881	3.424	5.500
36	2762.6	2.802	1.831	3.416	5.524
37	2832.7	2.794	1.882	3.408	5.548
38	2902.4	2.786	1.946	3.400	5.572
39	2971.9	2.778	1.901	3.392	5.596
40	3041.1	2.770	1.828	3.384	5.619
41	3110.0	2.762	1.868	3.376	5.643
42	3178.7	2.754	1.917	3.368	5.666
43	3247.1	2.747	1.874	3.361	5.689
44	3315.2	2.739	1.863	3.354	5.713
45	3383.1	2.730	1.866	3.347	5.736
46	3450.7	2.722	1.959	3.340	5.759
47	3517.9	2.714	1.945	3.333	5.782
48	3584.9	2.706	1.847	3.326	5.805
49	3651.7	2.698	1.876	3.319	5.828
50	3718.1	2.690	1.865	3.312	5.851
51	3784.3	2.682	1.986	3.306	5.874
52	3850.2	2.673	1.869	3.300	5.897
53	3981.2	2.657	1.882	3.287	5.942
54	4046.3	2.649	1.945	3.281	5.965
55	4111.1	2.641	1.898	3.275	5.988
56	4175.6	2.633	1.855	3.269	6.011

57	4239.9	2.625	1.878	3.263	6.033
58	4303.9	2.616	1.924	3.257	6.056
59	4367.7	2.609	1.882	3.252	6.079
60	4431.1	2.601	1.916	3.246	6.101
61	4494.3	2.592	1.883	3.241	6.124
62	4557.3	2.584	1.852	3.235	6.146
63	4620.0	2.576	1.921	3.230	6.169
64	4682.4	2.568	1.928	3.225	6.191
65	4744.6	2.560	1.843	3.219	6.214
66	4806.5	2.552	1.929	3.214	6.236
67	4868.1	2.544	1.865	3.209	6.259
68	4929.4	2.536	1.924	3.204	6.281
69	4990.6	2.528	1.884	3.199	6.304
70	5051.4	2.520	1.826	3.194	6.327
71	5112.0	2.512	1.994	3.189	6.349
72	5172.3	2.503	1.871	3.185	6.372
73	5232.4	2.495	1.890	3.180	6.394
74	5292.2	2.487	1.871	3.175	6.417
75	5351.7	2.479	1.889	3.171	6.439
76	5411.0	2.471	1.836	3.166	6.462
77	5470.0	2.463	1.912	3.161	6.485
78	5528.8	2.454	1.909	3.157	6.507
79	5587.3	2.447	1.828	3.153	6.530
80	5645.6	2.439	1.881	3.148	6.552
81	5703.6	2.430	1.869	3.144	6.575
82	5761.3	2.423	1.863	3.140	6.598
83	5818.8	2.415	1.882	3.136	6.620
84	5876.1	2.406	1.930	3.131	6.643
85	5933.1	2.397	1.949	3.127	6.666
86	5989.8	2.389	1.907	3.123	6.689
87	6046.2	2.381	1.863	3.119	6.712
88	6102.4	2.373	1.879	3.115	6.735
89	6158.3	2.364	1.915	3.111	6.758
90	6214.0	2.356	1.855	3.108	6.781
91	6269.4	2.348	1.838	3.104	6.804
92	6324.6	2.340	1.838	3.100	6.827
93	6379.5	2.332	1.918	3.096	6.850
94	6434.1	2.323	1.943	3.092	6.873
95	6488.5	2.314	1.770	3.089	6.897
96	6542.7	2.307	1.835	3.085	6.920
97	6596.6	2.298	1.966	3.082	6.943
98	6650.2	2.289	1.910	3.078	6.967
99	6703.5	2.281	1.865	3.075	6.990

state	v	$E_v\ (cm^{-1})$	$B_v \times 10^2$ (cm^{-1})	$D_v \times 10^8$ (cm^{-1})	R_{min} (Å)	R_{max} (Å)
	100	6756.6	2.273	1.793	3.071	7.014
$(1)^4\Pi$	0	29.6	2.463	1.655	4.579	4.850
	1	89.5	2.456	1.634	4.490	4.958
	2	149.4	2.442	1.675	4.432	5.038
	3	208.7	2.433	1.695	4.386	5.105
	4	267.4	2.420	1.672	4.348	5.166
	5	325.7	2.409	1.747	4.315	5.223
	6	383.4	2.397	1.666	4.285	5.275
	7	440.7	2.384	1.792	4.258	5.326
	8	497.4	2.373	1.709	4.234	5.375
	9	553.6	2.360	1.766	4.211	5.422
	10	609.4	2.348	1.823	4.190	5.468
	11	664.5	2.335	1.755	4.170	5.513
	12	719.2	2.321	1.879	4.152	5.557
	13	773.2	2.309	1.825	4.134	5.601
	14	826.7	2.295	1.842	4.118	5.644
	15	879.7	2.282	1.946	4.102	5.687
	16	932.0	2.268	1.882	4.087	5.729
	17	983.8	2.253	1.931	4.073	5.772
	18	1035.0	2.240	1.998	4.059	5.814
	19	1085.6	2.225	1.952	4.046	5.856
	20	1135.5	2.210	2.019	4.033	5.898
	21	1184.9	2.196	2.077	4.021	5.941
	22	1233.7	2.181	2.069	4.010	5.983
	23	1281.8	2.165	2.089	3.998	6.026
	24	1329.2	2.149	2.204	3.988	6.068
	25	1376.1	2.133	2.172	3.977	6.112
	26	1467.7	2.100	2.306	3.958	6.199
	27	1512.5	2.083	2.359	3.948	6.244
	28	1556.5	2.065	2.275	3.939	6.289
	29	1599.9	2.047	2.440	3.930	6.334
	30	1642.6	2.029	2.518	3.922	6.381
	31	1684.5	2.011	2.499	3.914	6.428
	32	1725.7	1.991	2.540	3.906	6.476
	33	1766.1	1.972	2.708	3.898	6.524
	34	1805.8	1.952	2.790	3.891	6.574
	35	1844.6	1.931	2.750	3.884	6.625
	36	1882.6	1.909	2.839	3.877	6.677
	37	1919.9	1.888	3.021	3.871	6.730
	38	1956.2	1.866	3.044	3.864	6.785
	39	1991.8	1.843	3.158	3.858	6.841

state	v	$E_v\ (cm^{-1})$	$B_v \times 10^2\ (cm^{-1})$	$D_v \times 10^8\ (cm^{-1})$	R_{min} (Å)	R_{max} (Å)
	40	2026.5	1.819	3.231	3.852	6.898
	41	2093.1	1.770	3.517	3.841	7.019
	42	2125.1	1.744	3.646	3.835	7.082
	43	2156.1	1.718	3.715	3.830	7.148
	44	2186.2	1.690	3.890	3.825	7.216
	45	2215.3	1.662	4.072	3.821	7.286
	46	2243.5	1.633	4.199	3.816	7.360
	47	2270.6	1.604	4.386	3.812	7.436
	48	2296.8	1.574	4.574	3.808	7.515
	49	2321.9	1.543	4.709	3.804	7.598
	50	2346.1	1.510	5.069	3.800	7.685
	51	2369.1	1.474	5.570	3.796	7.778
	52	2431.4	1.360	6.252	3.787	8.094
	53	2449.9	1.322	6.659	3.784	8.212
	54	2467.3	1.281	7.396	3.781	8.339
	55	2483.6	1.238	7.509	3.779	8.475
	56	2498.7	1.195	8.395	3.777	8.622
	57	2512.7	1.149	8.616	3.775	8.780
	58	2525.5	1.101	9.608	3.773	8.956
state	v	$E_v\ (cm^{-1})$	$B_v \times 10^2\ (cm^{-1})$	$D_v \times 10^8\ (cm^{-1})$	R_{min} (Å)	R_{max} (Å)
$(2)^4\Pi$	0	20.1	1.866	1.615	5.253	5.583
	1	60.1	1.860	1.632	5.141	5.716
	2	100.0	1.858	1.622	5.066	5.807
	3	139.7	1.856	1.522	5.006	5.883
	4	179.7	1.852	1.587	4.955	5.949
	5	219.7	1.849	1.558	4.911	6.010
	6	259.6	1.846	1.514	4.872	6.065
	7	299.6	1.842	1.521	4.835	6.118
	8	339.5	1.840	1.509	4.802	6.167
	9	379.5	1.836	1.502	4.771	6.214
	10	419.5	1.832	1.425	4.742	6.260
	11	459.5	1.829	1.488	4.717	6.303
	12	499.4	1.825	1.395	4.691	6.345
	13	539.4	1.822	1.529	4.666	6.386
	14	579.4	1.819	1.487	4.642	6.426
	15	619.2	1.816	1.579	4.620	6.465
	16	659.0	1.814	1.489	4.598	6.502
	17	698.7	1.811	1.444	4.578	6.539
	18	738.4	1.807	1.330	4.558	6.576
	19	778.1	1.803	1.473	4.539	6.611
	20	817.7	1.799	1.321	4.521	6.646
	21	857.4	1.795	1.484	4.503	6.681

22	896.9	1.792	1.473	4.486	6.715
23	936.4	1.789	1.414	4.469	6.748
24	975.8	1.785	1.372	4.453	6.782
25	1015.2	1.781	1.404	4.437	6.814
26	1054.5	1.778	1.394	4.422	6.847
27	1093.8	1.774	1.491	4.407	6.878
28	1132.9	1.771	1.464	4.393	6.910
29	1172.0	1.767	1.275	4.379	6.942
30	1211.0	1.763	1.407	4.365	6.973
31	1250.0	1.759	1.341	4.352	7.004
32	1288.9	1.755	1.496	4.339	7.034
33	1327.7	1.751	1.412	4.326	7.065
34	1366.4	1.748	1.359	4.314	7.095
35	1405.0	1.743	1.289	4.301	7.125
36	1443.6	1.739	1.400	4.289	7.155
37	1482.1	1.736	1.498	4.278	7.185
38	1520.5	1.732	1.332	4.266	7.214
39	1558.8	1.728	1.317	4.255	7.244
40	1597.0	1.723	1.383	4.244	7.273
41	1635.1	1.719	1.382	4.233	7.302
42	1673.2	1.715	1.394	4.223	7.331
43	1711.1	1.712	1.383	4.213	7.360
44	1786.8	1.702	1.375	4.192	7.417
45	1824.4	1.697	1.429	4.183	7.446
46	1899.4	1.690	1.291	4.164	7.504
47	1936.7	1.684	1.413	4.154	7.533
48	2010.9	1.676	1.387	4.136	7.590
49	2047.9	1.671	1.440	4.128	7.618
50	2121.5	1.662	1.327	4.110	7.675
51	2158.1	1.657	1.331	4.102	7.703
52	2231.0	1.648	1.372	4.086	7.761
53	2267.3	1.644	1.273	4.078	7.789
54	2303.4	1.638	1.334	4.070	7.818
55	2339.5	1.634	1.420	4.062	7.846
56	2375.4	1.630	1.388	4.054	7.874
57	2411.2	1.625	1.343	4.047	7.902
58	2446.9	1.621	1.418	4.039	7.931
59	2482.4	1.615	1.330	4.032	7.959
60	2517.9	1.610	1.297	4.025	7.987
61	2553.2	1.606	1.391	4.018	8.016
62	2588.4	1.602	1.513	4.011	8.044
63	2623.4	1.596	1.308	4.004	8.073
64	2693.1	1.587	1.375	3.990	8.129

	65	2727.8	1.582	1.442	3.984	8.158
	66	2762.3	1.577	1.450	3.977	8.186
	67	2796.7	1.572	1.344	3.970	8.215
	68	2831.0	1.567	1.279	3.964	8.244
	69	2865.2	1.562	1.491	3.958	8.273
	70	2899.1	1.556	1.433	3.951	8.301
	71	2933.0	1.552	1.290	3.945	8.330
	72	2966.7	1.546	1.580	3.939	8.360
	73	3000.2	1.541	1.281	3.933	8.389
	74	3033.6	1.535	1.326	3.927	8.418
	75	3066.9	1.530	1.532	3.921	8.447
	76	3100.0	1.524	1.444	3.916	8.477
	77	3165.7	1.514	1.323	3.905	8.536
	78	3198.4	1.508	1.421	3.899	8.566
	79	3230.9	1.502	1.569	3.893	8.597
	80	3263.1	1.496	1.495	3.888	8.627
	81	3295.2	1.491	1.435	3.883	8.657
	82	3327.2	1.485	1.496	3.877	8.688
	83	3359.0	1.479	1.416	3.872	8.718
	84	3390.6	1.473	1.342	3.867	8.749
	85	3422.1	1.468	1.481	3.862	8.780
	86	3453.4	1.461	1.677	3.857	8.812
	87	3484.5	1.454	1.612	3.852	8.844
	88	3515.4	1.448	1.593	3.847	8.876
	89	3546.1	1.441	1.501	3.842	8.908
	90	3576.6	1.435	1.479	3.837	8.940
	91	3607.0	1.428	1.647	3.833	8.973
	92	3637.1	1.422	1.542	3.828	9.006
	93	3667.0	1.415	1.593	3.823	9.039
	94	3696.8	1.408	1.639	3.819	9.073
	95	3726.3	1.401	1.596	3.814	9.107
	96	3755.6	1.394	1.625	3.810	9.141
	97	3784.7	1.386	1.672	3.805	9.176
	98	3813.5	1.379	1.602	3.801	9.211
	99	3842.2	1.372	1.756	3.797	9.247
	100	3870.6	1.364	1.714	3.792	9.283
state	v	$E_v\ (cm^{-1})$	$B_v \times 10^2$ (cm^{-1})	$D_v \times 10^8$ (cm^{-1})	R_{min} (Å)	R_{max} (Å)
$(3)^4\Pi$	0	24.0	2.059	1.550	5.006	5.312
	1	71.3	2.052	1.566	4.906	5.431
	2	118.3	2.045	1.553	4.836	5.520
	3	165.1	2.035	1.622	4.784	5.595
	4	211.4	2.028	1.389	4.741	5.661

5	258.1	2.020	1.516	4.705	5.718
6	304.5	2.013	1.598	4.670	5.780
7	350.5	2.000	1.833	4.638	5.833
8	440.8	1.987	1.572	4.583	5.935
9	485.8	1.978	1.782	4.558	5.985
10	530.1	1.967	1.515	4.536	6.033
11	574.2	1.958	1.465	4.514	6.080
12	618.2	1.949	1.825	4.494	6.126
13	661.6	1.940	1.495	4.474	6.171
14	704.8	1.932	1.869	4.456	6.216
15	747.6	1.922	1.468	4.438	6.259
16	790.1	1.912	1.712	4.421	6.303
17	832.3	1.905	1.821	4.404	6.346
18	874.1	1.897	1.648	4.389	6.387
19	915.6	1.886	1.612	4.374	6.436
20	956.8	1.875	1.726	4.359	6.472
21	997.7	1.867	1.758	4.345	6.514
22	1038.2	1.857	1.800	4.332	6.555
23	1078.3	1.848	1.504	4.318	6.597
24	1118.2	1.837	2.093	4.306	6.639
25	1157.6	1.827	1.687	4.294	6.680
26	1196.6	1.816	1.789	4.582	6.722
27	1235.4	1.808	1.839	4.270	6.764
28	1273.7	1.794	1.871	4.259	6.805
29	1311.6	1.787	1.831	4.248	6.848
30	1349.2	1.774	1.628	4.238	6.890
31	1386.5	1.763	2.169	4.227	6.932
32	1423.3	1.754	1.746	4.217	6.975
33	1459.7	1.741	1.975	4.207	7.017
34	1495.7	1.730	1.955	4.198	7.059
35	1531.4	1.721	1.896	4.189	7.104
36	1566.7	1.706	1.761	4.180	7.146
37	1636.1	1.685	2.136	4.163	7.235
38	1670.1	1.673	1.824	4.154	7.279
39	1703.8	1.660	2.314	4.146	7.324
40	1736.9	1.646	1.868	4.139	7.370
41	1769.7	1.634	2.514	4.131	7.417
42	1802.0	1.620	2.383	4.123	7.465
43	1833.6	1.602	2.159	4.116	7.514
44	1864.8	1.590	2.225	4.109	7.564
45	1895.6	1.574	3.017	4.102	7.616
46	1925.7	1.559	1.908	4.096	7.668
47	1955.4	1.546	2.218	4.089	7.721

	48	1984.7	1.533	2.329	4.083	7.772
	49	2013.5	1.520	2.016	4.076	7.821
	50	2042.1	1.507	2.812	4.070	7.874
	51	2070.0	1.491	2.260	4.065	7.932
	52	2097.5	1.475	2.911	4.059	7.985
	53	2151.1	1.451	2.265	4.048	8.093
	54	2177.4	1.437	1.966	4.043	8.147
	55	2203.3	1.422	2.899	4.037	8.205
	56	2228.7	1.406	2.563	4.032	8.262
	57	2278.4	1.381	2.252	4.023	8.379
	58	2302.6	1.366	2.177	4.018	8.435
	59	2326.5	1.352	2.610	4.013	8.496
	60	2349.9	1.338	3.112	4.009	8.557
	61	2372.9	1.323	2.565	4.004	8.618
	62	2395.5	1.311	2.295	4.000	8.679
	63	2417.8	1.298	1.798	3.996	8.740
	64	2439.8	1.284	2.758	3.992	8.801
	65	2461.4	1.269	3.222	3.988	8.866
	66	2482.5	1.254	2.301	3.984	8.936
	67	2503.2	1.236	3.058	3.980	8.999
	68	2543.5	1.214	2.248	3.973	9.134
	69	2563.2	1.197	2.223	3.969	9.200
	70	2582.5	1.184	2.603	3.966	9.271
	71	2601.5	1.174	2.606	3.962	9.338
	72	2620.1	1.159	2.422	3.959	9.408
	73	2638.5	1.147	2.703	3.956	9.480

state	v	$E_v\ (cm^{-1})$	$B_v \times 10^2$ (cm^{-1})	$D_v \times 10^8$ (cm^{-1})	R_{min} (Å)	R_{max} (Å)
$(1)^4\Delta$	0	41.3	3.289	2.086	3.966	4.196
	1	123.7	3.279	2.065	3.888	4.287
	2	206.1	3.269	2.091	3.837	4.354
	3	287.9	3.258	2.094	3.797	4.409
	4	369.4	3.248	2.050	3.763	4.458
	5	450.6	3.238	2.093	3.733	4.503
	6	531.5	3.228	2.075	3.707	4.545
	7	612.0	3.218	2.071	3.682	4.584
	8	692.3	3.208	2.078	3.660	4.622
	9	772.1	3.197	2.039	3.640	4.658
	10	851.8	3.187	2.096	3.620	4.693
	11	931.0	3.177	2.064	3.602	4.726
	12	1010.0	3.168	2.026	3.585	4.758
	13	1088.7	3.158	2.101	3.568	4.790
	14	1167.0	3.148	2.120	3.553	4.822

15	1244.9	3.135	2.154	3.538	4.852
16	1322.3	3.125	2.052	3.524	4.883
17	1399.5	3.116	2.063	3.510	4.912
18	1476.3	3.105	2.121	3.497	4.941
19	1552.7	3.094	2.116	3.485	4.970
20	1628.8	3.085	2.067	3.472	4.998
21	1704.6	3.074	2.116	3.461	5.027
22	1779.9	3.063	2.124	3.449	5.054
23	1854.9	3.053	2.027	3.438	5.082
24	1929.6	3.043	2.145	3.428	5.109
25	2003.9	3.031	2.060	3.417	5.136
26	2077.9	3.022	2.109	3.407	5.163
27	2151.6	3.011	2.081	3.398	5.189
28	2224.9	3.001	2.062	3.388	5.215
29	2297.9	2.991	2.136	3.379	5.241
30	2370.5	2.980	2.032	3.370	5.267
31	2442.8	2.971	2.098	3.361	5.293
32	2514.8	2.959	2.074	3.352	5.319
33	2586.4	2.950	2.115	3.344	5.344
34	2657.8	2.939	2.036	3.336	5.369
35	2728.8	2.929	2.052	3.328	5.395
36	2799.4	2.919	2.135	3.320	5.420
37	2869.8	2.908	2.034	3.312	5.445
38	2939.8	2.898	2.109	3.305	5.470
39	3009.5	2.888	2.025	3.297	5.494
40	3078.8	2.877	2.094	3.290	5.519
41	3147.9	2.867	2.039	3.283	5.544
42	3216.6	2.857	2.072	3.276	5.568
43	3285.0	2.847	2.073	3.269	5.593
44	3353.1	2.836	2.102	3.263	5.617
45	3420.9	2.826	1.994	3.256	5.642
46	3488.3	2.815	2.139	3.250	5.666
47	3555.4	2.805	2.070	3.244	5.691
48	3622.1	2.794	1.989	3.237	5.715
49	3688.6	2.784	2.051	3.231	5.739
50	3754.8	2.775	1.946	3.225	5.763
51	3820.6	2.765	2.074	3.220	5.787
52	3886.2	2.755	1.968	3.214	5.811
53	3951.5	2.745	2.134	3.208	5.836
54	4016.5	2.734	1.982	3.203	5.859
55	4081.1	2.724	2.009	3.197	5.883
56	4145.5	2.715	1.994	3.192	5.907
57	4209.6	2.704	2.043	3.186	5.931

58	4273.4	2.694	2.038	3.181	5.955
59	4336.8	2.685	1.909	3.176	5.979
60	4400.0	2.675	2.082	3.171	6.003
61	4462.9	2.664	1.964	3.166	6.027
62	4525.5	2.655	1.976	3.161	6.051
63	4587.8	2.645	2.032	3.156	6.075
64	4649.8	2.634	1.987	3.151	6.099
65	4711.5	2.625	1.921	3.147	6.122
66	4772.9	2.615	2.037	3.142	6.146
67	4834.0	2.604	1.966	3.137	6.170
68	4894.9	2.595	1.964	3.133	6.194
69	4955.4	2.585	1.939	3.129	6.218
70	5015.7	2.575	1.989	3.124	6.242
71	5075.6	2.566	1.961	3.120	6.266
72	5135.3	2.555	1.966	3.116	6.289
73	5194.7	2.546	1.914	3.111	6.313
74	5253.8	2.536	1.999	3.107	6.337
75	5312.7	2.526	1.960	3.103	6.361
76	5371.2	2.517	1.955	3.099	6.385
77	5429.5	2.506	1.888	3.095	6.409
78	5487.5	2.497	1.996	3.091	6.433
79	5545.1	2.487	1.942	3.087	6.457
80	5602.5	2.477	1.854	3.083	6.481
81	5659.7	2.467	1.964	3.080	6.506
82	5716.5	2.458	1.885	3.076	6.530
83	5773.1	2.448	1.979	3.072	6.554
84	5829.4	2.438	1.882	3.069	6.578
85	5885.5	2.429	1.972	3.065	6.602
86	5941.2	2.419	1.832	3.062	6.627
87	5996.7	2.409	2.009	3.058	6.651
88	6051.8	2.399	1.854	3.055	6.675
89	6106.7	2.390	1.922	3.051	6.700
90	6161.4	2.380	1.869	3.048	6.724
91	6215.7	2.370	1.972	3.044	6.749
92	6269.8	2.360	1.800	3.041	6.773
93	6323.6	2.351	1.971	3.038	6.798
94	6377.1	2.341	1.865	3.035	6.822
95	6430.4	2.332	1.885	3.031	6.847
96	6483.3	2.322	1.938	3.028	6.872
97	6536.0	2.312	1.868	3.025	6.897
98	6588.4	2.302	1.854	3.022	6.922
99	6640.6	2.292	1.899	3.019	6.947
100	6692.5	2.283	1.859	3.016	6.972

state	v	$E_v\ (cm^{-1})$	$B_v \times 10^2$ (cm^{-1})	$D_v \times 10^8$ (cm^{-1})	R_{min} (Å)	R_{max} (Å)
$(1)^4\Sigma^-$	0	17.5	2.275	3.972	4.698	5.085
	1	86.9	2.233	2.675	4.554	5.336
	2	122.9	2.203	3.556	4.503	5.433
	3	158.2	2.193	4.226	4.460	5.522
	4	192.5	2.175	3.078	4.425	5.602
	5	260.7	2.129	4.209	4.363	5.751
	6	294.0	2.114	3.993	4.336	5.824
	7	326.5	2.087	3.240	4.313	5.895
	8	358.6	2.060	3.264	4.290	5.967
	9	390.3	2.042	4.683	4.270	6.037
	10	421.1	2.023	3.974	4.251	6.107
	11	451.4	1.999	3.132	4.233	6.176
	12	481.2	1.971	3.906	4.216	6.246
	13	510.5	1.950	4.328	4.200	6.315
	14	539.2	1.931	4.954	4.185	6.385
	15	567.1	1.909	3.456	4.171	6.455
	16	594.6	1.880	3.633	4.158	6.526
	17	647.9	1.836	5.170	4.133	6.669
	18	673.6	1.815	4.446	4.121	6.742
	19	698.7	1.787	3.520	4.110	6.815
	20	747.3	1.736	5.088	4.089	6.967
	21	770.6	1.713	5.510	4.079	7.045
	22	793.4	1.692	5.354	4.070	7.121
	23	815.6	1.669	3.934	4.061	7.201
	24	837.4	1.641	4.451	4.053	7.281
	25	858.6	1.615	5.091	4.045	7.361
	26	879.3	1.595	5.058	4.037	7.442
	27	899.5	1.572	5.635	4.029	7.525
	28	919.1	1.547	5.437	4.022	7.610
	29	938.3	1.523	4.745	4.015	7.696
	30	956.9	1.495	4.837	4.009	7.784
	31	975.0	1.470	4.622	4.002	7.874
	32	992.6	1.445	5.853	3.996	7.965
	33	1009.8	1.422	5.080	3.990	8.058
	34	1026.5	1.398	6.048	3.984	8.151
	35	1042.7	1.377	6.175	3.979	8.246

Table S3: Values of the eigenvalues, the rotational constants and, and the abscissas of the turning points for the different vibrational levels of states $X^2\Sigma^+$, $(2)^2\Sigma^+$, $(3)^2\Sigma^+$, $(1)^2\Pi$, $(2)^2\Pi$, $(2)^2\Sigma^-$, $(2)^4\Sigma^+$, $(1)^4\Pi$, $(2)^4\Pi$, $(3)^4\Pi$, and $(1)^4\Sigma^-$ of CaNa molecule.

States Λ^{2S+1}	v	E_v (cm^{-1})	$B_v \times 10^2$ (cm^{-1})	$\Delta B_v / B_v$ %	$D_v \times 10^7$ (cm^{-1})	R_{min} (Å)	R_{max} (Å)
$(X)^2\Sigma^+$	0	50.16	8.1153[a]<br>8.3[c]	2.2	2.1733	3.6177	3.9215
	1	148.39	8.0002		2.2465	3.5273	4.0603
	2	243.80	7.8833		2.3172	3.4705	4.1662
	3	336.41	7.7618		2.4431	3.4274	4.2620
	4	425.88	7.6298		2.5727	3.3923	4.3524
	5	512.07	7.4978[a]<br>7.5[c]	0.02	2.6315	3.3627	4.4400
	6	595.22	7.3610		2.8042	3.3371	4.5264
	7	675.13	7.2201		2.8744	3.3146	4.6125
	8	751.94	7.0754		3.0506	3.2945	4.6994
	9	825.57	6.9296		3.1168	3.2765	4.7879
	10	896.17	6.7797		3.2903	3.2602	4.8746
	11	963.66	6.6233		3.5724	3.2454	4.9679
	12	1027.85	6.4592		3.5987	3.2320	5.0618
	13	1088.98	6.3004		3.8551	3.2197	5.1605
	14	1146.93	6.1207		4.3151	3.2085	5.2630
	15	1201.49	5.9508		4.0614	3.1982	5.3707
	16	1253.12	5.7696		4.6689	3.1888	5.4817
	17	1301.64	5.6017		4.3484	3.1802	5.5976
	18	1347.49	5.4212		5.0328	3.1723	5.7158
	19	1390.41	5.2397		5.3667	3.1650	5.8454
	20	1430.33	5.0377		5.6086	3.1584	5.9817
	21	1467.36	4.8509		5.5118	3.1524	6.1255
$(2)\,^2\Sigma^+$	0	65.26	7.5430		1.1063	3.7778	4.0481
	1	189.64	7.5212		1.1703	3.6841	4.1572
	2	311.79	7.4996		1.1875	3.6224	4.2376
	3	432.51	7.4667		1.1970	3.5739	4.3051
	4	552.10	7.4449		1.1820	3.5330	4.3650
	5	671.00	7.4169		1.2038	3.4973	4.4200
	6	789.03	7.3895		1.1978	3.4653	4.4713
	7	906.34	7.3649		1.2102	3.4363	4.5198
	8	1022.88	7.3338		1.1998	3.4096	4.5663
	9	1138.76	7.3097		1.2381	3.3879	4.6110
	10	1253.79	7.2779		1.1865	3.3618	4.6543
	11	1368.24	7.2511		1.2609	3.3401	4.6964
	12	1481.81	7.2219		1.1878	3.3197	4.7376
	13	1594.82	7.1924		1.2343	3.3004	4.7780
	14	1707.12	7.1655		1.2245	3.2820	4.8150
	15	1818.74	7.1345		1.2398	3.2645	4.8549
	16	1929.58	7.0995		1.3116	3.2477	4.8937
	17	2039.41	7.0648		1.2939	3.2318	4.9322
	18	2148.33	7.0318		1.1718	3.2165	4.9705
	19	2256.76	7.0052		1.2737	3.2017	5.0069
	20	2364.45	6.9725		1.3128	3.1876	5.0443
	21	2471.16	6.9289		1.3019	3.1739	5.0815

	22	2576.97	6.8982		1.3302	3.1608	5.1186
	23	2681.88	6.8599		1.3157	3.1481	5.1556
	24	2785.83	6.8175		1.2977	3.1359	5.1925
	25	2888.98	6.7904		1.3317	3.1240	5.2295
	26	2991.24	6.7442		1.4092	3.1126	5.2665
	27	3092.36	6.7021		1.2780	3.1015	5.3035
	28	3192.72	6.6710		1.4539	3.0907	5.3411
	29	3291.97	6.6195		1.3647	3.0803	5.3784
	30	3390.21	6.5838		1.3121	3.0702	5.4158
	31	3487.63	6.5410		1.5229	3.0604	5.4535
	32	3583.88	6.4995		1.3020	3.0508	5.4915
	33	3679.26	6.4525		1.5150	3.0416	5.5298
	34	3773.45	6.4075		1.4203	3.0326	5.5684
	35	3866.64	6.3639		1.5100	3.0238	5.6076
	36	3958.71	6.3175		1.3612	3.0153	5.6457
	37	4049.80	6.2661		1.6163	3.0070	5.6859
	38	4139.64	6.2223		1.5042	2.9990	5.7265
	39	4228.35	6.1639		1.6789	2.9911	5.7676
	40	4315.64	6.1106		1.4719	2.9835	5.8093
	41	4401.78	6.0528		1.7151	2.9761	5.8517
	42	4486.61	6.0084		1.6561	2.9689	5.8948
	43	4570.14	5.9396		1.5834	2.9620	5.9388
	44	4652.35	5.8848		1.8238	2.9552	5.9836
	45	4733.11	5.8224		1.6236	2.9486	6.0294
	46	4812.59	5.7625		2.0762	2.9422	6.0763
	47	4890.39	5.6968		1.4865	2.9360	6.1242
	48	4966.93	5.6245		2.0102	2.9299	6.1735
	49	5041.83	5.5614		2.0679	2.9241	6.2241
	50	5115.10	5.4887		1.8291	2.9184	6.2762
	51	5186.83	5.4122		2.1590	2.9129	6.3299
	52	5256.80	5.3385		1.9588	2.9076	6.3854
	53	5325.12	5.2527		2.1726	2.9024	6.4428
	54	5391.66	5.1746		2.4437	2.8975	6.5024
	55	5456.31	5.0911		2.3011	2.8927	6.5643
	56	5519.08	4.9976		2.1851	2.8881	6.6288
	57	5579.96	4.9023		2.6110	2.8837	6.6962
	58	5638.80	4.8095		2.6794	2.8794	6.7668
	59	5695.54	4.7050		2.6937	2.8753	6.8410
	60	5750.14	4.5983		3.0170	2.8714	6.9196
	61	5802.43	4.4856		3.0559	2.8677	7.0031
	62	5852.36	4.3632		3.2319	2.8642	7.0924
	63	5899.82	4.2360		3.4696	2.8609	7.1882
(3) $^2\Sigma^+$	0	52.91	7.0153		1.4038	3.9069	4.2049
	1	151.50	6.9483		1.6402	3.8058	4.3430
	2	245.15	6.9067		1.5019	3.7412	4.4403
	3	338.14	6.8492		1.6574	3.6898	4.5240
	4	429.16	6.8098		1.6061	3.6470	4.5991
	5	519.01	6.7528		1.6383	3.6096	4.6691
	6	607.63	6.7113		1.7189	3.5763	4.7353
	7	694.84	6.6600		1.6203	3.5462	4.7970
	8	781.12	6.6118		1.7931	3.5185	4.8585
	9	865.97	6.5633		1.7539	3.4929	4.9203
	10	949.55	6.5057		1.7755	3.4692	4.9775

	11	1031.93	6.4607		1.8117	3.4469	5.0352
	12	1113.16	6.4099		1.8143	3.4259	5.0924
	13	1193.16	6.3499		1.9339	3.4062	5.1490
	14	1271.80	6.3015		1.8586	3.3875	5.2052
	15	1349.33	6.2480		1.8719	3.3697	5.2611
	16	1425.74	6.1939		1.8942	3.3528	5.3157
	17	1501.08	6.1470		1.9437	3.3366	5.3703
	18	1575.31	6.0919		1.8742	3.3211	5.4251
	19	1648.50	6.0355		2.1220	3.3063	5.4804
	20	1720.32	5.9741		2.0490	3.2921	5.5365
	21	1790.93	5.9193		2.0186	3.2786	5.5929
	22	1860.38	5.8569		2.0210	3.2655	5.6458
	23	1928.72	5.8017		2.2456	3.2530	5.7051
	24	1995.71	5.7329		2.1922	3.2410	5.7645
	25	2061.30	5.6577		2.4781	3.2295	5.8242
	26	2125.28	5.5885		2.0030	3.2186	5.8842
	27	2188.25	5.5340		2.2942	3.2079	5.9451
	28	2249.99	5.4638		2.3099	3.1978	6.0065
	29	2310.39	5.3914		2.1323	3.1880	6.0684
	30	2369.70	5.3346		2.3466	3.1786	6.1311
	31	2427.81	5.2629		2.3254	3.1695	6.1943
	32	2484.70	5.1985		2.1111	3.1607	6.2582
	33	2540.58	5.1331		2.4659	3.1523	6.3228
	34	2595.20	5.0642		2.0507	3.1442	6.3880
	35	2648.87	5.0010		2.5869	3.1363	6.4542
	36	2701.30	4.9342		2.0917	3.1287	6.5209
	37	2752.76	4.8672		2.4169	3.1214	6.5887
	38	2803.07	4.7989		2.1583	3.1144	6.6573
	39	2852.39	4.7316		2.6139	3.1076	6.7271
	40	2900.55	4.6666		2.2657	3.1010	6.7980
	41	2947.67	4.5920		2.4180	3.0947	6.8704
	42	2993.67	4.5234		2.4968	3.0885	6.9446
	43	3038.54	4.4476		2.5247	3.0827	7.0206
	44	3082.27	4.3747		2.6582	3.0770	7.0988
	45	3124.79	4.2958		2.4980	3.0715	7.1791
	46	3166.16	4.2158		2.9469	3.0663	7.2621
	47	3206.25	4.1375		2.9901	3.0613	7.3477
(1) $^2\Pi$	0	88.43	10.494		1.5734	3.2023	3.4314
	1	259.36	10.443		1.7205	3.1234	3.5289
	2	425.43	10.401		1.6617	3.0721	3.5985
	3	590.28	10.350		1.7194	3.0316	3.6575
	4	753.33	10.309		1.7040	2.9975	3.7101
	5	915.09	10.260		1.7083	2.9677	3.7584
	6	1075.64	10.217		1.7380	2.9411	3.8036
	7	1234.87	10.170		1.7062	2.9170	3.8465
	8	1393.05	10.124		1.7510	2.8949	3.8875
	9	1549.99	10.079		1.7255	2.8744	3.9270
	10	1705.86	10.032		1.7415	2.8552	3.9652
	11	1860.63	9.9873		1.7553	2.8372	4.0024
	12	2014.27	9.9409		1.7274	2.8203	4.0387
	13	2166.92	9.8958		1.7507	2.8042	4.0742
	14	2318.55	9.8529		1.7249	2.7889	4.1084
	15	2469.27	9.8083		1.7297	2.7744	4.1423

	16	2619.03	9.7632		1.7872	2.7604	4.1761
	17	2767.66	9.7133		1.8034	2.7471	4.2095
	18	2915.05	9.6611		1.7964	2.7343	4.2425
	19	3061.29	9.6140		1.7497	2.7220	4.2752
	20	3206.54	9.5671		1.7534	2.7105	4.3077
	21	3350.77	9.5154		1.8154	2.6994	4.3399
	22	3493.82	9.4648		1.7675	2.6885	4.3719
	23	3635.82	9.4167		1.7659	2.6778	4.4037
	24	3776.80	9.3655		1.8515	2.6675	4.4354
	25	3916.58	9.3157		1.8140	2.6575	4.4670
	26	4055.29	9.2676		1.8518	2.6478	4.4985
	27	4192.87	9.2166		1.9046	2.6384	4.5299
	28	4329.25	9.1686		1.8571	2.6293	4.5613
	29	4464.53	9.1174		1.8853	2.6205	4.5926
	30	4598.63	9.0647		1.8315	2.6118	4.6240
	31	4731.63	9.0125		1.8265	2.6034	4.6554
	32	4863.49	8.9555		1.7574	2.5954	4.6869
	33	4994.28	8.9007		1.7606	2.5878	4.7185
	34	5124.00	8.8439		1.7754	2.5805	4.7492
	35	5252.62	8.7883		1.7860	2.5735	4.7811
	36	5380.10	8.7272		1.9000	2.5667	4.8130
	37	5506.27	8.6654		1.9712	2.5600	4.8460
	38	5631.04	8.6013		1.9467	2.5531	4.8786
	39	5754.48	8.5437		1.8427	2.5462	4.9108
	40	5876.79	8.4874		1.9270	2.5394	4.9432
	41	5997.89	8.4279		2.0697	2.5329	4.9766
	42	6117.57	8.3633		2.0726	2.5266	5.0099
	43	6235.87	8.3047		2.1197	2.5204	5.0438
	44	6352.82	8.2433		2.3131	2.5143	5.0781
	45	6468.21	8.1784		2.4082	2.5084	5.1128
	46	6581.99	8.1145		2.3170	2.5027	5.1480
	47	6694.26	8.0494		2.3572	2.4971	5.1837
	48	6804.98	7.9823		2.3304	2.4916	5.2201
	49	6914.10	7.9066		2.3719	2.4863	5.2571
	50	7021.49	7.8289		2.1730	2.4811	5.2947
	51	7127.31	7.7503		2.2667	2.4761	5.3334
	52	7231.39	7.6608		2.3558	2.4712	5.3728
	53	7333.64	7.5784		2.3414	2.4664	5.4133
	54	7434.11	7.4863		2.5499	2.4617	5.4549
	55	7532.61	7.3951		2.6007	2.4572	5.4977
	56	7629.10	7.2971		2.8175	2.4528	5.5420
	57	7723.43	7.1984		2.9309	2.4485	5.5879
	58	7815.53	7.0931		3.0536	2.4444	5.6355
	59	7905.32	6.9856		3.2456	2.4404	5.6850
	60	7992.62	6.8663		3.1983	2.4366	5.7370
	61	8077.41	6.7444		3.6817	2.4329	5.7916
	62	8159.39	6.6132		3.4997	2.4293	5.8490
	63	8238.61	6.4728		3.9710	2.4259	5.9098
	64	8646.02	5.4477		5.6324	2.4087	6.3756
	65	8701.35	5.2477		5.6493	2.4064	6.4770
(2) $^2\Pi$	0	66.88	9.0659		1.8161	3.4351	3.7000
	1	194.23	8.9797		1.9868	3.3488	3.8187
	2	317.11	8.9020		1.9660	3.2938	3.9063

	3	437.74	8.8136		2.0302	3.2511	3.9823
	4	555.96	8.7329		2.0778	3.2156	4.0519
	5	671.86	8.6461		2.0623	3.1851	4.1164
	6	785.75	8.5591		2.1733	3.1581	4.1791
	7	897.28	8.4683		2.2368	3.1340	4.2403
	8	1006.33	8.3693		2.2512	3.1122	4.3003
	9	1113.03	8.2751		2.3179	3.0922	4.3596
	10	1217.39	8.1771		2.3933	3.0738	4.4186
	11	1319.28	8.0736		2.3917	3.0568	4.4776
	12	1418.84	7.9713		2.5240	3.0410	4.5368
	13	1515.91	7.8653		2.5412	3.0262	4.5963
	14	1610.58	7.7589		2.5719	3.0124	4.6564
	15	1702.88	7.6506		2.5911	2.9995	4.7175
	16	1792.94	7.5462		2.7016	2.9873	4.7759
	17	1880.64	7.4323		2.8593	2.9758	4.8418
	18	1965.72	7.3044		3.0277	2.9649	4.9022
	19	2048.07	7.1888		2.6523	2.9547	4.9686
	20	2128.50	7.0930		2.7514	2.9450	5.0314
	21	2206.93	6.9729		3.2082	2.9358	5.0976
	22	2282.79	6.8542		2.7276	2.9271	5.1640
	23	2356.77	6.7494		3.0170	2.9188	5.2311
	24	2428.71	6.6403		2.7491	2.9108	5.2982
	25	2498.98	6.5445		2.9574	2.9032	5.3655
	26	2567.44	6.4382		2.6095	2.8960	5.4325
	27	2634.49	6.3534		2.8499	2.8890	5.4995
	28	2699.99	6.2533		2.5144	2.8823	5.5662
	29	2764.24	6.1731		2.7920	2.8758	5.6332
	30	2827.14	6.0846		2.4444	2.8695	5.6977
	31	2888.93	6.0043		2.8163	2.8635	5.7647
	32	2949.36	5.9125		2.6355	2.8577	5.8318
	33	3008.49	5.8221		2.7624	2.8520	5.8993
	34	3066.33	5.7396		2.6022	2.8466	5.9675
	35	3232.27	5.4700		2.9572	2.8314	6.1793
	36	3284.88	5.3745		3.1493	2.8267	6.2534
	37	3335.98	5.2743		2.8989	2.8221	6.3297
	38	3385.67	5.1706		3.5451	2.8178	6.4089
(2) $^2\Sigma^-$	0	56.72	8.5200		2.0329	3.5380	3.8254
	1	166.75	8.4809		2.1041	3.4409	3.9452
	2	275.25	8.4536		1.9595	3.3777	4.0293
(2) $^4\Sigma^+$	0	45.99	7.3145		1.8668	3.8136	4.1306
	1	137.07	7.2504		1.8855	3.7139	4.2663
	2	226.83	7.1858		1.9045	3.6497	4.3672
	3	315.27	7.1207		1.9223	3.6000	4.4542
	4	402.39	7.0551		1.9411	3.5587	4.5334
	5	488.20	6.9889		1.9614	3.5231	4.6075
	6	572.68	6.9221		1.9817	3.4916	4.6781
	7	655.85	6.8546		2.0034	3.4634	4.7463
	8	737.67	6.7862		2.0270	3.4378	4.8127
	9	818.15	6.7166		2.0534	3.4143	4.8778
	10	897.27	6.6464		2.0752	3.3926	4.9421
	11	975.02	6.5756		2.0902	3.3725	5.0055
	12	1051.43	6.5046		2.1052	3.3537	5.0685
	13	1126.52	6.4331		2.1224	3.3361	5.1311

	14	1200.27	6.3610		2.1374	3.3195	5.1934
	15	1272.71	6.2884		2.1523	3.3038	5.2557
	16	1343.84	6.2152		2.1673	3.2895	5.3181
	17	1413.66	6.1414		2.1880	3.2759	5.3806
	18	1482.18	6.0673		2.2033	3.2625	5.4434
	19	1549.40	5.9930		2.2196	3.2497	5.5065
	20	1615.35	5.9193		2.2171	3.2376	5.5700
	21	1680.06	5.8486		2.1536	3.2261	5.6339
	22	1743.71	5.7860		1.9893	3.2148	5.6982
	23	1806.56	5.7339		1.8676	3.2041	5.7437
	24	1868.78	5.6792		2.1784	3.1942	5.8096
	25	1929.88	5.5918		2.9805	3.1848	5.8851
	26	1988.90	5.4800		2.6833	3.1758	5.9512
	27	2046.41	5.4377		8.2615	3.1667	6.0002
	28	2104.45	5.4400		1.5028	3.1577	6.0642
	29	2162.19	5.3466		3.6182	3.1490	6.1314
	30	2217.41	5.2187		2.6371	3.1409	6.2002
	31	2271.01	5.1514		2.4420	3.1331	6.2711
	32	2323.35	5.0570		2.6028	3.1256	6.3443
	33	2374.34	4.9886		2.7039	3.1184	6.4194
	34	2423.84	4.8715		3.0818	3.1115	6.4962
	35	2471.80	4.8218		2.1276	3.1050	6.5746
	36	2518.72	4.6994		3.7999	3.0986	6.6555
	37	2563.80	4.6469		1.9574	3.0926	6.7378
(1) $^4\Pi$	0	58.80	8.2863		1.7984	3.5901	3.8729
	1	170.56	8.2013		1.9619	3.4989	4.0003
	2	278.36	8.1233		1.9479	3.4408	4.0949
	3	384.00	8.0345		1.9977	3.3959	4.1773
	4	487.42	7.9497		2.0355	3.3588	4.2531
	5	588.65	7.8558		1.9846	3.3272	4.3248
	6	687.98	7.7565		2.0125	3.2991	4.3940
	7	785.30	7.6550		2.0043	3.2827	4.4617
	8	880.61	7.5477		2.0264	3.2605	4.5285
	9	973.87	7.4417		2.1861	3.2391	4.5950
	10	1064.82	7.3415		2.3784	3.2195	4.6613
	11	1153.31	7.2450		2.5581	3.2014	4.7276
	12	1239.27	7.1519		2.7381	3.1868	4.7940
	13	1322.66	7.0598		2.8139	3.1724	4.8605
	14	1403.54	6.9588		2.7359	3.1576	4.9281
	15	1481.95	6.8429		2.6359	3.1441	4.9972
	16	1557.94	6.7166		2.6296	3.1315	5.0682
	17	1631.46	6.5870		2.7595	3.1196	5.1414
	18	1702.46	6.4558		3.0195	3.1085	5.2170
	19	1770.81	6.3252		3.3611	3.0982	5.2952
	20	1836.40	6.1954		3.7511	3.0885	5.3765
	21	1899.14	6.0635		4.0549	3.0794	5.4610
	22	1958.94	5.9233		4.3032	3.0710	5.5494
	23	2015.63	5.7599		4.7810	3.0631	5.6418
	24	2068.65	5.5550		5.3932	3.0559	5.7669
	25	2117.61	5.3563		3.9860	3.0494	5.8605
	26	2164.03	5.2230		4.2423	3.0433	5.9957
(2) $^4\Pi$	0	57.70	7.1110		1.1724	3.8869	4.1727
	1	168.24	7.0853		1.2552	3.7880	4.2913

	2	276.40	7.0639		1.2199	3.7232	4.3758
	3	384.00	7.0380		1.2491	3.6720	4.4468
	4	490.77	7.0165		1.2403	3.6288	4.5098
	5	596.93	6.9906		1.2400	3.5910	4.5675
	6	702.51	6.9679		1.2579	3.5572	4.6214
	7	807.46	6.9428		1.2362	3.5265	4.6724
	8	911.90	6.9180		1.2637	3.4983	4.7210
	9	1015.72	6.8941		1.2469	3.4721	4.7677
	10	1119.02	6.8679		1.2533	3.4477	4.8129
	11	1221.76	6.8436		1.2694	3.4247	4.8567
	12	1323.91	6.8170		1.2525	3.4031	4.8995
	13	1425.50	6.7894		1.2863	3.3826	4.9415
	14	1526.43	6.7616		1.2730	3.3631	4.9828
	15	1626.71	6.7306		1.2634	3.3445	5.0236
	16	1726.37	6.6998		1.2755	3.3270	5.0639
	17	1825.34	6.6667		1.2321	3.3093	5.1039
	18	1923.70	6.6305		1.2327	3.2948	5.1437
	19	2021.39	6.5943		1.2329	3.2825	5.1832
	20	2118.39	6.5554		1.2150	3.2683	5.2227
	21	2214.70	6.5159		1.2781	3.2531	5.2622
	22	2310.22	6.4786		1.3421	3.2391	5.3017
	23	2404.86	6.4409		1.4075	3.2256	5.3414
	24	2498.56	6.4052		1.5363	3.2121	5.3812
	25	2591.17	6.3716		1.6032	3.1997	5.4212
	26	2682.66	6.3354		1.6047	3.1888	5.4616
	27	2773.01	6.2959		1.5988	3.1780	5.5024
	28	2862.20	6.2521		1.5166	3.1666	5.5437
	29	2950.25	6.2008		1.4114	3.1553	5.5856
	30	3037.20	6.1452		1.4158	3.1446	5.6284
	31	3123.03	6.0911		1.4677	3.1342	5.6721
	32	3207.73	6.0399		1.5446	3.1241	5.7133
	33	3291.31	5.9921		1.7745	3.1144	5.7569
	34	3373.58	5.9411		2.1756	3.1049	5.8039
	35	3454.04	5.8733		2.4899	3.0958	5.8549
	36	3532.23	5.7959		2.2483	3.0871	5.9048
	37	3608.59	5.7434		1.5624	3.0787	5.9572
	38	3684.10	5.7047		1.5715	3.0706	5.9999
	39	3758.52	5.6293		2.0645	3.0626	6.0536
	40	3830.94	5.5305		2.1186	3.0550	6.1102
	41	3901.17	5.4268		2.6272	3.0477	6.1703
	42	3968.73	5.3102		2.5618	3.0408	6.2338
	43	4033.99	5.2300		2.4701	3.0342	6.3014
	44	4097.07	5.1035		3.7487	3.0278	6.3737
	45	4156.90	4.9903		2.5442	3.0219	6.4498
	46	4214.61	4.8790		4.0351	3.0162	6.5316
	47	4269.12	4.7421		2.9044	3.0109	6.6180
	48	4321.21	4.6206		4.0097	3.0058	6.7108
	49	4370.25	4.4761		3.0979	3.0011	6.8092
	50	4416.75	4.3367		4.1332	2.9967	6.9149
(3) $^4\Pi$	0	37.36	6.4451		1.9253	4.0542	4.4061
	1	111.60	6.3959		1.9058	3.9427	4.5541
	2	185.27	6.3476		1.8847	3.8706	4.6624
	3	258.40	6.3005		1.8635	3.8147	4.7545

4	331.01	6.2544		1.8398	3.7682	4.8370
5	403.12	6.2097		1.8131	3.7281	4.9130
6	474.78	6.1667		1.7803	3.6925	4.9841
7	546.04	6.1257		1.7458	3.6604	5.0511
8	616.95	6.0865		1.7138	3.6311	5.1149
9	687.55	6.0490		1.6788	3.6041	5.1759
10	757.86	6.0134		1.6414	3.5790	5.2343
11	827.95	5.9797		1.6044	3.5555	5.2906
12	897.84	5.9480		1.5648	3.5333	5.3448
13	967.58	5.9181		1.5278	3.5124	5.3971
14	1037.21	5.8901		1.4887	3.4925	5.4476
15	1106.75	5.8638		1.4550	3.4736	5.4966
16	1176.23	5.8390		1.4211	3.4554	5.5439
17	1245.68	5.8155		1.3947	3.4380	5.5899
18	1315.11	5.7926		1.3822	3.4213	5.6346
19	1384.48	5.7692		1.3879	3.4052	5.6779
20	1453.77	5.7442		1.4075	3.3897	5.7204
21	1522.88	5.7177		1.3951	3.3748	5.7680
22	1591.82	5.6940		1.2764	3.3603	5.8089
23	1660.78	5.6796		1.0736	3.3463	5.8445
24	1730.04	5.6739		0.98711	3.3327	5.8837
25	1799.69	5.6637		1.1793	3.3193	5.9222
26	1869.36	5.6353		1.4770	3.3059	5.9602
27	1938.54	5.5953		1.4402	3.2946	6.0034
28	2007.27	5.5683		1.0699	3.2844	6.0401
29	2076.05	5.5556		1.0216	3.2734	6.0769
30	2144.95	5.5350		1.1981	3.2615	6.1134
31	2213.72	5.5147		1.0135	3.2499	6.1494
32	2282.61	5.5077		9.8646	3.2388	6.1853
33	2351.65	5.4889		1.4445	3.2278	6.2210
34	2420.29	5.4576		1.4070	3.2171	6.2564
35	2488.61	5.4465		1.1007	3.2065	6.2915
36	2557.04	5.4391		1.3917	3.1965	6.3268
37	2625.24	5.4163		1.5315	3.1869	6.3620
38	2693.04	5.3962		1.3590	3.1773	6.3971
39	2760.60	5.3803		1.2355	3.1677	6.4322
40	2828.03	5.3629		1.2722	3.1581	6.4675
41	2895.17	5.3324		1.3831	3.1488	6.5029
42	2961.89	5.3079		0.96454	3.1397	6.5384
43	3028.56	5.2900		1.3245	3.1307	6.5743
44	3094.82	5.2512		1.4155	3.1218	6.6103
45	3160.64	5.2365		1.1338	3.1132	6.6465
46	3226.32	5.2101		1.8516	3.1047	6.6831
47	3291.32	5.1831		1.3665	3.0963	6.7198
48	3356.06	5.1697		1.7931	3.0881	6.7570
49	3420.23	5.1362		1.6111	3.0800	6.7945
50	3483.93	5.1188		1.5138	3.0720	6.8323
51	3547.18	5.0839		1.5392	3.0642	6.8706
52	3609.91	5.0566		1.3666	3.0565	6.9093
53	3672.17	5.0205		1.4268	3.0489	6.9486
54	3733.92	4.9894		1.5218	3.0415	6.9884
55	3795.11	4.9531		1.5306	3.0341	7.0288
56	3855.78	4.9245		1.8760	3.0269	7.0698

	57	3915.75	4.8884		1.7012	3.0197	7.1114
	58	3975.19	4.8621		2.1628	3.0127	7.1537
	59	4033.83	4.8238		1.6627	3.0058	7.1967
	60	4091.94	4.7942		2.2376	2.9990	7.2405
	61	4149.20	4.7530		1.4486	2.9923	7.2850
	62	4205.93	4.7147		2.2358	2.9857	7.3306
	63	4261.76	4.6744		1.3854	2.9792	7.3769
	64	4317.04	4.6263		2.2845	2.9728	7.4244
	65	4371.39	4.5918		1.7297	2.9666	7.4729
	66	4425.06	4.5363		2.2241	2.9604	7.5226
	67	4477.86	4.5047		2.3624	2.9544	7.5735
	68	4529.77	4.4488		1.9610	2.9485	7.6256
	69	4580.90	4.4072		2.7604	2.9427	7.6792
	70	4631.00	4.3589		1.8987	2.9370	7.7342
	71	4680.30	4.2998		2.4609	2.9315	7.7909
	72	4728.59	4.2542		2.3838	2.9261	7.8493
	73	4775.90	4.1904		1.9968	2.9208	7.9095
	74	4822.29	4.1320		2.7019	2.9157	7.9719
	75	4867.57	4.0751		2.4235	2.9107	8.0362
	76	4911.80	4.0051		2.5301	2.9058	8.1030
	77	4954.91	3.9393		3.0726	2.9011	8.1764
	78	5037.47	3.7997		2.6265	2.8921	8.3228
	79	5077.08	3.7370		2.8328	2.8879	8.4008
	80	5115.59	3.6667		3.0554	2.8838	8.4826
	81	5152.81	3.5818		3.0145	2.8798	8.5680
	82	5188.73	3.5049		2.8097	2.8760	8.6576
	83	5223.46	3.4244		3.2521	2.8723	8.7519
	84	5288.83	3.2503		3.2949	2.8655	8.9556
	85	5319.49	3.1540		3.4387	2.8623	9.0662
(1) $^4\Sigma^-$	0	77.00	11.074		2.1603	3.1090	3.3547
	1	235.42	11.058		1.8641	3.0308	3.4438
	2	399.13	11.022		2.2058	3.5124	3.5124
	3	559.44	10.970		1.8913	2.9369	3.5696
	4	720.80	10.936		2.2075	2.9022	3.6214
	5	879.93	10.885		1.8904	2.8722	3.6686
	6	1039.36	10.845		2.2223	2.8452	3.7132
	7	1196.90	10.799		1.8739	2.8208	3.7551
	8	1354.49	10.749		2.1926	2.7984	3.7955
	9	1510.52	10.710		1.8903	2.7778	3.8342
	10	1666.27	10.648		2.0696	2.7601	3.8718
	11	1820.99	10.613		2.0203	2.7418	3.9083
	12	1974.85	10.552		1.9517	2.7245	3.9440
	13	2128.08	10.516		2.2634	2.7082	3.9789
	14	2279.89	10.472		1.9893	2.6927	4.0129
	15	2431.04	10.427		2.3107	2.6780	4.0465
	16	2580.83	10.396		2.0893	2.6639	4.0794
	17	2729.74	10.339		1.9602	2.6505	4.1119
	18	2877.94	10.296		2.1463	2.6375	4.1440
	19	3025.07	10.245		1.8446	2.6251	4.1757
	20	3171.64	10.190		2.1874	2.6132	4.2071
	21	3317.14	10.157		2.2339	2.6016	4.2382
	22	3461.55	10.107		2.0057	2.5905	4.2690
	23	3605.20	10.062		2.2318	2.5798	4.2996

24	3747.76	10.018		1.9400	2.5694	4.3300
25	3889.55	9.9585		1.9089	2.5593	4.3602
26	4030.61	9.9135		2.2725	2.5495	4.3903
27	4170.56	9.8717		2.0808	2.5400	4.4202
28	4309.64	9.8200		2.1125	2.5308	4.4500
29	4447.80	9.7781		2.2241	2.5219	4.4797
30	4584.93	9.7288		1.8679	2.5132	4.5092
31	4721.32	9.6702		1.9823	2.5047	4.5387
32	4856.87	9.6257		2.2749	2.4965	4.5682
33	4991.35	9.5812		2.0801	2.4884	4.5976
34	5124.96	9.5290		2.0967	2.4806	4.6269
35	5257.66	9.4838		2.1992	2.4730	4.6562
36	5389.36	9.4343		1.9128	2.4656	4.6855
37	5520.25	9.3764		1.9437	2.4583	4.7148
38	5650.30	9.3284		2.2747	2.4512	4.7441
39	5779.32	9.2849		2.1721	2.4443	4.7734
40	5907.40	9.2330		2.0041	2.4375	4.8027
41	6034.62	9.1820		2.1249	2.4309	4.8320
42	6160.90	9.1337		2.0553	2.4244	4.8614
43	6286.26	9.0791		1.9188	2.4181	4.8908
44	6410.77	9.0248		2.1474	2.4119	4.9203
45	6534.33	8.9784		2.3136	2.4058	4.9499
46	6656.84	8.9295		2.0928	2.3999	4.9796
47	6778.42	8.8732		1.9733	2.3941	5.0094
48	6899.08	8.8183		2.1126	2.3884	5.0393
49	7018.77	8.7663		2.1276	2.3828	5.0694
50	7137.46	8.7111		2.0532	2.3774	5.0997
51	7255.20	8.6556		2.1817	2.3720	5.1300
52	7371.94	8.6043		2.2912	2.3668	5.1606
53	7487.63	8.5515		2.1245	2.3617	5.1913
54	7602.31	8.4926		1.9721	2.3567	5.2223
55	7716.02	8.4328		2.0963	2.3518	5.2534
56	7940.33	8.3217		2.2220	2.3422	5.3165
57	8050.89	8.2631		2.1595	2.3376	5.3483
58	8160.40	8.2037		2.2182	2.3330	5.3805
59	8268.82	8.1452		2.2488	2.3286	5.4130
60	8376.14	8.0849		2.1550	2.3242	5.4458
61	8482.35	8.0211		2.1176	2.3200	5.4789
62	8587.47	7.9571		2.2503	2.3158	5.5125
63	8691.44	7.8954		2.4009	2.3117	5.5464
64	8794.22	7.8333		2.3841	2.3077	5.5807
65	8895.79	7.7675		2.2927	2.3038	5.6156
66	8996.14	7.6969		2.3222	2.2999	5.6509
67	9095.18	7.6225		2.5111	2.2961	5.6868
68	9192.77	7.5420		2.7201	2.2925	5.7242
69	9288.73	7.4555		2.7453	2.2889	5.7664
70	9383.05	7.3736		2.4083	2.2854	5.8042
71	9476.10	7.3149		1.8910	2.2820	5.8390
72	9568.42	7.2745		1.9318	2.2786	5.8781
73	9659.98	7.2106		2.8870	2.2753	5.9183
74	9749.82	7.0951		3.6733	2.2720	5.9594
75	9837.16	6.9738		2.9039	2.2689	6.0081
76	9922.66	6.8945		2.3927	2.2659	6.0511

77	10006.78	6.8050		3.1683	2.2629	6.0957
78	10089.09	6.7145		2.3541	2.2601	6.1413

Table S4: Table of the FCF for the transitions $X^2\Sigma^+ - (1)^2\Pi$, $X^2\Sigma^+ - (2)^2\Pi$, $X^2\Sigma^+ - (2)^2\Sigma^+$, $(1)^2\Pi - (2)^2\Pi$, $(2)^2\Sigma^+ - (2)^2\Pi$, $(2)^2\Sigma^+ - (1)^2\Pi$ of the molecule CaCs.

$(1)^2\Pi - (2)^2\Pi$						
	0	1	2	3	4	5
0	4.08E-07	6.04E-06	4.48E-05	2.21E-04	8.18E-04	2.42E-03
1	4.59E-06	6.02E-05	3.92E-04	1.67E-03	5.28E-03	1.31E-02
2	2.63E-05	3.04E-04	1.72E-03	6.28E-03	1.66E-02	3.37E-02
3	1.04E-04	1.06E-03	5.16E-03	1.59E-02	3.47E-02	5.53E-02
4	3.24E-04	2.86E-03	1.19E-02	3.05E-02	5.28E-02	6.27E-02
5	8.33E-04	6.35E-03	2.23E-02	4.62E-02	6.08E-02	4.81E-02
$(2)^2\Sigma^+ - (2)^2\Pi$						
0	8.02E-01	1.48E-01	4.09E-02	7.37E-03	1.34E-03	2.09E-04
1	1.89E-01	4.56E-01	2.17E-01	1.02E-01	2.75E-02	7.12E-03
2	8.41E-03	3.51E-01	1.88E-01	2.02E-01	1.61E-01	6.24E-02
3	2.90E-04	4.28E-02	4.36E-01	3.76E-02	1.28E-01	1.87E-01
4	4.59E-04	4.92E-06	1.12E-01	4.20E-01	2.07E-04	4.81E-02
5	5.32E-05	1.75E-03	1.69E-03	2.09E-01	3.16E-01	2.74E-02
$X^2\Sigma^+ - (1)^2\Pi$						
0	4.78E-07	4.77E-06	2.48E-05	8.97E-05	2.55E-04	6.05E-04
1	7.08E-06	6.29E-05	2.90E-04	9.27E-04	2.32E-03	4.83E-03
2	5.24E-05	4.10E-04	1.66E-03	4.61E-03	9.97E-03	1.78E-02
3	2.57E-04	1.75E-03	6.08E-03	1.44E-02	2.63E-02	3.89E-02
4	9.46E-04	5.50E-03	1.62E-02	3.19E-02	4.72E-02	5.47E-02
5	2.77E-03	1.35E-02	3.28E-02	5.17E-02	5.83E-02	4.77E-02
$X^2\Sigma^+ - (2)^2\Pi$						
0	0.9.9901	4.72E-04	1.04E-04	8.86E-06	2.00E-06	2.06E-08
1	4.71E-04	9.99E-01	4.23E-07	3.81E-04	5.96E-06	6.58E-06
2	1.02E-04	4.20E-08	9.98E-01	1.11E-03	7.69E-04	3.53E-06
3	8.89E-06	3.76E-04	9.53E-04	9.91E-01	6.55E-03	1.20E-03
4	8.86E-07	1.77E-05	8.82E-04	5.49E-03	9.72E-01	2.05E-02
5	3.16E-08	2.16E-06	5.86E-05	1.94E-03	1.65E-02	9.32E-01
$X^2\Sigma^+ - (2)^2\Sigma^+$						
0	8.21E-01	1.72E-01	6.84E-03	3.40E-04	4.75E-04	6.86E-05
1	1.33E-01	4.73E-01	3.48E-01	4.41E-02	1.07E-05	1.68E-03
2	3.77E-02	2.10E-01	1.76E-01	4.35E-01	1.31E-01	5.12E-03
3	7.14E-03	1.03E-01	1.92E-01	2.02E-02	3.82E-01	2.52E-01
4	1.47E-03	3.05E-02	1.64E-01	1.04E-01	9.23E-03	2.20E-01
5	2.65E-04	8.86E-03	7.25E-02	1.78E-01	1.93E-02	5.95E-02
$(2)^2\Sigma^+ - (1)^2\Pi$						
0	1.68E-06	1.85E-05	1.02E-04	3.94E-04	1.17E-03	2.85E-03
1	2.60E-05	2.45E-04	1.16E-03	3.76E-03	9.30E-03	1.87E-02
2	1.97E-04	1.57E-03	6.19E-03	1.65E-02	3.28E-02	5.18E-02
3	9.71E-04	6.39E-03	2.04E-02	4.28E-02	6.48E-02	7.35E-02

79	10170.25	6.6612		2.4586	2.2573	6.1884

[a] Present work using MRCI method.

[c] Ref. [16].

4	3.52E-03	1.86E-02	4.63E-02	7.21E-02	7.47E-02	4.95E-02
5	9.92E-03	4.08E-02	7.47E-02	7.76E-02	4.31E-02	6.81E-03
